# Comprehensive Evaluation of PM (Deposition/TSP) Pollution from Multiple Quarrying Activities

**DOI:** 10.1021/acsomega.4c11520

**Published:** 2025-05-13

**Authors:** Gülnihal Kara, Ali Çankaya

**Affiliations:** † 531804 Konya Technical University, Department of Environmental Engineering, Konya 42130, Turkey; ‡ Institution of Graduate Education, Department of Environmental Engineering, Konya Technical University, Konya 42130, Turkey

## Abstract

This study, for the first time, analyzed organic matter (OM)-ash fractions, inorganic/organic species, chemical structures, and inhibition effects (EI) in the same sample using a novel procedure. This allowed for an evaluation of the actual contributions of these species to particulate matter (PM) and environmental impacts. Results showed that the mobilities of Al, Co, and potentially toxic elements (PTEs) (As, Cd, Cr, Cu, Pb, and Ni) varied between organic and inorganic phases depending on pH and organic groups. The transition of these elements into the organic phase, along with Ca, contributed to silica polymerization, increased EI in the organic eluate, and enhanced bioavailability in the presence of low water-soluble OM (WOM). Therefore, although Ca abundance reduced EI in the organic eluate, the organic eluate of multiple quarrying areas (MQA), with the lowest OM, exhibited an EI equivalent to the background area (BA). Although ΣPTEs in total suspended particulates (TSP) inorganic eluates decreased compared to total deposition (TD), EI increased. Due to TD-facilitated accumulation-transition and metabolite formation, leading to higher EI in organic eluates. This finding aligned with similar effects observed in aged atmospheric aerosols from the same region. MQA activities significantly contributed to inert dust pollution, Ca, Cu, Cr, ^40^K, and Na accumulation, silica and alkali oxide abundance, SOA precursor (e.g., phthalic acid) transformation into more hazardous phthalate esters (PAEs), and new metabolite formation affecting climate change. These activities increased yearly TD and estimated mean TSP by 42 and 41 times, respectively, to 52.9 g/m^2^-year and 1.3 mg/m^3^ compared to BA. Despite MQA having the highest TSP-TD, semirural (SRRA) PM influenced by MQA exhibited the highest chemical composition and acute EI. Furthermore, it was found that, aside from Na and Se, there were no significantly enriched elements in MQA when compared to BA.

## Graphical abstract

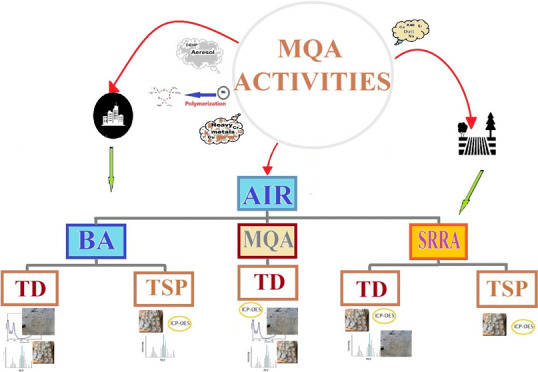

## 1 Introduction

The growth of the global population, infrastructure development, and the increasing demand driven by factors such as climate change have made the cement industry the second-largest sector,^1–3^ leading to a rise in the production of its primary raw material, limestone.^4^ Limestone production quarries generate dust and gas emissions during their operational processes (excavation/lifting, loading/unloading, crushing-screening, internal/external transportation, use of explosives, etc.), along with exhaust gases from mining machinery and emissions from maintenance activities (washing, lubrication, etc.^5^), contributing to air pollution. These activities also lead to the consumption, depletion, and reduction of both underground and surface water capacity, as well as local changes in flow regimes. Other environmental impacts include noise pollution, the loss of natural wind barriers, local climate changes, the loss of agricultural land, negative landscape ecology, vibration, destruction of nearby buildings, deterioration of transportation routes, and/or erosion, as well as traffic accidents and workplace hazards. Furthermore, health risks such as silicosis are present, causing discomfort to the local population.^3,6^ During operations, negative effects on air, water, and soil quality, as well as on natural vegetation, agricultural products, wildlife, and biodiversity, lead to health problems and migration.^3,7,8^ After operations are completed, these activities result in negative landscape changes (e.g., visual degradation of stockpiled areas) and an increased risk of drowning in unrehabilitated stockpiles.

Today, urban centers are heavily impacted by the unavoidable effects of PM pollution. Similar atmospheric interactions may occur in TD samples, offering more realistic results in environmental impact assessments.^9^ Quarry activities are among the most significant sources of visible PM pollution.

One of the aims of this study is to investigate the relationship between high PM concentrations in MQA, the chemical composition of PM, and its relationship with the EI, as well as to examine the effects of significant pollution sources on these high concentrations and identify pollutants and their sources that could adversely affect environmental and human health due to various activities. Comprehensively investigated PM pollution, the environmental impacts of which are not fully understood, stemming from the MQA in the region. To assess the effects and the true scale of the issue, comparisons were made with SRRA (believed to be adversely affected by MQA) and BA, an area distant from point sources. Fieldwork for sample collection was conducted in December and January of 2022. The physicochemical properties of the samples, their organic-elemental compositions, and the acute EIs of inorganic/organic eluates were determined and correlated through statistical analysis. In this context, the chemical compositions of BA, SRRA, and MQA-PM each equally impacted by emissions from international airport and road maintenance service were compared with acute EI levels. The effects of MQA and suspected drivers on EIs and the chemical composition of BA and on SRRA-PMs were investigated. Another objective was to investigate the effects of MQA on suspected SOA drivers, aerosol aging, and the formation of new metabolites in PM in the city center under high-altitude, cold climate conditions. For this purpose, chemical species in TSPs-TDs and acute EIs were compared. New metabolites formed as a result of chemical modifications leading to EI formation were identified.

To the best of our knowledge, this is the first report in which MQA-related pollution is investigated holistically, cMSs signals are identified in agricultural/quarry PM, and newly modified metabolites from chemical transformations are verified, offering a new perspective in understanding potential risks.

## 2 Methods and Materials

### 2.1 Study Area

The study was conducted in Konya, Turkey’s 10th most populous province (2.4 million, 2021), known as the ″grain silo″ due to its economy, which is primarily based on agriculture, alongside livestock farming, automotive, food, and textile industries.^17^ The city hosts 2 organized industrial zones (OIZs), 705 firms with emission licenses, and 167 industrial facilities classified as high-pollution sources. Industrial activities are concentrated in the northern part of the city, where the study was also carried out.^10,11^ In 2022, the national PM_10_ limit value (50 μg/m^3^) was exceeded 53 times in the city center and 20 times in BA. Air pollution has frequently been reported in local media.^12^ Surrounded by mountains exceeding 2000 m in height, the city has a high altitude conducive to SOA formation^13^ and organic aerosol aging,^14^ as well as cold climate conditions. The region contains Pb and Zn reserves and volcanic rocks with widespread Ni, Cr, and Mn content.^15^ The quarries in the region, due to intensified dust and noise emissions during dry and windy periods and the resulting damage to buildings, have become a frequent subject of protests by residents who rely on agriculture and livestock farming. These protests, often covered by the local media, are accompanied by complaints from drivers using the nearby D715 highway.^16^ The expansion of zoning plan boundaries has caused some quarries to fall within residential areas, exacerbating their negative impacts and affecting a larger portion of the population. In this study, three strategic sampling points were selected (Figure 1), located approximately 20 km from the city center and 5–10 km from major pollution sources such as the airport, industrial zone, bypass road, and wastewater treatment plant (WWTP). Although these points are situated against the prevailing wind direction, they are still influenced by these sources. One of the sampling points is located in the area containing 10 out of the 75 quarries in the city, which operates a stepped system (MQA), while another is within the nearest settlement area (SRRA). These two sampling points are near a maintenance unit with heavy vehicle traffic supplying materials for a bypass road under construction (10,069 crossings in 2022, including 1115 HDVs) and are about 5 km from the unit. The MQA, SRRA, and BA sampling points are located 0.36, 1.2, and 17.6 km away from multiple quarry activities, respectively.

**Figure 1:**
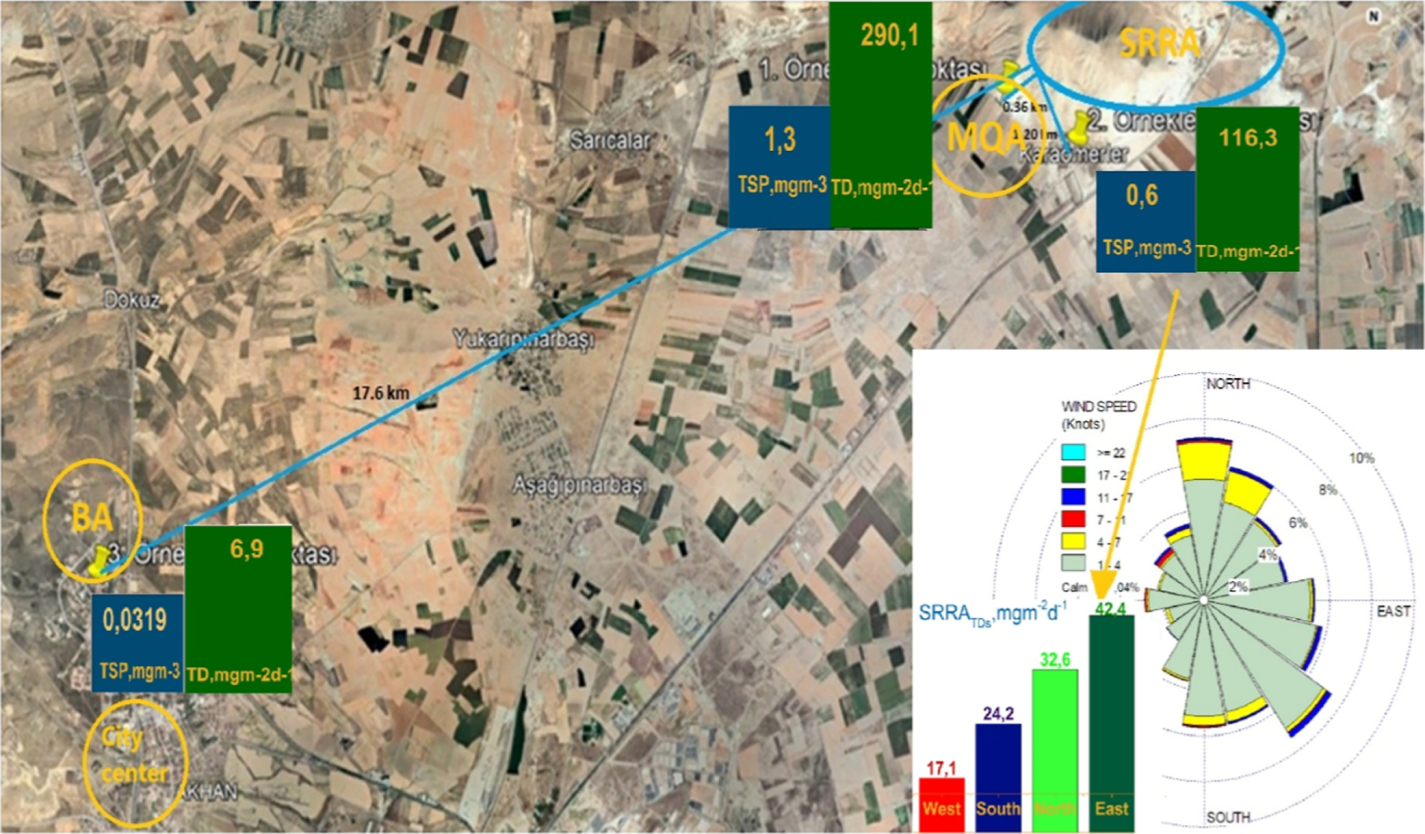
Site location and sampling points, TDs-TSPs, and prevailing winds during the sampling.

### 2.2 Sampling Procedure

The MQA was not sampled within its site, but in the surrounding area, while both TD and TSP samples were simultaneously collected at the other two sampling locations (Figure 1). The TDs were collected for 30 days using a simple system in accordance with Turkey Environmental Regulations (TS-2342), while TSP samples were collected for 24 h, twice, using the Gast medium-volume sampler. Both sampling procedures were carried out according to the protocol outlined in Supporting Information-Section 1 (S1). Meteorological data (e.g., air temperature, pressure) obtained from the 37°52′07.3″N 32°28′16.7″E station (presented in Supporting Information-S2) were used to generate the wind diagram (Figure 1).

### 2.3 Sample Preparation, Extraction and Physicochemical Characterization

All materials in contact with the samples were subjected to the CEREVE procedure, the details of which are provided in Supporting Information-S3. Pre and postsampling, validation, physicochemical analysis, FTIR, ICP-OES and GC/MS procedures are presented in Supporting Information-S4. Using the newly applied procedure in this study, 12 different analyses (pH, EC, flux, total dissolved solids (TDS), total suspended solids (TSS), The loss on ignition (LOIs), extractable organic matter (EOM), FTIR, ICP-OES, GC/MS, EIs) were performed on a single TD sample, and 5 different analyses (mass, EOM, FTIR, ICP-OES, GC/MS, EIs) were performed on TSP. This approach allowed for the evaluation of the contributions of different species to PM and enabled reliable correlation analyses (using direct sample data rather than sampling point data). For this procedure, TD samples were combined with the rinse residues after measuring volume, EC, and pH, then stirred for 1 min using a magnetic stirrer. Half the volume was filtered through a GF/A filter for ICP-OES and GC/MS, while the rest passed through an ash-free cellulose filter for LOI-TDS/TSS analysis. The GF/A filters were dried in a freezer to prevent losses of volatile compounds (VOC),^18^ and the cellulose filters were dried in an oven. After weighing, 0.01 g samples were taken for FTIR analysis. Four equal segments were cut from the GF/A filter using ceramic scissors (1/2 for element analysis, 1 for organic, and 1 for inorganic eluates). The same procedure was repeated for blank filters. Using the ultrasonic- extraction method detailed in Supporting Information-S5, organic and inorganic eluates were prepared sequentially with dichloromethane (DCM) and ultrapure water (UPW). The DCM extracts were concentrated to 1 mL at 35 °C under reduced pressure using an evaporator. For the EOM analysis, the DCM was evaporated (using a water bath), and after weighing, the sample was redissolved in 1 mL of DCM. Half of the solution was used for phytotoxicity testing, while the remainder was prepared for GC/MS analysis and stored at −18 °C. Prior to the phytotoxicity test, the DCM was evaporated again, and the extract was redissolved in 1.5% (v/v) dimethyl sulfoxide (DMSO)/UPW for solvent exchange.^19^ Organic and inorganic eluates were prepared in serial dilutions (ranging from 6.25% to 100%, including six different concentrations) and controls (six replicates per test). FTIR, LOI results, and the EC-TDS relationship indicated the presence of VOCs, so cleanup procedures were not applied to the GC/MS extracts.

### 2.4 Ecotoxicity Analysis

The test involving L. sativum was conducted according to^20^ and^21^ (details provided in Supporting Information-S6). Biometric indices, including the seed germination index and percentage inhibition, were used for the evaluation of the results.^22^

### 2.5 Instrumental Analysis

FTIR, ICP-OES, and GC/MS analyses were performed according to the procedures detailed in Supporting Information-S7. EFs relative to the urban background were calculated using Al as the reference element (RE), following.^23^ Similarly, most previous studies have also employed Al as the RE.^23,24^ EFs were classified into three categories: <3 as low enrichment, 3–5 as moderate enrichment, and >5 as high enrichment.^25^

### 2.6 Validation Procedure

In this study, a multistep validation procedure was conducted, details of which are provided in Supporting Information-S8. For elemental analyses, a three-step validation was employed, incorporating SRM, laboratory (LB)/field (FB) blanks, and the internal standard (IS) technique. For organic species, a two-step validation using the LB/FB technique was applied. For determining phytotoxic effects, a two-step validation comprising control and reference tests was performed.

## 3 Results

### 3.1 TSP Mass Concentrations, Physicochemical Characterization of TDs, and Fluxes

TSP, flux, and physicochemical analysis results, including pH, EC, TDS, TS, EOM, and LOI, are presented in Table 1. This study observed that both EQSs for Turkey (450 mg/m^2^-day), and EEA limits (350 mg/m^2^-day) were not exceeded in MQA_TD_ (Table 1).^26,27^

**Table 1:** TSP, Flux, and Physicochemical Analysis Results

	BA	MQA	SRRA
TD volume (mL)	750^a^	368^a^	300^a^
TD flux (mg/m^2^-day)	6.95^a^	290.17^a^	116.32^a^
TSP (μg/m^3^)	32.78^a^	1199[Table-fn t1fn3]	599.96^a^
pH	7.39^a^	8.17^a^	NA
EC (μS/cm)	202.50^a^	348^a^	NA
TDS (g/L)	0.24^a^	0.12^a^	0.09^a^
TSS (g/L)	0.08^a^	1.79^a^	0.15^a^
insoluble OM (WIOM) (g/g)	0.40^a^	0.13^a^	0.34^a^
WOM (g/g)	0.34^a^	0.04^a^	0.17^a^
water-soluble ash (WSA) (g/g)	0.25^a^	0.07^a^	0.20^a^
water insoluble ash (WIA) (g/g)	0.02^a^	0.75^a^	0.30^a^
EOM_TD_ (g/g)	0.17^a^	0.07^a^	0.31^a^
EOM_TSP_ (g/g)	0.21^a^	NA	0.32^a^
EITD-inorganic eluat (%)	–15.33[Table-fn t1fn3]	2.55[Table-fn t1fn3]	10.00[Table-fn t1fn3]
EITD-organic eluat (%)	17.52[Table-fn t1fn3]	12.00[Table-fn t1fn3]	21.15[Table-fn t1fn3]
EITSP- inorganic eluat (%)	13.87[Table-fn t1fn3]	NA	5.73[Table-fn t1fn3]
EITSP- organic eluat (%)	8.37[Table-fn t1fn3]	NA	4.10[Table-fn t1fn3]

a The average of 2 sets.

b Highest inhibitory/bioavailability effect.

The spatial distribution of dust in the MQA, along with the influence of wind direction, was evaluated using the wind diagram and the results of the TS-2342 sampler with four nozzles in SRRA (Supporting Information-S1 and Figure 1). According to the diagram, the sampling region is located at the intersection of dominant wind directions, and while the winds are frequent, their strength is low. During the sampling period, with only 5 days of strong winds (>3 m/s) reported (Supporting Information-S2 and Table 1). The highest TD flux was observed at the east nozzle, aligned with the dominant wind direction, and was 1.5, 1.8, and 2.5 times higher than those from the north, south, and west, respectively.

Blasting activities in quarries primarily generate large PM (>10 μm, 80%^28^). In this study, the contribution of TDS to TS was found to be three times higher in SRRA_TD_ (36.4%) compared to MQA (11.8%). These findings indicate the dominant presence of fine particles in the dust transported to and accumulated in SRRA. The results from Atasağun^29^ for MQA also support this assumption. Over the four-month period (50 PM_10_ and 46 PM_2.5_ samples), PM_10_ showed variability (±32), whereas PM_2.5_ exhibited consistent monthly (±11) and maximum daily averages. Furthermore, PM_10_ levels decreased by 5% in August (a period of reduced quarry activities due to a nine-day religious holiday) compared to September, while PM_2.5_ increased by 21%. This confirms that quarrying mainly affects large air particles, while secondary aerosol formation and other sources impact fine particle levels. The TD/TSP ratios in SRRA and BA, Sivacoumar et al.^30^’s PM_10_-TSP results (cont. monit. syst., Σ 555), the TD flux in MQA, and Atasağun^29^’s PM_10_ results were used to estimate MQA_TSP_. Using the stable PM_10_/TSP ratios (0.21–0.26) reported by and the PM_10_ results of Atasağun,^29^ the max daily TSP in MQA (TSP_MQA_) was estimated to be 1.46 mg/m^3^. The TD flux in MQA was approximately twice as high as in SRRA, while TD in SRRA was 17 times and TSP was 18 times higher than in BA. The TD/TSP ratios in SRRA and BA showed minimal variability, with values of 20.62 and 18.37, respectively, and an average of 19.5. Using the TD_MQA_/TD_SRRA_ and TD_MQA_/TSP_MQA_ ratios (2 and 20, respectively), the TSP concentrations in MQA were estimated to be 1.2 and 1.4 mg/m^3^. Consistent results were obtained using all three methods (1.2, 1.4, and 1.5 mg/m^3^). In the literature, TSPs in mining activities are reported to vary depending on the sampling location, activity type, and material involved. For example, max. TSP of 0.88 mg/m^3^ were observed in surface limestone mining areas,^31^ avg. of 0.22 mg/m^3^ in stone crushing facilities,^32^ avg. of 0.47 mg/m^3^ in stone mine facilities,^33^ and of 15.5–28.4 mg/m^3^ in small-scale mining during tasks such as drilling and blasting.^34^ In quarry operations involving shovelling and loading, avg. TSP was reported as 4.3 mg/m^3^.^35^ In this study, the estimated TSP in MQA was found to be ∼3–6 times higher than that in stone crushing facilities^32,33^ and 1.5 times higher than that in surface limestone mining areas.^31^ The presence of and other alkaline salts in MQA contributed to the weak alkalinity of the TD and, despite the increase in pH, led to a rise in EC. The pH and EC values of the TDs analyzed in this study (Table 1) were similar to those reported for a limited number of TD samples in the literature, such as in Poland (max pH 7.9, EC 30.7–897 μS/cm;^36^) and Italy (max pH 8.4, mean EC 117 μS/cm, and max EC 920 μS/cm;.^37^ Compared to BA, the EC, TDS, and TA values of MQA were 1.4, 2.5, and 3.5 times higher, respectively. This increase was likely due to the higher concentrations of Ca (HCO_3_)_2_ and other elements (e.g., Na, Cu), as well as NO_3_/Cl-containing salts and/or organo-complexes, in MQA compared to BA. The EC-TDS relationship (*k*-factor), linked to dissolved ions, offered key insights for estimating species composition and choosing GC/MS methods. The lower values of the *k* factor in this study, being 0.34 for MQA and 0.23 for BA, indicated the presence of carboxylic acids in BA and hydroxyl-containing species such as phenols in MQA.^38^ In alkaline pH, NH4 salts (from MQA blasting) convert to NH3 gas, HCO3 salts to CO3 crystals, and cations/metals to insoluble organo-complexes, likely lowering TDS and the *k*-factor. As the contributions of WIOM to the OM in SRRA_TD_, BA_TD_, and MQA increased (67%, 55%, and 37%, respectively), the EOM (0.31, 0.17, and 0.07 g/g, respectively) increased proportionally. The similar WIOM content in SRRA_TD_ to that of EOM, which aligns with the GC/MS results, indicates the predominant presence of insoluble organic species and confirms that all species present are extractable. These results show that WIOM-EOM alignment with GC/MS signal intensity supports WIOM as a cost-effective, simple complementary method to GC/MS analysis.

### 3.2 GC/MS-Nontargeted Analysis

According to Black et al.^13^ understanding the physicochemical properties of samples (e.g., pH, OM etc.) and implementing improvements based on the obtained results can significantly contribute to the expansion of chemical domain boundaries in NTA studies. In this study, the extraction and analysis procedure (Supporting Information-S9) was developed based on the physicochemical and FTIR results of the samples, aiming to extend the chemical domain boundaries. A total of 23 organic compounds with sample-to-blank ratios greater than 5 were detected in GC/MS spectra: 5 in BA_TSP_, 4 in BA_TD_, 12 in SRRA_TD_, and 4 in MQA (Table 2). The highest total SI (total area) value was observed in SRRA_TD_, which belonged to the cMSs group, with a global production volume reaching 2.4 million metric tons in 2020. MQA and BA_TD_ were observed to have similar SI values (The EOM in BA_TD_ was found to be twice as high as that in MQA). The SI value in BA’s TSP was ten times greater than that in its TD. Moreover, different organic species were detected in the TD and TSP. The number of shared species across the three sampling sites, equidistant from point sources, was found to be limited (5 cMSs species (D4–D6, D8, D10) in BA_TSP_ and SRRA_TD_, and D7 and DEHP in MQA and BA_TD_). The ranking of the cMSs species in terms of abundance also varied in SRRA_TD_ and BA_TSP_, except for D6, D8, and D10. It is hypothesized that secondary organosiloxane aerosol (SOSiA) and gaseous cMSs adsorbed onto fine particles contributed to the higher diversity and quantity of species in the TSP.

**Table 2:** Results of the GC–MS (Full Scan) Analysis of Samples

	BA_TD_ (SI × 10^8^)	BA_TSP_ (SI × 10^9^)	SRRA_TD_ (SI × 10^10^)	MQA_TD_ (SI × 10^8^)
D4		0.004	0.020	1530 × 10^–7^
D5		0.005	0.010	
D6		0.036	0.029	
D7	0.002		0.019	0.006
D8		0.007	0.004	
D9			0.001	
D10		0.002	0.001	
ΣcMSs	0.002	0.054	0.083	0.006
D4/D5		0.861	2035	
PD (per/km^2^)	2758.62	2758.62	4.00	≪
PA	0.012			
dibutyl phthalate (DBP)	0.008			
(DEHP)	0.029			0.035
ΣPAEs	0.037			0.035
2,4-bis(1-phenylethyl) phenol				0.028
C22			0.001	
C21			0.002	
C29			0.005	
C30			0.005	
C20			0.005	
C18			0.004	
C18/C29			0.768	
Σalkanes			0.789	

### 3.3 cMSs

D3 in none of the samples, and D4–5 was not detected in MQA and BA. Many researchers have reported higher levels of D5, D6 (correlations with population density (PD), 0.6 and 0.7, respectively;^39,40^ and D4 in urban areas compared to rural regions. In contrast, this study found the highest SI values for all three compounds in SRRA_TD_, which had the lowest PD (Table 2).

Furthermore, all three sampling points were equidistant from major sources of cMSs, including airport (D3–D5), landfill (D5–D6), WWTP (D4–D6), and industrial facilities, particularly paper factories, (significant users of high-volume foam suppressants (D6).^41^ Previous studies have consistently reported that among cMSs, D5 is the most frequently and abundantly detected in environmental matrices, while D4 is most commonly observed in air (in the gas phase). According to the SI results, D4 ranked as the second highest in SRRA_TD_ and the third highest in BA_TSP_ and was the most widely detected compound across the majority of samples. Personal Care Products (PCHP) due to impurities (not directly used,^39^) and serves as a primary monomer in the silicone industry. In previous studies, D4 was reported at higher levels in urban centers, its highest SI value was surprisingly observed in SRRA_TD_, the area with the lowest PD. Similarly, Horii et al.^39^ also reported higher levels of D4 in rural areas with monomer production facilities (where D4 constitutes 80% of ΣcMSs,^39^) compared to urban centers. The province hosts companies producing household goods, building materials, and chemicals, contributing 50% of global cMS production, plus a paper factory. PDMS-based high-temperature greases are widely used in industries like lime/hardening furnaces and bearing lubrication. The industries at all three sampling sites, including the airport (where thermal degradation of silicone oils commonly used in the aircraft industry results in D4, D3, and D5 at 100%, 33%, and 18%, respectively,^42^), maintenance facilities (where petrochemicals and petroleum products contain D4,^41^), and the storage areas for these products, can be considered as common sources due to the high contribution of D4 within ΣcMSs (D4 and D6, 58% and 33%, respectively,^43^). The increase in D5 and D6 alongside D4 in SRRA_TD_, along with the presence of other cMSs, confirms the strong influence of local sources, consistent with the LOI results. Another surprising finding was that D6 had the highest SI, followed by D5 in second place. The peak area of D6 at the same concentration was half that of D4 and like D5,^44^ which indicates that the ranking of sizes did not change according to concentration. Similarly, Anh et al.^45^ reported D6 as the cMSs, with the highest concentration in the particle phase, which was even twice as high as in the gas phase. Recent studies have reported that D6 tends to continuously increase in the atmosphere due to industrial activities, WWTPs, and landfills, and that it accumulates.^46^ The atmospheric half-life of D6 is estimated to be 3.3 days, while its half-life in sediment is 5836 days.^47,48^ Compared to D4, the atmospheric half-life of D6 is 3.5 times shorter, but its half-life in sediment is 16 times longer. Experimental data obtained by Panagopoulos and MacLeod,^48^ confirmed the accumulation of D6 in sediments. The higher fraction of D6 observed in all PM samples from four different sampling locations in Vietnam (PM0.5 > 0.1,^49^ cannot be linked to SOA, and its presence in the gas phase in polar regions^50,51^ indicates that D6 can be transported over long distances. Additionally, the presence of D6 in environmental matrices^52^ and in polar regions further confirms its widespread use, along with D3 and D5. Previous studies may have detected D6 at lower levels in the gas phase compared to D5 due to its tendency to be present in the particulate phase and/or artifacts in the sorbents used.

Like other cMSs, D6 was also found with the highest SI in SRRA_TD_. Similarly, Le et al.^49^ reported that in the craft village, which had the lowest particulate concentration, D6 was higher than D4–5 across all particulate fractions (PM0.5–0.1). At the same sampling site (BA), its presence in TSP, rather than TD, confirmed the strong sorption of D6 to suspended particles, while its absence in TD (except SRRA) validated the strong influence of local sources. In the BA_TSP_, when landfill and WWTP are considered as common sources for D6 and D5, the observed D6 > D5 ratio may have been influenced by sorption factors, as well as the proximity of the paper factory and large-scale residential construction near BA.

The majority of previous studies have reported that D5 is directly related to indoor concentrations due to PHCP consumption, and indirectly to outdoor concentrations. As a result, higher concentrations are observed in urban areas (within a 1 km radius from the center), where PD is higher, compared to semirural/mountainous areas, with indoor levels being higher than outdoor levels.^53–55^ However, the observation of the highest SI in SRRA_TD_ was surprising. According to recent studies, D5 is one of the significant precursors that initially contributes to SOSiA^56^ and SOA,^57,58^ then to PA. Its conversion to PA^59^ via sorption factors may also play a role in its lower SI values observed in SRRA_TD_ compared to D6–D7, and in BA_TSP_ compared to D6 and D8. There is currently no data available regarding the global production or sources of D7 and > cMSs. Although D7 is recognized as the most hydrophobic member of the group.^60^ A limited number of studies have reported data on the presence of D7–9 in the gas phase^39^ and D7 in sewage sludge,^61,62^ whereas no data exists regarding in the particle phase or D8–10 across gas/particle phases and other environmental matrices. In 20 domestic/mixed/industrial sludge samples, D7 was identified as the second most prevalent VMS (97%), while D3–4 had the lowest detection frequency (<15%).^61^ The lower presence of D7–9 in the gas phase, as compared to other compounds,^39^ further corroborates the high tendency of D7–10, similar to D6, to be found in the particle phase. The presence of D7 in both BA_TD_ and its similar sources in BA_TSP_ and MQA suggests comparable origins for D7 across these matrices. The formation of D7 through the thermal degradation of PDMS at 500 °C () suggests that it originates from industrial facilities, such as cement plants with high-temperature silicone oil usage and/or combustion licenses, and/or WWTP. The presence of D8 and D10 exclusively in BA_TSP_ and SRRA_TD_, and D9 only in SRRA_TD_, implies that these compounds likely result from complex sources such as local emissions, abiotic degradation, and SOASi. Their detection can be attributed to their predicted hydrophobicity and/or tendency to accumulate in the particle phase.

The strong local sources in SRRA were examined on-site. There was no monomer production facility, and pesticides not containing siloxane were being used. The high signals could be attributed to lubrication and maintenance activities/petroleum product consumption (fertilizer, seeds, etc. for agricultural machinery), irrigation systems, transportation, and the catalytic effect of clay minerals (from MQA) on the decomposition of silicone oils and/or the abiotic degradation of slow-release fertilizers (silicone-coated) used in large volumes (in SRRA), as well as the polymerization of linear dimethylsiloxane diols in the soil through irrigation.^63^ Previous studies have suggested that natural alkali–silica reaction (ASR) and polymerization may be natural sources of cMSs.^46^ This polymeric structure may have formed complex structures by the incorporation of one or more PTE/TEs. Previous studies have reported that organic matter (OM) forms complexes with Cu^64^ as well as other PTE/TEs such as Zn, Cr, Pb, and As, and that phthalates, aldehydes, and ketones act as ligands.^65^ The preference of ligands for metals/metalloids/cations is influenced by the chemical groups present in the environment and pH levels. The estimated high pH in SRRA may have enhanced deprotonation, allowing multiple groups to bind to the ligands.^66^ Alkali ions form complexes faster than other ions.^67^ Previous studies (e.g., atmospheric ash/oxides of Ca, Mg, and Zn by^68,69^ biomass-coal ash/size order SiO_2_, Al_2_O_3_, etc., by Al-Naiema et al.;^70^ and river sediments in Konya/size order SiO_2_, MgO, etc., by Coskun et al.^15^) confirm the dominant presence of oxides in the atmosphere. Natural polymerization may have initiated through the oxidation of NO_3_ via denitrification, resulting in the formation of (OH)_2_ compounds from Mg,^71^ Ca, and similar alkaline oxides, as well as through the reduction of amorphous silica solubility by CO_2_ via mineral carbonation. This process might have first produced silanes, followed by the formation of siloxane bridges (Si–O–Si) under alkaline conditions, leading to the generation of D4 and other cMSs. Moreover, it is suggested that complex organic compounds of D4-D6 ^48^ and other cMSs could have accumulated by sorbing onto organic carbon in aged aerosols.

### 3.4 Diagnostic Ratio

The original D4/D5 ratio (according to SI) was 2.04 in SRRA_TD_, which is twice as high as the value in BA_TSP_ (0.86). The peak area of D5 at the same concentration is half that of D4.^44^ Accordingly, the concentration-based ratios are 1 and 0.4, respectively. These findings are inconsistent with most of the literature, which generally reports a D4/D5 ratio >1 in the gas phase. However, similar results have also been reported (e.g., 0.31 and 0.87 in urban and mountainous regions, respectively,;^39^ < 1,.^72^ Despite the decrease in D5 due to SOA/SOASi, the tendency of D4 to remain in the gas phase may have contributed to the D4/D5 ratio being <1. Additionally, the extended sampling duration and precipitation-free cold periods in TDs might have led to the accumulation of D4, thereby increasing the ratio relative to TSP. Due to artifacts in the sorbents commonly used in the literature, losses of 32% in D5 during storage have been reported by,^73^ while D4 remains unaffected. This suggests that previous studies may have observed changes in both the original concentrations of cMSs and diagnostic ratios during sampling and storage. The discrepancies in D4/D5 ratios observed in the literature, such as those reported by,^39^ indicate that differences in sampling methods (e.g., sorbent/type of sampling) and weather conditions may play a significant role.

### 3.5 PA, PAEs, and 2,4-bis­(1-phenylethyl) Phenol

Anthropogenic PA is hydrophilic and serves as a precursor to SOA formed by the photoxidation of VOCs (such as naphthalene (Nap), D5, etc.), with a tendency to be present in fine particles.^59,74^ We believe that the levels of Nap in the gas phase are in the Konya atmosphere are currently high, just like in the past,^75,76^ suggesting that PA formation may occur through degradation. PAEs (DBP and DEHP) are volatile hydrophobic chemicals that are frequently detected in the atmosphere^49^ and WWTPs,^77^ especially at high levels in industrial areas.^49^ DEHP constitutes approximately 30% by weight of PVC^78^ and is the most commonly used and well-known plasticizer, accounting for 80% of those used in production.^79^ With consumption levels 10 times higher than DBP, DEHP is also the most abundant type of PAEs detected in the atmosphere.^49^ Both DEHP and DBP, which have similar peak areas according to the NIST library, were detected in BA_TD_, whereas only DEHP was identified in MQA at a signal intensity comparable to that of BA_TD_. Similar to the Beijing atmosphere (∼3.19,^49^), the DEHP/DBP ratio in BA was approximately 4.

Le et al.^49^ reported that while DBP increased in PM_0.1_ compared to PM_0.5_, DEHP decreased in an inversely proportional manner. The adsorption factors of PM and the inadequacy of low duration/volume sampling may be reasons for the failure to detect DEHP in BA_TSP_. The absence of PA–PAEs in SRRA_TD_, despite the presence of D5, was surprising. The deposition or dilution of PA–PAEs in the dense dust of SRRA and MQA, as well as local sources (e.g., lubricants) in MQA, may have contributed to their detection. The long-range transport potential of 2,4-bis­(1-phenylethyl) phenol, which is used in the industrial coating of large equipment,^80^ is low and it is only released from industrial areas, suggesting that its presence in MQA may have originated from mining machinery and equipment.

### 3.6 Alkanes

The ranking of alkanes in SRRA_TD_ according to SI is C30 > C20 > C29 > C18 > C21 > C22, with a C29/C18 ratio of 6.6. Previous studies have confirmed the presence of alkanes in biomass combustion products,^70^ in urban atmospheres (e.g., the urban atmosphere of Beijing^81^), in wash waters of heavy machinery from quarries (C9–C28,^5^), and in quarry soil (C9>^46^). However, both the size rankings and the C29/C18 ratio were only similar to those of biomass combustion. We believe that their sources may be associated not only with biodiesel consumption (with C18 being the only compound that can be linked,^82^) but also with plant-based motor/maintenance oils used in equipment such as seed-fertilizer machines, chainsaws, and sickles. This is because the sampling period (December to January) makes postharvest biomass combustion impossible, while it is suitable for wheat planting (November to December). The source of cMSs may also possibly be such equipment.

### 3.7 Elemental Contents and Sources

The concentrations, TD flux, and enrichment factors of the elements in the samples are presented in Table 3.

**Table 3:** Average Total Deposition Fluxes, Concentrations, and EFs to the Urban Background of 18 Elements (*n* = 2)

	TSP_SRRA_ (ng/m^3^)	concentrations (mg/kg)				flux (μg/m^2^d)			Turkey standard^26^ (μg/m^2^d)	Germany standard^27^ (μg/m^2^d)	EFs to the urban background (BA)	
		MQA_TD_	SRRA_TD_	SRRAT_SP_	BA_TD_	MQA	SRRA	BA			MQA_TD_	SRRA_TD_
Al	0.006	0.002	0.136	0.013	0.005	0.000	0.002	0.000			RE	RE
As	0.376	0.675	11,268	0.825	0.765	0.042	0.132	0.022		4	2.17	0.58
Ba	1130	4329	17,707	2479	6033	0.271	0.208	0.177			1.76	0.12
Cd	0.004	0.011	0.014	0.009	0.019	0.001	0.000	0.001	4	2	1.46	0.03
Co	0.007	0.018	0.234	0.016	0.045	0.001	0.003	0.001			1.00	0.20
Cr	0.058	0.327	0.118	0.127	0.977	0.020	0.001	0.029			0.82	0.00
Cu	0.126	4885	0.630	0.276	2723	0.305	0.007	0.080			4.41	0.01
Mo	0.005	0.007	0.007	0.012	0.034	0.000	0.000	0.001			0.54	0.01
Ni	0.047	0.287	0.269	0.102	0.431	0.018	0.003	0.013		15	1.63	0.02
Pb	0.049	0.412	1125	0.107	1109	0.026	0.013	0.033	250	100	0.91	0.04
Se	0.024	0.071	0.298	0.052	0.027	0.004	0.003	0.001			6.41	0.43
Zn	1161	7075	52,902	2546	7923	0.442	0.620	0.233		400	2.19	0.26
Ca	356.06	5172.88	2094.05	780.82	3441.84	323.25	24.55	101.05			3.69	0.02
K	5099	38,499	799,257	11,182	21,731	2406	9368	0.638			4.35	1.46
Mg	3320	39,745	112,855	7280	62,765	2484	1323	1843			1.56	0.07
Mn	0.130	1246	1918	0.285	2272	0.078	0.022	0.067			1.35	0.03
Na	25,842	98,183	2351,093	56,670	29,290	6135	27,557	0.860			8.23	3.18
P	32,481	17,313	517,279	71,230	31,562	1082	6063	0.927			1.35	0.65
Σ TE^a^	0.487	1425	12,525	1069	2871	0.089	0.147	0.084				
Σ PTE^b^	3081	19,247	85,951	6757	22,252	1203	1007	0.653				

a As, Pb, Cd, Cr—The top 25 substances.^83^

b As, Pb, Cd, Cr, Mn, Ni, Zn, Cu, Ba—The 100 Substances.^83^

In previous studies, it has been reported that positive matrix factorization (PMF) is inadequate for providing reliable source estimates without including OC, etc.^84^ or without additional models,^85^ and that changes in the number and composition of sources could lead to misleading results in SRRA and other areas.^86,87^ Potential sources were estimated by analyzing and correlating results and conducting on-site investigations of local sources. In TSP, Zn was found to have the highest concentration among microelements, followed by Ba and As. Kunt et al.^88^ reported that As was the second highest detected microelement in urban areas of Konya after Pb.

In all samples, Ca was the element with the highest concentration, while Na was the second highest in SRRA and MQA, and Mg was the second highest in BA. Similarly, in previous studies, Ca was found to be either the first or second highest concentration element in TDs.^36,37^ Zn was the element with the highest concentration in all samples, followed by Ba as the second highest microelement. Similarly, in urban PM_10_,^89^ and in TDs, Zn was either the first^36^ or second^90^ most concentrated microelement. Blasting operations in quarries are conducted using ammonium nitrate-based explosives, which constitute 80% of global consumption and are the most frequently and widely used, with a composition of 94% ammonium nitrate. During the initial detonation, compounds such as C_6_HN_3_O_8_Pb and Pb­(N_3_)_2_ are utilized in Cu and Al capsules; Al and Mn are employed to enhance performance.^28^ In construction machinery, elements such as Cr, Pb, As, Cd, Ni, Cu, and Zn are released due to diesel fuel/oil combustion and maintenance activities.^5,91^ Aggregates contain Ca (limestone), Si (silicate), As (salts in silicates;^92^) and serve as a source of ^40^K/K_2_O.^93^ Xu et al.^94^ reported Zn, Pb, and Cd as primary elements (PEs) sourced locally from mining; while Mn, Pb, As, and Cd were identified as elements whose atmospheric accumulation thresholds are exceeded in mining areas. In the same study, it was reported that Cu and Cr did not exhibit a normal distribution due to mining activities. In this study, Cu was found to be 8 and 2 times higher in MQA compared to SRRA_TD_ and BA_TD_, respectively; Ca was 2.5 and 1.5 times higher. Cr was detected at levels 3 times higher than those in SRRA_TD_. However, PEs were stored at the highest levels in SRRA. In MQA, Zn was the most stored microelement at 0.44 μg/m^2^d, followed by As at 0.04 μg/m^2^d (fourth), and Pb at 0.03 μg/m^2^d (fifth). Similarly, Blondet et al.^95^ reported that Zn was the second highest stored microelement in the mining area after Fe, followed by Pb in third. The presence of Pb and Zn as the first and second most stored elements, respectively, in the Beijing-SRRA_TD_, where agricultural activities are conducted,^90^ and the exceedance of atmospheric accumulation threshold values in urban areas,^95^ confirm that complex sources influence these elements. In MQA, contrary to expectations, the levels of Ba, Mn, and Al were found to be low. We believe that these levels are due to losses caused by filtration (TD). The comparison results of SRRA’s TD-TSP and MQA’s TD-PM_2.5_ also support this hypothesis (Figure 2A,B). According to the size ranking, Ba was the third most abundant microelement in MQA_TD_, whereas it ranked first in MQA_PM2.5_.^29^ It was observed that, compared to TD, the contributions of elements such as, Cd, Co, Cr, Cu, Ni, Pb, and Se decreased, while the contribution of Ba increased (Figure 2B). In SRRA, the contributions of As, Co, and Mg remained unchanged in TSP compared to TD, whereas the contributions of Cu, Cr, Al, and Ba increased. This indicates that filtering and/or adsorption factors contributed to losses of Ba. Pb and Cr, which tend to be present in fine particles,^96^ showed a surprising decrease in their percentage contributions in PM_2.5_. The abundance of Zn and Ba in MQA altered the contribution ranking of other elements; when these two elements were excluded, the contributions of Pb, Cr, Cd, and Ni in PM_2.5_ increased compared to TD. Compared to others, the increase in K, Na, Mg, P, Zn, Ba, Co, Al, Pb, As, and Se in SRRA_TD_ is attributed to agricultural activities (Zn-based fungicides; commercial fertilizers^97^ and irrigation (Na)). The increase in Cr and Mn in BA is thought to be influenced by industrial facilities and/or coal usage. Similarly, the increase in Ca, Cr, and Cu in MQA is considered to be driven by mining activities (geogenic, explosives, and/or heavy machinery). In BA, salt lakes contributed to Na levels, while in other regions, irrigation water consumption played a role. The observed increase in Na levels in the samples is associated with irrigation water use compared to BA (80 and 3.4 times higher in SRRA and MQA, respectively), which is likely to lead to an increase in SAR that reduces soil quality. In comparison to BA_TD_, the inverse relationship observed between the decrease in P and the increase in K and Na in MQA supports this hypothesis and indicates the influence of fertilizer and nonsoil sources (aggregate, underground soil, irrigation water, or well water). Previous studies^36,37^ have confirmed a strong relationship between Na in TD and CI (irrigation/sea-lake, etc.) and NO_3_ (fertilizer) (CI > 0.7 and 0.85, NO_3_ > 0.75 and 0.73), indicating high levels of CI/NO_3_ in SRRA and MQA. The high ^40^K activity in underground soil extracted through mining activities in Turkey and worldwide,^93,98^ suggests that in MQA, with its low WSA, the increase in K from aggregate sources is influenced by ^40^K rather than water-soluble K_2_O.

**Figure 2:**
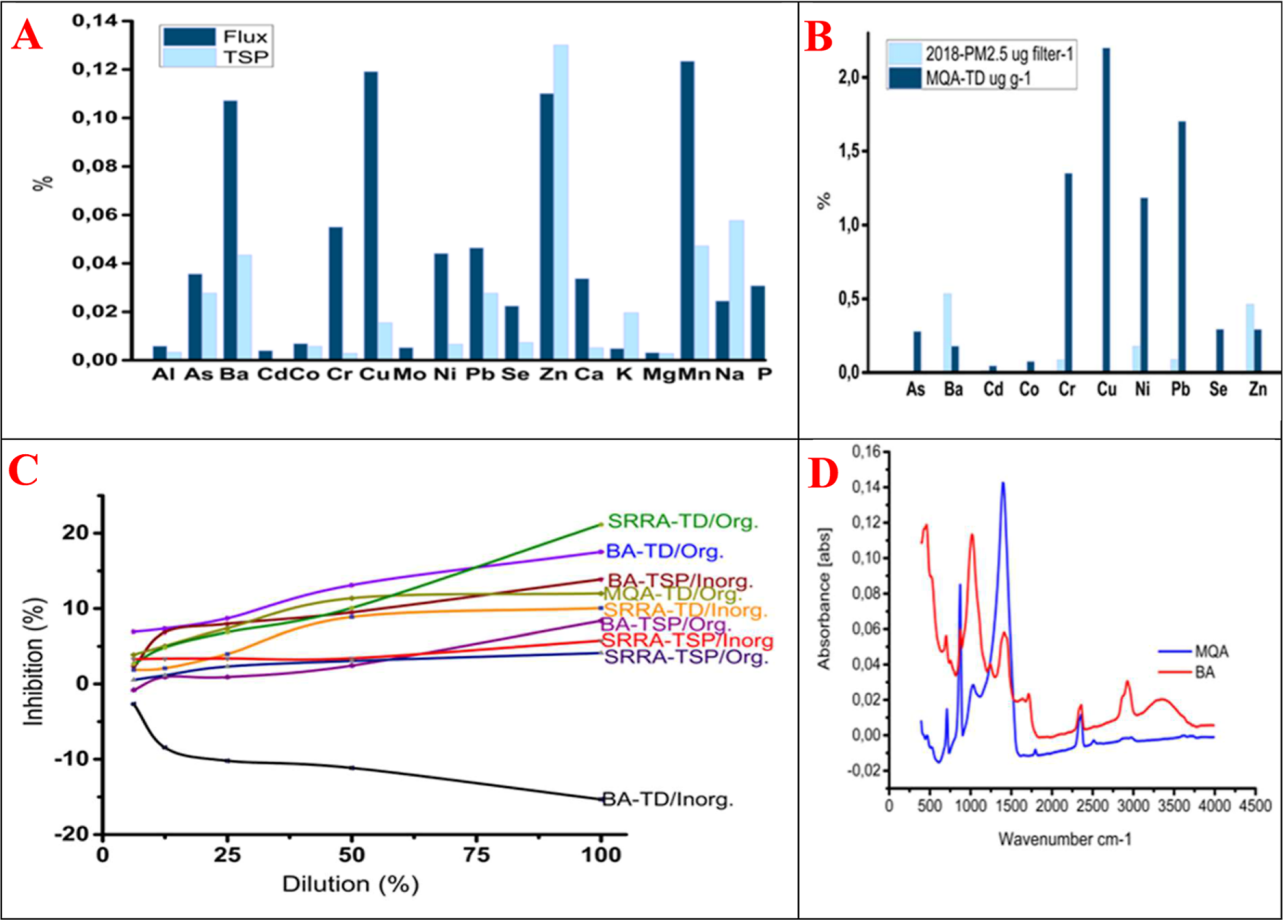
(A) Comparison of relative contributions of elements to total element concentration in SRRA-flux and SRRA_TSP_, (B) comparison of relative contributions of elements to total element concentration in MQA_TD_ and MQAP_M2.5_, (C) TDs and TSP’s organic and inorganic eluates’ EIs on L. sativum root lengths according to control, (D) FT-IR spectrum of MQA and BA-TD dusts.

EF was used to assess the degree of anthropogenic impact, specifically to determine which elements were relatively enriched compared to BA and Cu were moderately enriched, and As, Ba, Cd, Ni, Zn, Mg, and P were low. In SRRA, Na was moderately enriched, and K was low. The high level of Al in SRRA rendered the EF_SRRA_ results insufficient for the assessment when considered alone.

### 3.8 ATR–FTIR

In MQA, 8 peaks (709, 871, 1033, 1411, 1688, 1797, 2360, and 2514 cm^–1^) and in BA, 9 peaks (702, 871, 1033, 1292, 1411, 1688, 2360, 2923, and 3386 cm^–1^) were identified, along with numerous weak absorption bands (Figure 2D). For quantification purposes, the peaks at 702, 709, 871, 1033, 2360, and 3363 cm^–1^ were broad enough to allow reliable peak measurements.

This study aimed to determine the SiO_2_ in MQA. The standard interference correction method (NIOSH-7500) was applied for the most common interfering silicate minerals, kaolinite and cristobalite, using the respective ratios of 800/915 and 800/620, and the results were compared before and after correction. However, a weak peak at 871 cm^–1^ in BA and a strong symmetric CO_3_ peak in MQA overlapped with a broad-tailed peak extending from 702–709 cm^–1^ to 624 cm^–1^ (Figure 2D). Multiple species producing overlapped bending vibrations in close proximity to the 800 cm^–1^ absorption band, which is critical for reliable c-silica analysis, contributed to both tailing effects and the failure to meet the method’s validity criteria (peak height ratio of 1–1.4). These overlapping species include CaCO_3_ (875–876 cm^–1^,;^99^ NO_3_ (835 cm^–1^,^100^), amorphous silica, other silica polymorphs (800–1200 cm^–1^,^98^ and cMSs (814 cm^–1^,^101^). Despite these challenges, the presence of c-silica and silicate minerals was confirmed in both dust samples.

The characteristic stretching vibrations at 871 and 1401–1411 cm^–1^, which were approximately 1.5 and 2.8 times stronger than those in BA_TD_, respectively, indicate an abundance of CO_3_ in the MQA. The overlapping bending vibrations of Si–O, Si–O–Me, and NO_3_ contribute to the increased intensity and tailing of these peaks. The differences in the intensity of these two characteristic peaks compared to BA further support this assumption. The tailing observed at the 1401–1411 cm^–1^ band, extending toward higher wavenumbers, may be attributed to contributions from NO_3_,^100^ silicates,^102^ alkanes,^103^ and cMSs.^101^

In BA with a peak at 1033 cm^–1^ and a broad tail extending up to 900 cm^–1^, a broadening toward high wavenumbers in the intense Si–O–Si bands was observed due to metals/cations participating in the octahedral structure (Si–O–Me/cation replacing Si in Si–O–Si), which is indicative of the presence of organo complexes,^104^ and this broadening overlapped with the characteristic peak at 1135 cm^–1^ of cMSs.^101^ The asymmetric CO_3_ peak with a peak at 1401/1411 cm^–1^ has a tail extending up to 1118 cm^–1^, which overlapped with the C–H bending vibration of cMSs. In BA, a moderate intensity peak, and in MQA, a weak intensity broad asymmetric double peak at 2923 and 2852 cm^–1^ confirmed the presence of multiple methyl groups in cMSs.^105^ The presence of complex metabolites (multiple methyl groups, which bind polymeric structures to form an oligomeric structure) is also confirmed in both of these dusts. The decrease in the intensity of the C=O band, the formation of COOH^–^ and molecularly coordinated water, and the C–H and Me–OH bending vibrations, which indicate the presence of ligands in the aromatic ring, are further evidence of organo-complex formation.^106^ The presence of these complexes was confirmed by molecularly coordinated water at 1620 cm^–1^ and 1635 cm^–1^, the presence of COOH^–^ and conjugated carbonyl at 1797–1728 cm^–1^ ^107^ COO^–^ at 1797–1728 cm^–1^, O–Me–O vibration at 514–462 cm^–1^,^108^ γ­(C–H) at 709 cm^–1^ (indicating the presence of aromatic rings in the octahedral structure, i.e., the ligand in the medium), and Me/cation–OH vibration at 3600 cm^–1^. The presence of PA–PAEs was confirmed by the CH_2_ stretch for aromatic carboxylic acids (1292 cm^–1^), C–C stretch (1401 cm^–1^), OH stretch for phthalate ions (1635 cm^–1^), C=O stretch for the phthalate ester (1797 cm^–1^), broad C–H bond for PA–PAEs (2514–2923 cm^–1^), and O–H stretch for carboxylic acids observed only in BA at 3386 cm^–1^.^109^ The intensity of the broad asymmetric peak at 3386 cm^–1^, which contributes to OH stretching, also indicated that the organic species/quantity forming OH stretches were more abundant in BA compared to MQA. The presence of phenols was confirmed by the C–O stretch (1292 cm^–1^), C=C stretch (1401 cm^–1^), broad C–H bond (2960 cm^–1^), and O–H bands for phenols (3550 cm^–1^).

### 3.9 Ecotoxicity

The phytotoxicity results were generally consistent with pollutant loads, although it is difficult to directly correlate the chemical species in the PM with acute effects. The PTEs/TEs (which exhibit pH-dependent variable mobility) and/or salts that are not toxic to humans but adversely affect plant growth (e.g., Na with CI-containing salts that increase osmotic pressure and SAR) influenced the EI in both organic and inorganic phases. The results showed that L. sativum exhibited high tolerance to inorganic–organic pollution in the PM, and that root length was the most reliable parameter for determining inhibitory/bioavailability effects. Seed germination did not show any signs of being affected in any of the tested samples (inorganic/organic eluates). However, stem height did not yield comparable results.

The organic eluates in SRRA and the inorganic eluates in BA_TSP_ exhibited the highest inhibitory effects, while the inorganic eluates in BA_TD_ showed bioavailability effects (Table 1). The eluates of SRRA, characterized by organic matter for cMSs abundance and inorganic elements such as Zn, As, Pb, Na, K, Mg, and P, showed both inhibitory and bioavailability effects. Unexpected results were observed in the inorganic eluates of BA, enriched with Cu, Cr, and Pb, and MQA, enriched with Cu and Cr. The study showed that both organic and inorganic compounds in coarse dust (TD) in SRRA and inorganic compounds in fine dust (TSP) in BA, as well as organic compounds in coarse dust, contributed to a greater inhibitory effect.

In BA, the increase in P and Mg in TD and/or the bioavailability effect of Cu, Cr, and Ni may have contributed to a growth-promoting effect in the inorganic eluate. In fine dusts (BA_TSP_), similar to SRRA_TSP_, the levels of PTEs, except for Zn, may have increased, which could explain the observed EI in the inorganic eluate. We hypothesize that Fe, Mg, S, P, and K adsorbed onto coarse particles of dust transported from the fertilizer storage facility located about 5 km from BA have accumulated in BA_TD_. The fact that Mg and P levels in BA_TD_ are 2 and 3 times higher than in MQA, respectively, supports this assumption.

The EI in the organic eluates generally increased as EOM-OM increased (10% in MQA, rising to 17.52% and 21.15% in BA and SRRA-TD, respectively; Table 1). While the EOM in BA_TSP_ increased compared to SRRA_TSP_, its EI also doubled (Table 1). Compared to TDs, the EI in TSP organic eluates decreased by approximately 4 times in SRRA and 2 times in BA. Although MQA had the highest WIA (0.75 g/g), contrary to expectations, the EI in the organic eluate was about 5 times higher than that in the inorganic eluate. Similarly, the EI of the organic eluate of indoor dust for on Photobacterium was ∼1.5 times higher than that of the inorganic eluate.^19^ The organic eluate of MQA showed half the EI of SRRA_TD_ and exhibited a similar EI to BA_TD_. 2,6-bis­(1-phenylethyl) phenol was detected with low SI only in MQA. The presence of alcohol may have contributed to the completion of PA–PAEs transformation, the formation of hazardous PAEs, and the equivalent EI observed for BA_TD_ and MQA, which have 6 times higher D7 and 30,000 times higher D4 levels. Even when diluted, SRRA_TD_ exhibited the highest EI in both organic and inorganic eluates as expected. Compared to MQA, SRRA had a higher OM-EOM and was characterized by the highest GC/MS-SI, ΣTE-PTEs, and Na values. Furthermore, the contribution of OM to TS was equivalent to that of TA (Table 1), and its TA was twice that of BA.

### 3.10 Correlation between the Concentrations of Elements, Physicochemical Parameters, and Phytotoxicity Analysis

The correlation matrix of the elements in PM with each other, pH, conductivity, TDS, WIOM, WOM, and EI is presented in Supporting Information-S10. The study identified strong positive correlations between EOM and GC/MS-Σ SI (0.81), WIA and EC (0.82), EI in the organic eluate and Al, As, Ba, Co, Pb, Zn, Mg, Mn, Na, and P (>0.7), EI in the inorganic eluate and Al, As, Se, Zn, K, Na, and P (>0.7), and among Co, Pb, Mg, K, Al, Se, Ba, and P (agriculture-related elements) (>0.8). Additionally, strong negative correlations were observed between EOM and Cr, Cu, Cd, Ni, Ca, and Mo (>0.8); EI in the organic eluate and Ca and Cu (>0.95); and EI in the inorganic eluate and Cr and Ni (>0.98); WOM and EC (0.98). Although the limited number of phytotoxicity test results reduces reliability, in general, the correlations of elements with EI in the organic eluate (>0.8) were higher than those with EI in the inorganic eluate (>0.7).

Excluding As, Mo, Cd, Cu, Ni, Zn, and Ca, the strong correlations (>0.8) observed among agriculture-related elements and the productivity indicator WOM, as well as the significant correlations (>0.7) of Ni, Pb, Al, Co, Cr, and Cu with K, Zn, Na, Mn, Mo, As, and Cd, and their weaker correlations (>0.5), indicate the dominant influence of agricultural activities on the atmospheric levels of these elements. However, elements with high water solubility (e.g., As, Cd), along with MQA (Cu, Cr, and Ca), WWTPs (Cr, Cu, Ni, Pb, Zn,^110^ and industrial activities significantly influenced the correlation values. Among the elements related to agricultural activities, only As, Cu, Mn, Cd, Pb, and Mo did not show significant correlations with most of the others, yet they exhibited strikingly high correlations (>0.9) with each other. This suggests a common source, with the increased levels of As and Pb in SRRA, compared to BA and MQA, pointing to the banned insecticide PbHAsO_4_ as a potential contributor. Contrary to expectations (fertilizer) the low correlation (0.2) between P and Cd may be attributed to the levels of water-soluble Cd and/or industrial activities. The high levels of Ca, Cu, and Cr in MQA, with significant correlations between them (Ca–Cu, 0.4; Ca–Cr, 0.76; Cu–Cr, 0.8), and the direct association of Ca (lime) with certain elements related to agricultural activities (Zn, Ba, K, Mg, Na >0.8; Al, P, Co, Cr, Se > 0.7), were noteworthy. Additionally, the strong negative correlation between pH, EI in organic eluates, and EOM (>0.75) was also significant. With the exception of Cr, the significant correlations observed between Ca and Cu and elements associated with agricultural activities (Cu with Cd, Ni, Pb, Mn, Mo at 0.9, and Cr and As at 0.8) indicate that Ca and Cu are influenced by agricultural activities, whereas Cr may serve as an indicator of MQA and industrial activities. The microelement Zn, which contributed most significantly to the TDs, exhibited high correlations with agricultural activity-related elements (Al, As, Co, Se, K, Na, P at >0.95, and Mg at 0.8), similar to those observed for Ca and Cu. This demonstrates the significant mass contribution of agricultural activities (such as agricultural production/fertilizers, etc.) to the coarse dust in the Konya atmosphere. It is difficult to explain the strong relationship (0.8) between EC, which has a low correlation with pH (0.03) and negative correlations with other elements, and the insoluble species. Unlike pH, ions associated with EC maintain their stability at ambient temperature for several weeks.^111^ The significant positive correlation with WIA is observed only for Ca (0.7), indicating that different ions influence EC in each sample, but in all samples, Ca-containing species contribute to EC. The strong negative correlation between Ca and pH further confirms the dominant presence of Ca in the form of HCO_3_/CO_2_ in the final pH. In LOI analyses, the heat likely increased WIA by facilitating the volatilization of CO_2_ and the conversion of soluble bicarbonate salts into crystalline carbonate salts, as well as the degradation of complexes (above 100 °C). The conductivity of the new metabolites (organo- complexes), whose morphology and physicochemical properties have changed compared to their original states,^66^ may have decreased relative to their original forms, but the ion-dipole forces could have contributed to conductivity.^112^ The final pH (<9) and EC of both TDs, with a low positive correlation (0.226) with WIOM and a strong negative correlation (0.98) with WOM, support this assumption. The increase in organo-calcium complexes in BA and SRRA-TD may have inhibited the formation of CO_3_/HCO_3_,^65^ leading to an increase in WSA and possibly contributing to the reduced correlation of Ca with WIA. Ca bound to carboxyl groups effectively reduced the EI in the organic eluate (−0.998) by preventing the binding of PTE-TE and/or reducing their solubility (increasing pH). Cr and Ni are primarily preferred in silica complexes.^104^ Moreover, Cu, which can easily interact with both organic and inorganic substances depending on pH,^113^ can also form organic complexes in their presence. These complexes may have contributed to inverse decreases in the inorganic fraction, thereby influencing bioavailability. The strong negative correlation between EI in the organic eluate with Cu and the formation of complexes, which likely occurs after the depletion of preferred cations such as Al, and/or the degradation of some weak complexes (as indicated by the high negative correlation with EOM > 0.8), supports this hypothesis. Additionally, the high tolerance of plants^113^ may have further facilitated this process. The differences in pH, organic fraction type, and levels in the samples may have led to variations in the organo-Cu complexes and free Cu levels, resulting in bioavailability effects in certain samples (as seen with an EI in the inorganic eluate of −0.3). It is hypothesized that the contribution to the EI in the inorganic eluate of elements not preferred by the ligand, such as Al and As (in the inorganic fraction), could explain their influence on bioavailability (with EI values of 0.7 and 0.8, respectively, for Al and As in both inorganic and organic eluates). This study suggests that PTEs, such as As, Pb, and Zn, which transition to the organic phase, exhibit bioavailability effects (with EI values in the organic eluate >0.8), while some show a decrease in free concentration levels, contributing to this bioavailability effect. This explains the unexpected high EI in the organic eluate in MQA. The strong positive correlations (>0.7) of PTEs with WOM and WIOM (WOM-Co, Ni, Pb, Cr, Cu; WIOM-Cd, Ni, Pb) and the relatively low or negative correlations of Cu and some other elements with the EI in the inorganic eluate (Co, 0.64; Ni, −0.98; Pb, −0.21; Cr, −0.12; Cu, −0.28) support the tendency of certain elements to bind to OM in Urban lake sediments (OM-Cd, Pb > 0.7; OC-Cd, Cr, Cu, Mn, Pb, Zn > 0.8), with Pb showing the highest binding affinity.^114^ This hypothesis is further supported by the report of high concentrations of Zn, Mn, and Ba (1500–750 mg/kg), moderate concentrations of Pb and Cu (250–100 mg/kg), and low concentrations of Cr (<40 mg/kg) in the organic particulate matter at the Beijing air monitoring station.^115^

The significant correlation (0.74) between EI in the organic eluate and OM confirms the relationship between organic species and EI. The limited number of EOM and phytotoxicity results led to a nonsignificant (−1) relationship between EOM and EI in both organic and inorganic eluates. Therefore, the exact relationship between EOM and EI remains unknown. However, the strong correlation (0.8) between EOM and GC/MS-SI suggests that a significant portion of the species in the dichloromethane extracts was detected.

We believe that Cd, Pb, Cr, Ni, Al, Zn, Co, and Cu transition to the organic phase, supporting the polymerization of silica; furthermore, Al, Cr, Ni, Pb, and Cd may have attached to the polymeric structure. The high positive correlations observed between some elements (Co with Al and Pb > 0.8, Pb–Al, *r* = 0.7, Al–Cu, *r* = 0.6, Ni–Cr, *r* = 0.9) and the EI in the organic eluate, with the exception of Cr and Cd (>0.7), suggest the presence of complex polymeric structures, composed of multiple metal/metalloid-centered atoms.

## 4 Discussion

We found that the ATR-FTIR results provided a new perspective for evaluating physicochemical outcomes and EI, as well as for identifying metabolites. Furthermore, the results of LOIs, and EOM, when combined with FTIR, were useful for expanding the chemical domain boundaries in NTA and for understanding the composition of chemical species.

In dust with a dense matrix, ATR-FTIR proved inadequate in determining c-silica exposure, which is considered the primary cause of silicosis. Elements that cause serious diseases (such as ^40^K, As, etc.) in MQA dust, along with endocrine-disrupting chemicals commonly identified in environmental matrices were detected. Moreover, some of these elements (Ca, Cr, Cu) were at higher levels than the SRRA and BA. Na and consequently irrigation water consumption were also 3 times higher than BA. Na, Cr, Cu, and other PTEs, MQA inorganic eluate, PAEs, cMSs, and 2,6-bis­(1-phenylethyl) phenol are the suspected sources of EI in the organic eluate. Among these, 6-bis­(1-phenylethyl-phenol) and D4-D6 belong to the class of persistent substances and exhibit high biological accumulation tendencies. In fact, 6-bis­(1-phenylethyl-phenol) is toxic to humans (reproductive toxicity), while DEHP is considered a possible carcinogen. Some of these substances are also toxic to aquatic life (PA–PAEs). Regulations on PAEs and D4-D6 include restrictions on the use of D4-D6 in ″washable″ personal care products, and D4 and D5 in household cleaning products. Additionally, recommendations for restricting the use of D4–D6 in 2021 for certain industrial processes in the EU are in place. In Turkey, there are max. limits for DEHP and DBP in food products. However, there are currently no regulations regarding the use of cMSs in Turkey. We hypothesize that the original levels were higher; the large volume of inert dust diluted them, accelerated their settling, and increased the risk in the soil and aquifers. The concentrations of ^40^K and other harmful pollutants that may adversely affect human health and the ecosystem should be investigated in more detail. In this study, it was found that the TD in the SRRA increased by 17 times compared to the BA due to windward transport from the MQA. Additionally, the dust in the SRRA, enriched with elements and organic matter (OM) from agricultural activities, interacted with the dust in the MQA, which is rich in silica and elements such as Cu, Cr, Ni, and Ca that support the polymerization of silica. In the aged aerosols, formed cMSs (complex mineral structures) and new metabolites with natural polymerization. Therefore, SRRA_TD_ showed the highest EI. However, we currently lack information regarding the rate and duration of natural polymerization. Investigating the accumulation in agricultural fields was not the primary aim of this study, but the extent of contamination in SRRA is concerning. Whether it is related to MQA remains uncertain and requires further research. This highlights the necessity of combining MQA dust with other OM sources (e.g., diesel), subjecting them to aging processes, and conducting a more in-depth analysis of the resulting pollutants and their impacts.

The presence of D7–10 in atmospheric PM, whose potential risks are unknown, and the accumulation of D4 are concerning. Further comprehensive studies are needed to determine the contribution of natural ASR, particularly MQA and other sources, to this accumulation. These results should be considered a starting point for gaining a deeper understanding of the effects of MQA dust.

According to BA, MSPA and SRRA showed that there were no other significantly enriched elements besides Na and Se. This resulted from the unexpectedly high levels of elements in BA_TD_. Such an occurrence was not solely due to the influence of major pollution sources but also resulted from aerosol aging. This is because similar interactions and accumulations associated with atmospheric aerosol aging were observed in TDs. The presence of PA–PAEs and D5, suspected tracers of the POA-SOA transformation, in BA confirmed aerosol aging.

FTIR, LOI, and correlation analyses suggest that metals and metalloids may have been incorporated into the structure of cMSs and other organic compounds. The phytotoxicity results confirmed this hazardous accumulation and the dependence of EI on the chemical composition of the samples. However, unlike other TDs, the EI in atmospheric dust decreased inversely with the increase in inert dust in MQA. We hypothesize that the heavy masses of these new metabolites, with high ecological risk potential, accumulate in soil and water environments rather than in the air. Further studies investigating the accumulation in snow/water samples from MQA are needed to validate these assumptions.

## Supplementary Material



## Notes

Sampling procedure; meteorological datas; CEREVE procedure, sample preparation and physicochemical analyses; extraction and ecotoxicity analysis procedure; ecotoxicity results; FTIR, ICP-OES and GC/MS procedures; results of correlation coefficient (PDF)

## Notes

Terms & Conditions: Most electronic Supporting Information files are available without a subscription to ACS Web Editions. Such files may be downloaded by article for research use (if there is a public use license linked to the relevant article, that license may permit other uses). Permission may be obtained from ACS for other uses through requests via the Rights Link permission system: http://pubs.acs.org/page/copyright/permissions.html.
