# Supplementary material for: Comprehensive Evaluation of PM (Deposition/TSP) Pollution from Multiple Quarrying Activities
Source: ACS Omega. 2025 May 13;10(20):20353–67. doi: 10.1021/acsomega.4c11520 (PMC12120662; doi:10.1021/acsomega.4c11520)
Supplement: Supplementary file 1 [file ao4c11520_si_001.pdf]

Supporting Information

Comprehensive Evaluation of PM (Deposition/TSP) Pollution from Multiple Quarrying Activities

Gülnihal Kara[1*], Ali Çankaya[2]

[1] Konya Technical University, Department of Environmental Engineering, 42130, Konya, Turkey

[2] Konya Technical University, Institution of Graduate Education, Department of Environmental Engineering, 42130, Konya, Turkey

All correspondence should be sent to Gülnihal Kara:

Ph: +90 332 2051600

Fax: +90 332 2410635.

e-mail: gkara@ktun.edu.tr

Section1. Sampling procedure

High-density polyethylene (HDPE) bottles (1 L) that had been previously cleaned were used to collect TDs. Four collection bottles with rectangular cross-sections of 340 mm and 45 mm are attached to four collection heads (Figure S1-A). The samplers were placed on the university roof at BA (38° 1'33.20"K, 32°30'34.76"D-30 m above the ground), and on a hard flat surface (without obstacles such as walls or buildings nearby and at a collection height of 1.5 m from the ground) at SRRA (38° 5'14.82"K, 32°42'8.53"D)and MQA (38° 5'37.56"K, 32°41'15.74"D). Two simultaneous samples were taken in the dry season. Bulk samples were emptied from the collection bottles following each rainfall (to prevent any development of mo) and accumulated in (-18 °C) the laboratory. Residues that had absorbed onto the sampling head and the walls of the bottles were rinsed with ultrapure water in a clean room and accumulated in a separate clean HDPE bottle. Using pre-cleaned 37 mm GF/A filters (with a pore size of 1.6 μm (Whatman)), TSP samples were collected twice a day for 24 hours at a flow rate of 0.0538 m³/min from January 19, 2023, to January 20, 2023 (Figure S1-B). The air flow rate was checked using a rotameter (Gilmont Inst.) during each sampling. The variation in flow rate across all samples was < 3.2%. Field blanks consisted of closed-mouth HDPE bottles and parafilm-wrapped GFA filters (n=3), which were kept in the sampling area during the sampling period.

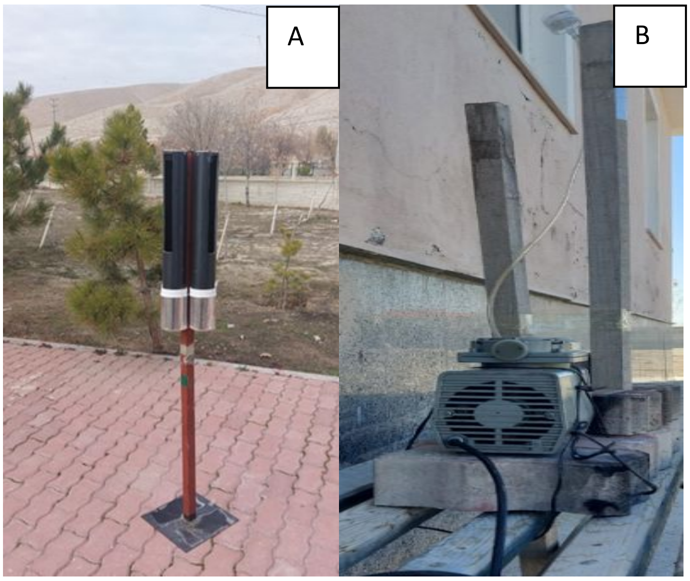

Figure S1. TD (A) and TSP (B) Samplers

Section 2. Meteorological datas

The meteorological data taken from station 37°52'07.3"N 32°28'16.7"E was used to prepare the wind diagram (air temperature-pressure, relative humidity, precipitation, wind direction-speed, etc.) and to evaluate possible transport from the MQA to the SRRA and the city center at the sampling period (Table S1).

Table S1. Meteorological datas

Gün	0	1	2	3	4	5	6	7	8	9	10	11	12	13	14	15	16	17	18	19	20	21	22	23
14	NNW 1.1	NNW 0.9	ENE 0.6	NNW 0.6	NE 0.5	SW 0.8	S 1.1	ESE 1.2	E 1.3	ENE1.5	ENE 1.4	NE 1.2	E 1.4	ESE 1.4	ESE1.5	NE 1.2	NE 1.1	NE 0.9	NNE0.5	NNW 0.6	SSW0.4	SSW 0.6	WSW0.5	W 0.3
15	WSW 0.7	WNW0.7	WNW0.5	WNW0.6	NW 0.6	N 0.5	NW 0.6	ENE 0.8	ESE1.1	SSE1.4	SE 1.4	SE 1.5	SE 1.4	SE 1.4	ESE1.2	E 1.2	ESE 1.1	SW 0.3	NW 0.5	WSW 0.5	NNW 0.7	W 0.3	SW 0.4	WSW0.3
16	WSW 0.4	WSW 0.2	NNW 0.3	N 0.3	SE 0.4	SSE 0.4	SE 0.7	SSE 1.1	ESE1.0	SE 1.1	ESE 1.1	SE 1.4	E 1.3	E 1.3	SE 1.3	ESE1.1	SSE 1.0	WNW0.5	WNW 0.5	WNW 0.4	NNW 0.8	NW 0.9	NW 0.8	WNW0.6
17	NW 0.5	WNW0.5	E 0.8	N 0.2	NE 0.7	SE 0.6	E 0.7	ESE 1.0	ESE1.0	ESE1.3	SE 1.4	SSE1.6	SE 1.7	SE 1.8	SSE1.8	SE 1.3	SW 0.9	SSW 0.7	SSW 0.9	WSW 0.9	WNW 1.2	SW 1.3	NW 1.5	NNW 1.5
18	NNW 1.7	SE 1.7	ENE 1.7	SE 1.7	SSW 1.7	E 2.8	NE 6.2	NE 6.1	ENE 6.2	E 6.4	ENE 6.3	SE 6.7	SE 6.8	S 6.7	SSW 4.6	NW 3.8	NW 4.2	NW 4.7	NW 6.5	NW 6.9	NW 6.9	N 4.4	N 3.5	NNE 3.5
19	N 3.3	S 2.8	SSE2.5	N 3.0	W 2.8	NE 3.8	ENE6.1	NNE 6.5	NE 7.0	NNE6.9	NNE 6.7	ENE6.4	ESE 6.6	ESE 6.6	SE 6.6	E 6.5	ESE 6.1	E 6.0	N 6.2	NNW 5.7	N 5.0	NW 5.8	W 5.5	W 5.4
20	NW 5.6	W 4.6	WSW3.3	WNW2.4	ESE2.3	S 4.6	S 5.6	SSE 6.5	SSE6.5	ESE6.6	SE 6.6	ESE6.6	SE 6.7	SE 6.5	ESE6.4	NE 6.7	NNE 6.4	N 3.6	NNW 2.9	N 3.2	NNW 3.2	WNW2.4	NNW 2.5	NNW 2.5
21	N 2.4	NW 2.3	NW 2.3	NW 2.2	WNW2.2	SSW 2.1	WSW 3.1	SSE 2.2	ESE1.7	E 1.7	E 1.4	ENE1.8	NE 1.8	NE 1.8	NE 2.0	NNE 4.8	N 6.6	NNW 6.5	NNW 6.7	NNW 6.8	NNW 6.0	N 6.0	NNW 3.2	NNW 3.3
22	NNW 3.0	NNE 6.1	NW 5.0	NW 5.6	NNW 4.4	SW 6.1	SE 6.1	SE 6.3	SSE6.4	E 1.4	SE 1.6	ESE1.2	NE 1.2	N 2.0	NNE 2.1	NNE 1.9	NNE 2.2	N 1.7	N 2.0	N 1.5	N 0.7	NNE 0.8	E 0.7	ENE 0.6
23	ESE1.1	E 1.3	ENE 1.3	NNE 1.4	ENE 1.1	NE 1.1	NE 1.3	SE 1.1	E 1.0	S 1.3	ESE 1.3	ESE1.6	ESE 1.6	SE 1.5	SE 1.3	SE 1.1	ESE 1.1	SE 0.8	SE 0.8	ESE0.6	ESE0.7	E 0.6	NNW 0.6	N 0.7
24	W 0.6	SW 0.4	NNE 0.5	WNW0.6	NW 0.7	WNW0.8	NW 0.8	WNW0.8	SSE1.3	SE 1.1	E 1.5	ENE1.4	ENE 1.6	E 1.7	NE 1.3	NE 0.9	ENE 1.2	ENE1.2	NE 0.5	NNW 0.3	NNW 0.2	W 0.3	WSW0.4	WSW0.3
25	WNW 0.3	WSW 0.4	NW 0.3	WNW0.5	N 0.5	ENE 0.4	SE 0.6	NNE 0.7	ESE1.0	SSE1.3	S 1.3	SSE1.3	S 1.4	ESE 1.4	SE 1.2	ESE1.1	SE 0.8	ESE0.3	W 0.3	WSW 0.3	NW 0.2	WSW0.2	NW 0.3	NNW 0.2
26	NW 0.2	SW 0.2	NW 0.2	NW 0.1	NNW 0.1	SE 0.3	ESE0.6	SE 0.7	SE 1.2	SE 1.4	SSE 1.2	ESE1.3	SE 1.5	SE 1.4	ESE1.5	ESE1.3	ESE 0.6	NNW 0.2	WSW 0.3	WNW 0.4	NNW 0.2	NW 0.2	W 0.5	W 0.3
27	WSW 0.3	WNW0.2	W 0.2	SSE0.3	NNW 0.1	NNE 0.1	NE 0.5	E 0.7	E 1.0	ESE1.2	SE 1.4	SE 1.4	SSE 1.3	WSW1.2	W 1.6	WSW 1.5	WSW1.0	WSW 1.1	WNW 1.3	S 1.1	W 1.1	SW 1.1	SSW 0.6	SW 0.6
28	SE 0.7	SE 1.0	ESE0.7	E 0.6	S 0.6	SSE 0.6	ENE0.9	ENE 1.3	E 1.1	NE 1.2	W 2.0	WSW 1.9	WSW2.3	WSW2.4	WSW1.8	W 1.7	WSW1.1	SSE0.5	W 0.6	W 0.6	W 0.5	NNW 0.8	NW 0.9	NW 0.8
29	NW 0.8	SSW 0.7	E 0.5	ENE 0.3	SSW 0.3	SSW 0.5	SE 0.5	ESE 1.1	ENE 1.2	N 1.2	W 1.7	W 2.1	W 2.3	WSW2.2	W 1.6	WNW1.4	WSW1.5	W 0.6	W 0.7	WSW 0.3	NW 0.3	WNW0.4	W 0.4	W 0.3
30	NW 0.1	N 0.2	N 0.1	WSW0.4	W 0.3	E 0.4	NE 0.8	E 0.9	ESE0.9	SE 1.5	SSE 1.8	WNW1.7	W 1.6	WNW1.5	NW 1.3	WNW1.4	W 0.5	NNW 0.2	WNW 0.2	WNW 0.2	WNW 0.6	W 0.6	WSW0.5	WSW0.5
31																								

Table S1. Continued

Gün	0	1	2	3	4	5	6	7	8	9	10	11	12	13	14	15	16	17	18	19	20	21	22	23
1	NW 0.3	S 0.4	NNW 0.3	NE 0.2	SSW 0.5	W 0.3	ENE0 .6	ENE 0.7	ESE1 .2	E 1.3	ENE 1.9	NNE 1.4	NE 1.9	NE 1.8	N 1.5	NW 1.7	NN W0.9	NNW 1.1	NNW 0.6	WSW 0.3	SW 0.6	WS W0.5	NNW 0.6	NW 0.6
2	SSW0 .5	SSW 0.8	W 0.5	N 0.3	NW 0.3	E 0.3	ESE0 .6	SE 0.8	SE 1.1	E 1.0	ENE 0.9	W 1.5	SW 1.6	SW 1.6	SSW 1.9	SW 2.0	WS W1.6	WSW 1.0	NNW 1.6	WNW 1.5	NW 1.0	NW 1.5	WS W1.1	WN W1.1
3	SW 0.9	NE 0.6	SSW 0.6	NNW 0.6	N 0.9	N 1.5	NW 0.9	NNE 1.5	NE 1.9	NE 1.8	NE 1.6	NE 1.7	NE 1.6	ENE 1.3	E 1.3	E 1.2	E 1.0	ENE0 .7	N 0.9	N 1.1	NNW 0.6	NNW 0.4	NE 0.3	N 0.2
4	NW 0.2	NW 0.3	WS W0.5	SW 0.5	SSW 0.7	SSE 0.9	S 1.4	S 1.7	SSE1 .3	NE 1.5	ENE 1.4	NE 1.4	ESE 1.4	ENE 1.3	NE 1.3	N 1.6	NW 1.1	NW 0.8	WNW 1.1	NNW 0.9	NW 1.0	WN W0.9	WS W0.8	WS W0.8
5	WNW 0.8	NW 0.6	SW 0.5	W 0.5	NW 0.5	WN W0. 5	NW 0.6	ENE 0.9	NNE 1.6	NNE2 .0	NE 2.0	NNE 2.1	NE 2.1	NNE 2.1	NNE 2.0	NNE 2.0	N 1.7	N 1.6	N 1.8	N 1.8	NNW 1.3	NNW 1.2	N 2.3	N 2.1
6	N 2.4	N 2.6	N 2.3	NNE 2.0	N 2.1	N 1.6	NNE 1.7	NNE 2.2	NNE 2.2	NNE2 .0	NNE 2.4	NNE 2.1	NNE 2.0	N 1.8	N 2.2	N 2.3	NN W1.8	N 2.2	N 2.6	N 2.7	N 2.8	N 2.4	NNE 2.2	NNE 2.1
7	N 2.5	N 2.6	NNE 1.9	NNE 1.9	NNE 2.1	NNE 1.8	NNE 2.1	N 1.4	N 1.0	NW 1.0	NN W0.8	NNE 1.7	NE 2.1	NE 1.9	NNE 2.0	NNE 1.6	N 1.5	WN W0.8	NW 0.9	NNW 0.7	SW 0.7	ENE 0.8	N 0.5	N 0.5
8	SSE0. 7	S 0.7	SSE0 .5	S 0.4	SW 0.4	S 1.0	SSW 0.9	SW 0.7	SSW 1.1	SSE1 .3	SSE 1.6	SSE1 .4	SE 1.0	NNE 1.3	N 1.3	SW 1.0	SSW 0.9	NW 0.4	N 0.3	N 0.3	NW 0.3	WN W0.3	SSE0 .6	SE 0.5
9	S 0.6	S 0.5	SW 0.5	S 0.5	SE 0.6	S 0.7	SSW 1.0	SSE 1.3	SE 1.2	ESE1 .3	ESE 1.0	ENE1 .3	ESE 1.1	ESE 0.9	E 0.8	NNW 1.1	N 1.8	NNW 1.1	N 0.5	SSE0 .7	SSW0 .6	S 0.5	W 0.3	WS W0.3
10	N 0.2	SSW 0.2	SSE0 .4	N 0.2	NNW 0.2	E 0.2	NNW 0.3	S 0.9	SSE1 .1	ESE1 .3	SSE 1.0	E 1.0	E 1.2	NE 1.3	NE 1.2	NNE 1.4	NNE 1.1	N 1.0	NNW 0.5	NNW 0.3	SW 0.4	SW 0.3	NW 0.3	NW 0.1
11	NNW 0.1	W 0.1	NNW 0.1	NW 0.1	WN W0.2	NW 0.1	WN W0.3	ENE 0.6	SE 1.0	ESE1 .2	SE 1.1	SE 1.1	ESE 1.2	SE 1.1	ESE1 .2	E 1.0	E 0.4	WN W0.3	W 0.2	WNW 0.3	NW 0.3	NNW 0.2	NW 0.4	WN W0.4
12	W 0.2	W 0.2	WN W0.2	NW 0.1	NNE 0.1	N 0.1	SE 0.5	ENE 0.6	ESE0 .6	SSE0 .9	S 1.1	ENE1 .0	NE 1.0	ENE 1.1	NE 0.9	N 1.1	N 0.9	NNW 0.9	NNW 1.1	NNW 0.9	NNW 1.0	NNW 0.7	NNW 0.5	NW 0.6
13	NNW 0.4	S 0.2	WS W0.3	SSW 0.3	N 0.1	NNE 0.1	S 0.5	ENE 1.0	NE 1.5	N 1.3	NNE 1.2	NE 1.8	NE 1.8	NE 1.6	NNE 1.5	NE 1.3	NE 0.8	N 0.6	NNW 0.5	NNW 0.1	SW 0.2	NW 0.1	WN W0.3	NW 0.1
14	W 0.3	WSW 0.1	NW 0.2	NNE 0.2	NNW 0.1	WS W0. 4	W 0.3	SW 0.5	ESE0 .9	ESE1 .0	SSE 1.0	SE 1.4	ESE 1.2	ESE 0.8	ENE 0.6	N 0.4	NN W1.0	N 2.0	N 1.6	N 1.8	N 1.6	N 1.7	NNE 1.0	N 1.1
15	N 0.6	W 0.2	WS W0.3	C 0.0	NNW 0.1	WN W0. 2	WSW 0.3	S 0.6	ESE0 .8	SE 1.1	SSE 1.1	SSE1 .0	SE 1.3	SSE 1.0	SE 1.0	E 1.0	ENE 0.5	E 0.9	S 1.3	SSW 1.0	S 0.5	NNW 0.4	NE 0.4	ENE 0.5
16	NNW 0.4	ENE0 .7	ESE0 .6	NNE 0.4	NNE 0.4	NNE 0.3	E 0.6	S 1.1	SSE0 .9	SSE0 .8	ESE 1.0	ESE1 .6	ESE 1.7	ESE 1.4	SE 1.0	NNE 0.6	NNE 0.8	NE 1.0	NNE0 .9	N 1.0	N 0.8	NNE 0.7	NNE 1.3	NNE 1.3
17	N 1.1	NNE 0.8	N 0.7	N 0.6	NNE 0.9	N 1.6	N 1.8	N 1.9	N 2.3	N 1.9	NNE 1.8	NNE 2.0	N 2.4	NNE 2.3	NNE 2.0	N 2.0	N 2.0	N 2.3	N 2.7	N 2.6	NNE3 .0	N 2.4	N 2.0	N 1.8
18	N 1.4	NNE 1.3	N 2.0	N 2.3	N 2.3	N 1.8	N 2.4	N 2.6	NNE 2.1	N 2.6	NN W2.8	N 2.5	N 2.5	N 2.1	N 1.7	NNW 1.5	NW 0.9	NW 0.8	NW 0.7	WNW 0.7	WNW 0.9	NNW 0.9	N 0.7	N 0.6
19	SSE0. 7	S 0.6	SSE0 .7	NNW 0.4	SE 0.5	SSW 0.6	SSW 0.6	S 1.0	SSE1 .3	SSE1 .0	ESE 1.1	SSE0 .9	SE 1.4	SE 1.3	SE 1.1	S 1.2	SE 0.4	ENE0 .2	SSE0 .4	W 0.3	S 0.4	N 0.1	N 0.3	N 0.1
20	NNW 0.2	E 0.5	NNE 0.2	N 0.1	N 0.1	N 0.2	ENE0 .5	ESE 0.8	SE 1.1	SSE1 .4	ENE 1.8	NE 2.0	NE 1.3	NE 1.3	NE 1.5	NNE 1.3	NE 0.7	N 0.9	N 0.7	WSW 0.4	W 0.4	WN W0.5	N 0.5	N 0.5
21	NNW 0.8	NW 0.6	NW 0.6	N 1.1	NNE 1.4	NNE 2.0	N 1.9	NNE 2.1	NNE 2.3	NE 2.4	NNE 2.8	NNE 2.6	NNE 2.3	N 2.2	N 2.2	N 2.3	N 1.5	NNW 1.7	N 1.9	NNW 1.6	NNW 1.6	NNW 1.5	N 1.5	N 1.6
22	N 1.2	NNW 1.4	N 1.3	NNW 1.1																				
23																								
24						SE 1.1	SSE1 .2	SE 0.9	SSE1 .1	S 1.0	S 1.0	SSW 0.9	ESE 0.9	NNE 1.1	NNE 1.4	NNE 1.3	NNE 0.7	NNE 0.3	NNW 0.5	NNW 0.2	SW 0.4	WS W0.2	W 0.3	W 0.3
25	W 0.3	WSW 0.3	NNW 0.1	NNW 0.1	NNW 0.2	NW 0.2	SSE0 .4	SE 0.7	SSE0 .7	E 1.0	NNE 1.2	E 1.1	E 1.2	ESE 1.1	E 1.1	E 0.7	NN W0.3	NW 0.2	WNW 0.2	WSW 0.2	WSW 0.3	WS W0.3	WS W0.4	WS W0.4
26	WSW 0.2	W 0.2	NW 0.3	NNE 0.2	NE 0.3	NN W0. 3	N 0.7	ESE 0.8	ESE0 .9	SE 1.1	ESE 0.9	E 1.1	ENE 1.4	ENE 1.3	E 1.3	E 1.0	ENE 0.3	NNE 0.5	NW 0.4	SW 0.4	NW 0.3	NNW 0.3	NW 0.5	NNW 0.3
27	W 0.4	WSW 0.2	W 0.3	NNW 0.1	WN W0.2	NW 0.2	ENE0 .3	ESE 0.8	SE 1.0	SE 1.1	SSE 1.0	N 0.9	NNE 1.3	NNE 1.5	NNE 1.6	NNE 1.4	NNE 0.9	N 0.7	NNW 0.6	SW 0.3	WNW 0.3	WN W0.4	W 0.4	W 0.4
28	NW 0.5	W 0.4	NNE 0.2	NNW 0.4	NNW 0.7	NN W0. 8	N 1.3	N 1.5	NNE 1.8	NE 1.9	NE 2.0	NNE 2.0	NNE 2.0	NNE 2.2	NNE 2.1	NNE 1.9	NNE 1.4	NNW 0.9	NNW 0.9	N 1.3	N 1.2	N 1.2	NNW 1.4	NNW 1.3
29	NNW 1.3	N 1.3	N 1.0	N 1.5	N 1.4	N 1.0	N 0.8	NNE 2.1	NNE 2.0	NE 1.6	NE 1.5	NE 1.8	NE 1.8	NE 2.1	NE 1.7	NE 1.6	NNE 1.2	N 0.9	NNW 0.8	NNW 0.4	WNW 0.4	NW 0.2	NW 0.4	NW 0.4
30	WNW 0.4	W 0.3	C 0.0	NNW 0.1	NW 0.2	NE 0.5	NE 0.3	NNE 0.7	NNE 1.3	NNE1 .2	NE 1.0	SE 1.1	ESE 1.1	SE 0.8	E 1.0	SE 0.6	N 0.1	C 0.0	C 0.0	NW 0.1	W 0.4	NNW 0.2	N 0.3	N 0.1
31	N 0.1	WSW 0.2	NW 0.3	S 0.4	NE 0.2	N 0.2	N 0.5	N 0.7	ESE0 .6	SE 0.9	E 1.0	SE 1.0	E 0.9	NE 0.9	NE 0.7	NNE 0.4	N 0.3	C 0.0	NNW 0.1	NW 0.3	S 0.2	NNE 0.2	NNW 0.4	NW 0.2

Table S1. Continued

Gü	0	1	2	3	4	5	6	7	8	9	10	11	12	13	14	15	16	17	18	19	20	21	22	23
1	NNW 0.3	WSW 0.2	N 0.1	WN W0.3	N 0.1	SSE0 .4	NE 0.4	SE 0.7	ESE1 .1	ENE1 .3	ENE 1.5	NE 1.6	NE 1.9	ENE 2.0	NE 1.6	NE 1.4	NE 1.0	N 0.8	WNW 0.4	WNW 0.3	SE 0.3	NNW 0.2	WS W0.4	WS W0.4
2	W 0.5	WSW 0.3	NNW 0.4	NNW 0.1	NNW 0.2	NW 0.3	SE 0.5	SE 1.0	SE 0.8	E 1.1	ESE 1.1	N 1.0	NE 1.1	ENE 1.2	NE 1.2	ENE1 .0	ENE 0.8	NNE 0.4	NW 0.4	W 0.4	WSW 0.6	WN W0.4	W 0.3	NW 0.1
3	SW 0.4	SW 0.4	NNW 0.2	NNE 0.1	NNE 0.1	SSE0 .4	SSE0 .4	SSE0 .7	SE 1.1	ENE1 .1	E 1.0	ESE1 .0	ENE 1.0	E 1.0	S 0.7	SW 0.5	NN W0.4	W 0.4	NW 0.3	WNW 0.2	WNW 0.3	NNW 0.3	N 0.2	C 0.0
4	NW 0.2	SSW 0.2	WN W0.2	SW 0.4	SW 0.2	N 0.1	ESE0 .3	SSE0 .7	SSE0 .8	ESE1 .1	ESE 1.1	E 1.1	ENE 1.0	SSE 0.8	SSE0 .7	SW 0.3	NW 0.2	NW 0.2	W 0.3	C 0.0	NNW 0.1	SE 0.2	N 0.3	N 0.1
5	NNE0 .2	SSE0 .3	SSE0 .5	NNE 0.1	N 0.1	N 0.1	E 0.5	E 0.7	ESE0 .8	E 0.9	ENE 1.1	E 1.0	SE 1.3	ESE 1.2	ESE1 .1	E 0.8	NNE 0.2	NW 0.3	WNW 0.2	WNW 0.2	WNW 0.2	NW 0.1	S 0.3	S 0.2
6	NNW 0.2	SW 0.1	N 0.1	E 0.2	WS W0.2	NNW 0.1	NNE 0.4	ESE0 .7	ESE0 .7	SSE0 .8	SE 1.2	SE 1.4	SE 1.5	ESE 1.3	ENE 0.8	NNE 0.7	NE 0.4	NNE 0.4	ENE0 .5	S 0.5	NW 0.5	ESE0 .5	S 1.5	S 1.6
7	W 0.6	ENE0 .6	NW 0.3	NNW 0.1	SW 0.4	SSW 0.6	S 0.9	SSE0 .6	SSE1 .0	SE 1.1	ESE 0.9	SE 1.0	SE 0.9	N 0.7	NE 0.5	N 0.5	N 0.5	NW 0.4	NNW 0.8	NNW 0.7	NNW 0.8	NNW 0.4	W 0.4	NW 0.3
8	W 0.7	WN W0.9	WN W0.5	NNW 0.8	N 0.9	NW 0.6	NW 0.5	N 1.6	N 1.9	NNE2 .0	N 2.1	NNE 2.2	NNE 2.0	N 1.6	NNE 1.6	NNE 1.8	NNE 2.0	N 1.8	N 1.0	N 0.9	N 0.8	N 0.7	NNE 1.0	NNE 0.9
9	NNE1 .0	N 1.0	N 1.0	N 0.8	N 0.8	N 1.2	NNE 1.8	NNE 1.5	N 1.7	NNE1 .9	N 2.1	N 1.9	NNE 1.3	NNE 1.4	N 1.8	N 1.9	NN W1.3	NNW 1.1	NNW 0.5	NNW 1.0	NNW 0.6	WN W0.5	WN W0.6	NW 0.6
10	W 0.5	WN W0.3	NW 0.3	W 0.5	W 0.4	N 0.2	N 0.2	SE 0.6	E 1.2	NNE1 .9	NE 2.0	NE 1.6	NNE 1.7	NE 1.7	NNE 1.5	NNE 1.3	NE 0.8	N 0.4	NW 0.3	WSW 0.3	WNW 0.3	WN W0.5	W 0.5	W 0.4
11	W 0.3	SW 0.2	C 0.0	W 0.2	NW 0.2	N 0.1	S 0.4	SSE0 .7	SE 0.8	SE 1.0	NW 0.9	ENE1 .0	E 1.3	ESE 1.2	E 1.3	ENE0 .9	NE 0.3	NNW 0.2	NNW 0.1	WSW 0.3	W 0.3	NNW 0.1	W 0.6	W 0.6
12	WNW 0.3	WN W0.4	SE 0.3	NNW 0.2	NW 0.1	NNW 0.1	NNE 0.2	NNE 0.7	ENE 0.8	ESE1 .0	ENE 1.0	E 1.1	E 1.3	E 1.2	ENE 1.0	ENE0 .8	NNE 0.2	N 0.6	WNW 0.3	WNW 0.3	W 0.5	WS W0.4	NW 0.5	WN W0.6
13	N 0.4	NW 0.3	NNW 0.2	NNW 0.3	NNW 0.3	WN W0.3	SSE0 .5	SE 0.7	NE 1.0	NE 1.3	NE 1.6	ENE1 .8	NE 1.6	NNE 1.5	NNE 1.2	NNE 0.8	N 0.4	NNE 0.3	W 0.3	NW 0.2	W 0.4	W 0.6	WN W0.5	WN W0.5
14	W 0.4	SW 0.5	WS W0.4	W 0.4	WN W0.3	NNW 0.2	W 0.3	N 0.5	SE 1.0	ENE1 .3	ENE 1.5	NE 1.7	NE 1.7	NE 1.8	NE 1.5	ENE1 .2	ENE 0.7	SW 0.3	WNW 0.2	WNW 0.2	WSW 0.4	W 0.5	WN W0.4	WN W0.3
15	W 0.5	W 0.2	WN W0.4	NW 0.2	NW 0.2	NNW 0.1	SE 0.3	ESE0 .6	E 0.8	ESE1 .0	ESE 1.1	E 1.2	E 1.2	SE 1.2	SE 1.3	SE 0.6	N 0.1	NNW 0.2	WNW 0.2	W 0.2	NW 0.2	NNW 0.3	NW 0.4	WN W0.3
16	NW 0.2	SE 0.4	S 0.3	NW 0.3	W 0.2	N 0.1	NNE 0.6	ENE 0.9	ESE0 .8	ESE1 .0	ESE 1.3	SE 1.4	SSE 1.3	SE 1.3	NW 1.1	NW 0.6	W 0.3	WN W0.3	SSE0 .4	SW 0.6	E 0.4	SW 0.8	SSW 0.5	S 0.5
17	SW 0.6	NNE 0.4	ESE0 .7	SSE0 .4	E 0.6	ESE0 .6	NNE 0.4	SE 0.7	ESE0 .8	ESE1 .1	E 1.2	ESE1 .2	SW 1.8	WS W1.6	WS W1.7	WSW 1.2	SSW 0.7	N 1.5	S 1.6	SSW 1.3	NNE0 .5	S 0.8	S 1.1	SSE1 .0
18	S 1.2	S 1.0	SE 0.7	NE 0.4	N 0.2	E 0.5	NE 0.5	SSW 0.7	W 0.5	SE 0.6	S 0.6	SSW 1.6	SSW 2.3	S 2.5	S 2.2	SSE1 .6	S 2.0	S 2.0	S 1.8	SSW 1.8	SE 1.1	SSE2 .2	WS W0.6	SW 0.8
19	SSW0 .4	S 1.7	S 1.4	SW 0.8	SE 0.6	SSE0 .6	ESE0 .5	NNE 0.8	SSE1 .6	SSE2 .0	SSE 1.8	SSW 1.2	S 1.4	SSE 1.3	S 0.9	SSW 0.9	WS W0.4	W 0.3	SW 0.4	S 0.5	WSW 0.4	SE 0.5	SSW 0.7	SSW 0.6
20	SW 0.6	ESE0 .5	ENE 0.8	ESE0 .7	ESE0 .6	ESE0 .6	NE 0.5	E 0.7	ENE 0.7	SE 1.0	SE 1.1	SE 1.4	SE 1.6	SE 1.6	ESE0 .9	NNW 0.9	NN W0.5	WN W0.6	NW 0.4	SSW 0.6	WSW 0.3	SSW 0.4	S 0.5	S 0.5
21	S 0.4	S 0.5	ESE0 .3	W 0.6	NNE 0.5	ENE0 .5	NE 0.4	S 0.6	SE 0.8	ENE0 .8	NE 0.5	S 1.3	SSE 1.6	SSE 1.2	SE 0.3	N 0.4	S 0.5	ESE0 .7	S 2.2	SSE1 .4	SSE0. 8	SSW 0.4	NE 0.5	NE 0.3
22	SSE0. 5	S 0.4	SSW 1.1	SW 0.6	W 0.6	SE 0.9	ENE0 .6	WS W1.4	SW 1.3	S 1.7	S 1.8	SSW 1.9	WN W1.4	WS W1.2	WS W0.9	NW 0.6	NW 0.5	WN W0.3	WSW 0.5	ESE0 .5	WSW 0.4	SW 0.4	SSW 0.4	SSW 0.4
23	S 0.5	SSW 0.2	N 0.1	SSE0 .4	SSW 0.4	SW 0.4	SW 0.5	SE 0.9	SSE1 .5	SSE2 .2	S 2.9	SSE2 .9	SSE 2.9	S 2.5	SSE3 .0	S 1.8	S 1.2	SSE1 .3	SSE1 .1	SSE1 .4	E 0.5	ESE0 .8	ENE 0.6	ENE 0.6
24	NNW 0.4	SE 1.0	S 1.0	S 0.7	SSW 0.6	SSE1 .1	SSW 0.9	SSW 0.8	SW 0.6	SSW 1.0	SW 0.7	S 1.2	N 1.7	SSW 1.4	SW 1.6	SSW 0.9	SW 0.9	SSW 0.8	SW 0.5	SSW 0.6	S 0.5	WN W0.5	W 0.8	W 0.8
25	W 0.5	NW 0.7	N 0.7	N 0.3	NNW 0.1	S 0.5	SE 0.5	E 0.8	ESE0 .8	S 0.9	NN W1.3	N 1.1	NN W1.1	NW 1.1	N 1.1	N 2.1	NNE 2.2	N 1.9	NNW 2.0	NNW 2.0	NNW 1.8	NNW 1.4	NNW 1.4	NNW 1.6
26	NW 1.2	NNW 1.5	NNW 1.3	NW 0.8	NNE 1.4	NW 1.2	NNW 1.3	NNE 1.2	NE 1.3	ENE1 .2	NE 1.3	SSE1 .2	NW 1.0	N 1.3	N 1.2	NNE 1.6	N 0.7	NNE 0.6	NW 0.5	NW 0.4	NW 0.2	NW 0.3	W 0.5	W 0.5
27	WNW 0.3	WSW 0.2	W 0.3	WN W0.2	WN W0.3	WN W0.3	WN W0.3	WN W0.4	NNW 0.6	SW 0.7	SSE 1.1	SE 1.1	S 1.1	E 1.1	SE 1.3	N 1.4	SSW 0.6	WN W0.2	C 0.0	NW 0.2	NNW 0.2	WN W0.1	W 0.5	W 0.4
28	WNW 0.3	NW 0.1	WN W0.3	SE 0.3	SE 0.7	SSE0 .3	SSW 0.3	SW 0.6	SSE0 .9	SE 1.3	SE 1.2	SSE1 .5	SE 1.1	SE 1.2	ESE0 .8	SSE0 .8	S 1.0	SSW 0.6	SSW 0.9	SSW 0.6	SE 0.5	SW 0.6	S 0.5	SE 0.5
29	S 0.5	SE 0.4	S 0.4	NNW 0.2	E 0.4	N 0.2	SSW 0.4	S 0.6	SE 0.7	ENE0 .7	NNE 0.8	SSE1 .0	SE 1.0	E 1.1	SE 1.1	ESE0 .6	WN W0.3	NNW 0.1	NW 0.3	NNW 0.2	NW 0.2	NW 0.1	SW 0.4	W 0.2
30	N 0.2	N 0.3	W 0.2	WS W0.2	S 0.3	NNW 0.1	NE 0.3	NNE 0.9	NNE 1.0	NE 1.0	E 1.0	SSE0 .9	SSE 0.7	ESE 0.9	E 1.0	E 0.6	NW 0.2	WSW 0.3	WNW 0.2	N 0.1	NNW 0.1	N 0.1	N 0.3	N 0.1
31																								

Table S1. Continued

Gü	0	1	2	3	4	5	6	7	8	9	10	11	12	13	14	15	16	17	18	19	20	21	22	23
1	W 0.2	SSE0 .3	S 0.3	ESE0 .4	E 0.6	WSW 0.5	WN W0.2	E 0.4	SSE0 .6	SE 0.8	SE 1.3	SE 1.6	SSE 1.3	SE 1.4	SE 1.1	WN W0.6	WS W0.9	E 0.4	SSW 0.5	SE 0.5	WNW 0.4	E 0.4	NNE 0.8	NNE 0.7
2	W 0.7	N 0.6	N 0.3	SW 0.5	E 0.5	SSE0 .4	SW 0.4	S 0.6	SW 0.8	S 0.9	SE 1.4	S 1.4	S 1.2	SSW 1.2	SSW 0.9	NW 0.7	N 0.5	NNW 0.1	WNW 0.5	WNW 0.4	WNW 0.4	SSE0 .5	WN W0.6	WN W0.7
3	NW 0.2	WSW 0.3	W 0.5	NNW 0.2	NW 0.5	NW 0.4	WN W0.4	SSE0 .7	SSW 0.8	SE 1.0	ENE 1.0	E 1.4	E 1.3	E 1.1	E 1.2	ENE1 .3	NNE 0.7	N 0.4	WNW 0.3	WNW 0.4	WNW 0.5	WN W0.6	W 0.8	W 0.8
4	W 0.7	W 0.5	WN W0.5	WN W0.4	W 0.8	W 0.8	W 0.5	NNW 0.4	NNE 0.9	NNE1 .3	NE 1.3	ENE1 .4	ENE 1.3	NE 1.3	ENE 1.3	NE 1.4	N 0.7	NNW 0.3	NNW 0.1	NNW 0.2	WNW 0.6	NW 0.4	WN W0.5	WN W0.4
5	W 0.3	SW 0.1	NW 0.3	NW 0.3	NW 0.3	WN W0.4	W 0.5	W 0.4	ENE 0.9	NE 1.5	ENE 1.6	NE 1.5	NE 1.2	NE 1.2	NE 1.3	NNE 1.1	N 1.0	N 1.0	N 1.0	N 0.9	N 1.0	N 0.9	S 0.5	SSW 0.4
6	SE 0.5	ESE0 .3	WN W0.4	SSW 0.4	SSE0 .6	SSE0 .5	S 0.4	SE 0.9	ESE0 .9	E 0.9	SSE 1.1	E 1.2	ESE 1.4	ESE 1.0	ESE1 .1	ESE0 .9	E 0.9	ENE0 .6	N 0.1	SSW 0.2	WSW 0.3	W 0.2	W 0.3	WN W0.2
7	W 0.4	WN W0.2	NNW 0.2	SSE0 .5	SSW 0.3	N 0.1	NE 0.3	ESE0 .6	ENE 0.8	SE 1.0	SE 1.2	S 1.0	SE 0.9	SE 1.0	SSE0 .6	NW 0.4	N 0.3	NNE 0.3	NE 0.2	SE 0.4	SE 0.3	NNE 0.2	SSW 0.5	SSE0 .4
8	SW 0.5	SSW 0.3	WS W0.2	NE 0.2	NW 0.3	E 0.3	W 0.5	S 0.4	WS W0.5	SE 1.2	SE 1.4	SE 1.2	SE 1.4	SE 1.2	WN W0.7	NW 0.6	WN W0.4	NNW 0.3	WNW 0.6	S 0.6	SSE0. 7	E 0.3	SW 0.6	WS W0.6
9	NW 0.3	WN W0.2	ESE0 .4	E 0.6	N 0.5	NE 0.4	NNE 0.5	NE 0.7	ESE0 .9	SE 0.9	ESE 1.1	ESE1 .4	E 1.2	E 0.9	ESE0 .7	ENE0 .4	N 0.3	W 0.5	W 0.2	W 0.2	W 0.3	NNW 0.2	N 0.4	N 0.1
10	NNE0 .2	SSW 0.1	ESE0 .3	SSE0 .5	NNE 0.2	E 0.3	SE 0.6	E 0.5	SE 0.8	ESE0 .7	SE 1.0	SE 0.9	SSE 1.0	SSE 0.9	S 0.8	SW 0.7	SE 0.5	SSE0 .5	NNE0 .4	SSE0 .5	S 0.4	E 0.3	SSE0 .5	SSE0 .5
11	S 0.7	SW 0.4	ESE0 .5	SE 0.5	ESE0 .4	NE 0.5	E 0.6	E 0.6	NNE 0.8	NNE0 .6	N 0.8	NNW 0.5	N 0.5	S 0.6	SSW 0.9	S 1.5	S 1.2	S 1.4	S 1.2	SSE1 .7	S 2.1	S 2.3	S 1.6	N 1.6
12	S 2.1	S 1.3	ESE0 .5	SSE1 .5	S 1.3	S 1.2	S 2.0	SSE1 .8	SSE2 .0	SSE1 .5	S 1.6	SSE1 .8	SSE 1.7	SE 2.0	ESE1 .3	SSE0 .8	SSW 0.6	SSW 0.7	SW 0.5	S 0.6	S 0.8	S 0.9	S 0.7	S 0.7
13	SE 0.9	NW 0.5	SW 0.6	ESE0 .7	W 0.6	SSE0 .7	SW 0.7	E 0.8	SSE0 .8	SSE0 .9	SSE 1.7	SSE2 .1	S 1.7	SW 1.2	SSW 0.9	S 0.9	SSE 1.0	SSE0 .9	SSE1 .6	S 2.6	S 1.4	S 1.4	S 1.0	S 1.1
14	SSE0. 7	S 0.5	NW 0.3	NNE 0.4	NNW 0.3	NW 0.4	N 0.4	WN W0.3	ESE0 .5	ESE0 .7	SE 0.7	S 0.9	WS W0.7	NW 0.6	WN W0.5	W 0.6	SE 0.6	N 0.5	NNW 0.5	N 0.4	N 0.3	SSW 0.3	SSW 0.5	S 0.5
15	NW 0.2	W 0.2	NNE 0.5	N 0.4	NNE 0.5	NNW 0.4	WSW 0.3	SSW 0.4	SE 1.0	S 1.3	S 1.3	S 1.5	SW 1.2	SW 1.2	WS W0.8	WSW 1.0	NW 0.6	WSW 0.7	SSW 0.5	S 0.6	NE 0.4	SSW 0.4	SSE0 .4	S 0.4
16	S 0.5	ESE0 .5	SW 0.4	NNE 0.4	NE 0.4	NE 0.3	SSW 0.4	SW 0.5	SE 0.7	E 1.0	ENE 1.2	ESE1 .0	ESE 1.0	E 0.8	NE 0.8	NE 0.6	NN W0.6	NNW 0.5	NW 0.2	W 0.3	WNW 0.3	W 0.4	NW 0.6	NW 0.3
17	WNW 0.4	SSW 0.3	NNW 0.2	NE 0.1	SW 0.3	N 0.1	NNE 0.2	ESE0 .7	E 0.8	E 1.0	E 1.1	E 1.2	ESE 1.0	E 0.9	E 0.8	NE 0.5	N 0.4	N 0.3	SE 0.2	WNW 0.4	SSW0 .3	NNW 0.3	W 0.4	W 0.3
18	N 0.3	ENE0 .3	SSE0 .4	SSE0 .4	S 0.2	NNE 0.1	NE 0.2	SE 0.7	ESE0 .9	E 1.0	ENE 1.0	E 1.0	E 1.1	E 1.0	E 0.6	S 0.3	WN W0.2	WSW 0.4	WSW 0.2	SW 0.3	WNW 0.2	NNW 0.1	NNE 0.4	N 0.1
19	N 0.2	N 0.1	NE 0.2	NNE 0.2	ESE0 .6	E 0.6	SSW 0.6	SSW 0.9	WS W0.8	SW 0.8	S 1.2	SSW 1.5	SSW 1.3	S 1.5	SSW 1.2	SSW 0.9	SW 0.8	SW 0.9	SSE1 .2	WSW 0.6	NNE0 .4	W 0.6	N 2.0	N 1.8
20	NNE2 .5	NNE 2.4	NNE 2.8	NNE 2.9	NNE 2.6	NNE 2.8	NNE 2.5	NNE 2.8	NNE 3.1	NNE2 .7	N 2.7	N 2.4	N 2.3	NNE 2.5	NNE 2.2	NNE 2.0	NNE 2.9	NNE 2.7	NNE2 .5	NNE2 .6	NNE2 .1	N 1.9	NNE 1.8	NNE 1.8
21	NNE2 .0	N 1.6	N 1.8	N 1.7	NNE 1.7	NNE 1.7	N 1.7	NNE 1.9	N 1.8	NNE1 .6	NE 1.4	NE 1.4	ENE 1.6	NE 1.5	NE 1.2	ENE1 .0	ENE 1.0	E 0.8	SE 1.0	S 1.2	WSW 0.8	S 1.0	SSW 0.9	SSW 1.0
22	SW 0.8	SSW 1.1	SW 0.9	SSW 0.8	SSW 1.1	SSW 1.0	WSW 0.9	S 1.4	SSW 1.2	SSW 0.9	SSW 1.1	SSW 1.2	S 1.2	S 1.3	ESE1 .0	SSE1 .2	SSE 1.0	N 0.9	SSE1 .1	SSE1 .0	S 1.3	S 1.1	W 0.8	SSW 0.7
23	W 0.7	WSW 0.8	SW 0.7	W 0.5	WS W0.6	W 0.6	WSW 0.7	W 0.8	WS W0.8	S 1.1	SSE 1.0	S 0.9	SE 1.0	SE 0.9	SE 0.8	SE 1.1	SSW 0.9	SE 0.8	N 0.8	S 0.8	SSW0 .9	NW 0.7	NW 0.7	NW 0.7
24	NW 0.6	SSE0 .8	SSE0 .8	SW 0.7	SSE0 .8	SSW 1.0	WSW 0.9	SW 0.9	SSE1 .2	N 1.5	S 1.1	SSE1 .2	SSE 1.1	SSE 0.8	S 0.9	SE 0.7	SE 1.0	SSE0 .8	SW 0.8	N 0.6	SSE0. 9	SSE0 .8	SE 1.0	SE 1.0
25	SSE1. 0	SSE0 .9	SSE1 .0	SSW 0.7	SSE0 .6	E 0.6	E 0.8	SE 0.9	WS W0.6	NNE1 .0	ENE 0.9	ENE0 .9	NE 1.1	NE 1.6	ENE 1.3	E 1.2	ENE 1.0	E 1.0	E 0.6	ESE0 .8	ESE0. 7	S 0.7	SSW 0.8	SSW 0.8
26	WNW 0.6	N 1.2	NNW 1.6	NNW 1.9	N 2.2	N 1.9	N 1.8	N 2.1	NNW 2.5	NNW 2.3	NN W1.9	NNW 1.9	N 2.4	N 2.6	N 2.5	N 2.5	N 2.1	NNW 1.9	N 1.2	NNW 1.2	N 1.2	NW 0.6	NNW 0.6	NNW 0.6
27	E 0.5	SSE0 .3	SW 0.3	NNW 0.1	NNW 0.1	NW 0.2	W 0.4	WS W0.3	SW 0.6	S 1.5	SSE 1.5	SSE1 .5	SE 1.5	SE 1.5	SE 1.4	SSE1 .1	SSE 1.0	SSW 0.5	C 0.0	SE 0.3	SSW0 .4	NNW 0.1	N 0.5	N 0.3
28	NNE0 .5	N 0.3	SSW 0.6	SSE0 .5	ENE 0.5	NNW 0.4	SSE0 .5	E 0.5	ENE 0.9	E 1.1	SE 1.2	ESE1 .1	ENE 1.4	NE 1.2	ENE 1.4	NE 1.0	ENE 0.9	N 0.3	NNW 0.2	NW 0.4	NW 0.4	WN W0.5	WN W0.5	WN W0.4
29	NNW 0.2	W 0.2	WN W0.2	NNE 0.3	E 0.5	W 0.3	NNW 0.1	NNE 0.2	SE 0.8	ESE1 .0	E 1.1	ESE1 .2	ESE 1.0	ESE 1.0	ENE 1.0	ENE0 .8	NE 0.5	NW 0.3	WNW 0.4	NW 0.3	WNW 0.4	WN W0.3		C 0.0
30	C 0.0	SW 0.1	C 0.0	C 0.0	N 0.1	NE 0.2	ESE0 .3	SE 0.8	S 0.9	ESE1 .2	ESE 1.2	E 1.2	ESE 1.1	ESE 0.9	SE 0.9	SE 0.6	E 0.3	NNW 0.2	NW 0.2	W 0.3	NNW 0.1	NNW 0.1	N 0.2	N 0.1
31	NW 0.2	SSE0 .1	N 0.1	ESE0 .3	SE 0.5	C 0.0	ENE0 .1	NNE 0.3	SE 0.8	ESE1 .1	E 1.1	ESE1 .1	E 1.1	ENE 1.2	ENE 1.1	ENE0 .9	NNE 0.6	N 0.4	NE 0.4	W 0.3	W 0.2	C 0.0	W 0.3	N 0.3

Table S1. Continued

Gü	0	1	2	3	4	5	6	7	8	9	10	11	12	13	14	15	16	17	18	19	20	21	22	23
1	C 0.0	C 0.0	C 0.0	C 0.0	WN W0.5	C 0.0	SSE0 .2	SE 0.9	E 1.1	NNE1 .5	NNE 1.2	ENE1 .8	NNE 2.3	E 1.3	NNE 1.3	ENE0 .7	NE 0.2	SW 0.3	W 1.0	W 0.5	W 0.4	SSW 0.4	WN W0.5	WS W0.6
2	W 0.6	NW 0.5	W 0.4	WS W0.0	W 0.4	C 0.0	ENE0 .1	N 1.2	NE 0.9	NE 1.2	ENE 1.1	E 1.3	NE 0.9	NE 0.8	ESE0 .6	SE 0.6	WN W0.5	W 0.6	W 0.6	C 0.0	SW 0.3	C 0.0	C 0.0	N 0.6
3	C 0.0	C 0.0	SSW 0.0	W 0.0	C 0.0	C 0.0	S 0.7	SSW 0.7	ENE 0.8	E 1.3	ESE 1.0	NNE 0.4	E 1.2	S 1.1	E 0.6	ESE0 .0	N 0.3	C 0.0	SSW 0.0	C 0.0	C 0.0	S 0.1	NE 0.4	C 0.0
4	C 0.0	C 0.0	S 0.1	S 0.0	SE 0.5	WSW 0.0	SE 0.4	SE 1.0	E 1.2	E 0.9	E 1.1	E 1.2	ESE 1.9	ESE 1.0	SSE0 .9	SE 0.4	C 0.0	W 0.5	C 0.0	NE 0.4	SSE0. 0	C 0.0	C 0.0	NNE 0.4
5	SW 0.3	C 0.0	C 0.0	C 0.0	SSE0 .1	S 0.4	E 0.8	SSW 0.5	SE 1.2	E 0.9	NNE 0.7	SW 0.5	S 1.0	ESE 0.7	ESE1 .0	SE 1.1	SW 0.5	C 0.0	S 0.0	SE 0.5	ESE0. 4	NW 0.3	SSW 0.1	S 0.5
6	NE 0.6	S 0.7	W 0.4	SE 0.7	S 0.4	NW 0.5	NE 0.2	E 0.5	SSW 0.5	NNE0 .9	NE 1.7	NE 0.7	SE 1.4	ENE 0.6	NE 0.2	NNE 0.1	NNE 0.2	NNE 1.8	ENE1 .8	N 0.9	NNE1 .3	SE 0.5	W 0.5	S 0.4
7	S 0.0	S 1.2	SW 0.6	WN W0.5	SE 0.6	NNW 0.1	SW 0.7	SSW 0.8	SSE1 .1	ESE1 .7	ESE 1.8	NE 1.3	ENE 1.0	SE 1.2	SSE1 .2	NNE 1.6	N 0.8	NNE 1.4	WNW 0.5	W 0.7	SSW0 .5	SSW 1.2	ESE0 .8	SSE0 .9
8	E 0.5	S 0.1	S 1.2	SSW 1.0	SE 1.1	SE 0.5	NE 0.7	SE 1.2	NNE 1.0	NNE0 .5	SSW 0.8	SSE0 .9	SSW 1.1	SE 1.6	ESE1 .7	S 1.1	SE 1.3	E 1.0	E 1.0	ENE1 .4	E 0.9	E 2.0	ESE1 .0	SSE0 .9
9	W 1.8	WN W0.7	NW 0.9	WN W0.9	W 1.1	NW 0.5	WN W0.9	W 0.8	W 0.8	SW 0.8	S 1.9	S 1.4	ENE 1.0	ESE 1.6	ESE1 .7	E 1.2	ESE 1.1	SSW 0.6	S 1.3	SSW 0.5	NW 0.3	WN W0.5	C 0.0	S 0.6
10	SE 0.8	NNW 0.3	S 0.5	NE 1.0	SE 0.5	NNE 0.8	WSW 0.8	E 1.1	ENE 0.6	SE 1.3	WN W0.6	S 1.5	SSE 1.1	ESE 0.6	NW 0.4	W 0.5	C 0.0	N 1.2	SSE0 .5	ENE0 .4	SE 0.4	S 1.0	C 0.0	S 0.4
11	N 0.6	C 0.0	SW 0.4	NNE 0.2	N 0.5	NNE 0.0	ENE0 .3	NNW 0.3	ENE 0.7	SSE2 .9	SSE 3.0	S 1.9	SE 3.3	SE 1.8	ESE1 .8	SE 3.8	SE 3.1	WN W1.2	E 1.7	ESE4 .0	SE 3.3	SSE2 .7	SSE2 .4	SE 2.9
12	SSE3. 4	SSW 2.0	ENE 1.9	SE 3.2	SE 2.1	SSE1 .1	W 0.6	SW 1.0	S 1.8	SSE2 .4	S 2.6	SSW 1.1	SSW 1.3	S 1.7	SW 1.1	SSW 0.7	W 0.6	SE 0.7	ESE0 .8	SW 0.3	C 0.0	C 0.0	WS W0.1	C 0.0
13	C 0.0	C 0.0	C 0.0	C 0.0	C 0.0	SSW 0.0	SSW 0.0	SSE0 .4	S 1.0	ESE1 .0	NNE 1.0	SSE1 .6	S 1.9	SSE 2.0	SE 1.2	SSW 2.0	S 0.9	SSE1 .8	NNW 0.6	SE 0.7	SSE0. 3	WN W0.4	S 0.0	SSW 0.3
14	WSW 0.4	C 0.0	C 0.0	C 0.0	C 0.0	C 0.0	C 0.9	W 0.6	S 0.3	SSE1 .5	W 0.9	SSW 0.9	ENE 1.4	ESE 1.0	E 1.2	S 0.8	NNE 0.0	W 0.3	N 0.0	N 0.0	WSW 0.7	C 0.0	WN W0.7	E 0.6
15	SSW0 .0	NW 0.0	C 0.0	WN W0.3	SSE0 .2	S 0.3	NW 0.0	C 0.0	SE 0.7	NW 0.5	ESE 1.1	E 0.5	SSE 1.2	E 1.6	SE 1.3	SE 0.8	C 0.0	SE 0.6	S 0.0	NNE0 .2	SSE0. 5	N 0.4	W 0.2	C 0.0
16	C 0.0	C 0.0	SSW 0.5	WS W0.0	ENE 0.3	NW 0.6	W 0.5	E 0.7	S 1.0	NE 1.0	NNE 0.6	NE 1.2	ENE 1.3	E 1.4	ESE0 .6	SE 1.1	C 0.0	WSW 0.2	SSW 0.5	C 0.0	C 0.0	SSW 0.3	SE 0.5	ENE 0.3
17	NW 0.5	WN W0.6	NW 0.6	ESE0 .6	E 0.4	NW 0.5	NE 0.6	ENE 0.4	SSE1 .6	SSE1 .3	SE 1.8	ESE2 .3	SSW 1.2	S 1.3	SSW 1.6	S 2.6	ENE 0.7	S 1.4	N 1.0	S 1.5	SSE0. 4	SE 1.0	SSW 1.0	WS W0.2
18	ESE1. 4	SSE0 .4	SSE0 .6	C 0.0	SSE0 .5	NNE 0.7	NNE 0.9	NE 0.5	S 1.3	S 2.3	SSW 2.0	SSW 3.4	S 1.2	WS W1.0	WS W1.6	E 0.8	NW 0.8	NW 0.4	NNW 0.6		S 1.2	SW 1.3	W 1.1	W 2.0
19	S 1.5	NW 1.0	E 1.4	E 1.1	NNE 1.0	N 0.5	N 0.8	NE 1.3	NE 0.9	ENE0 .9	SSE 1.4	ESE0 .9	E 0.8	SE 1.3	SW 0.3	NW 0.0	NW 0.5	NNW 0.6	NNE0 .3	NNE0 .1	WSW 0.1	SSW 0.3	W 0.4	ESE0 .1
20	S 0.7	SSW 0.1	C 0.0	C 0.0	E 0.1	W 0.7	SE 0.2	SE 0.8	SE 0.9	ENE1 .3	E 1.2	SSE1 .2	S 1.9	ESE 0.7	SE 1.8	SSE0 .9	WS W0.5	ENE1 .5	NNW 0.5	S 1.8	SSW0 .6	SE 1.0	S 0.8	SW 0.6
21	NNE0 .0	SE 0.6	NW 0.4	NE 0.5	NE 0.4	NNE 0.2	E 0.6	E 0.6	NE 0.6	NNW 0.5	S 1.2	E 0.9	E 0.9	ESE 0.6	SE 1.8	SE 0.9	SE 0.4	C 0.0	C 0.0	C 0.0	NE 0.5	W 0.0	W 0.8	N 0.4
22	ESE0. 7		WN W0.0	S 0.4	C 0.0	C 0.0	SSE0 .1	NE 0.6	NNE 0.6	N 1.1	S 0.8	SE 1.3	SE 1.3	ESE 1.0	NW 0.9	NE 0.4	C 0.0	WN W0.3	WNW 0.0	C 0.0	W 0.4	WS W0.4	SSW 0.0	NW 0.3
23	NW 0.5	WN W0.2	C 0.0	NW 0.4	E 0.4	E 0.1	SE 0.8	ESE0 .3	SSW 0.8	ENE1 .2	ENE 0.8	ESE1 .8	E 2.2	NNE 1.3	NE 1.3	E 1.1	ENE 0.6	SSW 0.3	SSW 0.3	C 0.0	C 0.0	C 0.0	NW 0.4	NW 0.4
24	WNW 0.5	W 0.3	NE 0.2	S 0.6	C 0.0	SW 0.0	SE 0.3	SE 0.7	NNE 0.7	ENE1 .8	ENE 2.2	WSW 0.8	N 1.0	E 1.4	NE 0.8	E 1.1	C 0.0	NNE 0.4	SSW 0.5	SW 0.1	W 0.7	W 0.6	WN W0.2	C 0.0
25	WNW 0.8	WN W0.3	C 0.0	C 0.0	C 0.0	NNE 0.4	NNE 0.8	ESE1 .0	E 2.0	ESE2 .0	E 1.8	E 2.8	E 2.6	C 2.7	NE 1.6	NE 2.0	NW 1.1	NNE 0.8	C 0.0	SSW 0.3	SSW0 .6	C 0.0	SSW 0.6	C 0.0

Section 3. CEREVE procedure

The cleaning of the materials (holding in a 2% HCl bath for 1 day followed by rinsing with ultra-pure water) and the pre-cleaning processes for the cellulose filters were carried out outside of the clean room. The storage of the filters in the desiccator until analysis and the storage of clean sampling containers were conducted in the clean room.

Section 4. Sample preparation and physicochemical analyses

Sample preparation

Before use, Whatman GF/A filters (TSPs and TDs 37, 90 mm, respectively) were cleaned in a furnace at 550°C for approximately 5 hours (Figure S2). Macherey-Nagel MN 640 cellulose filters (125 mm) were rinsed twice with 150 ml of ultra-pure water before each use and then dried in an oven at 105±2°C for 1 hour (Figure S3). Millipore

MCE-HAWG04700 hydrophilic cellulose esters membrane filters and HDPE collection bottles were cleaned by soaking in a 2% nitric acid solution (24 h) and then in an ultra-pure water bath (12 h) in a clean room.

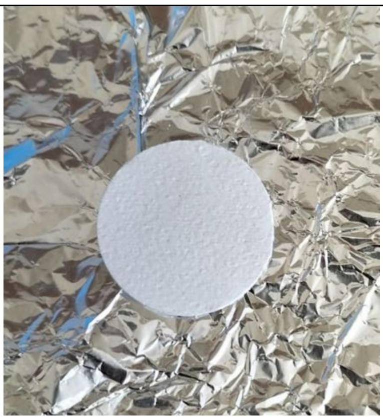<br>A-GF/A filter for TSP	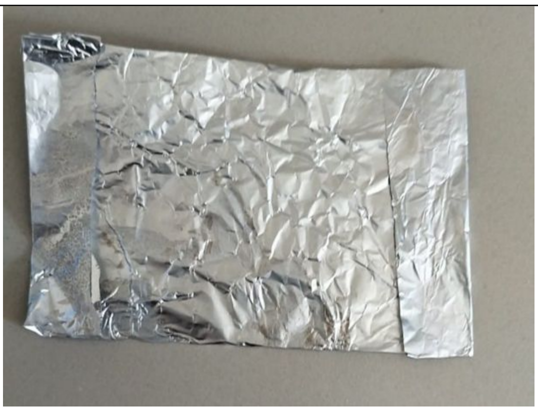<br>B- Wrapping in Al foil for the oven
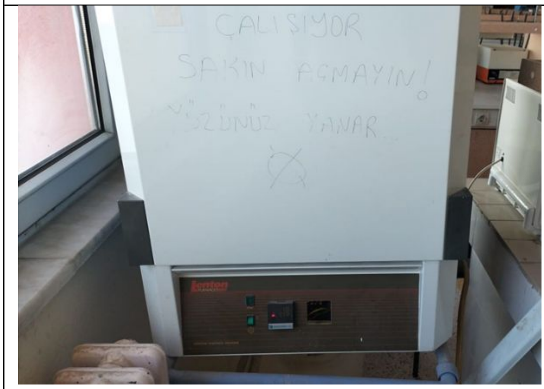<br><br>C- The furnace (550 ^{o}C'de 5 hour)	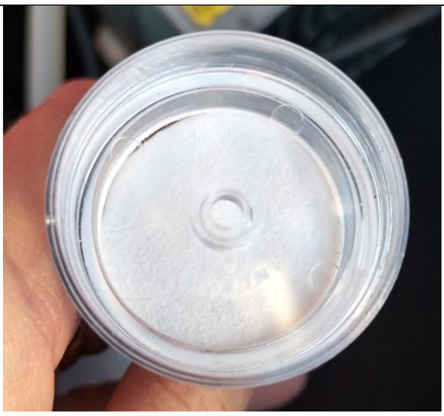<br>D- Placing the filter paper inside the measurement device (TSP)

Figure S2. Pre-preparation of GFA filters

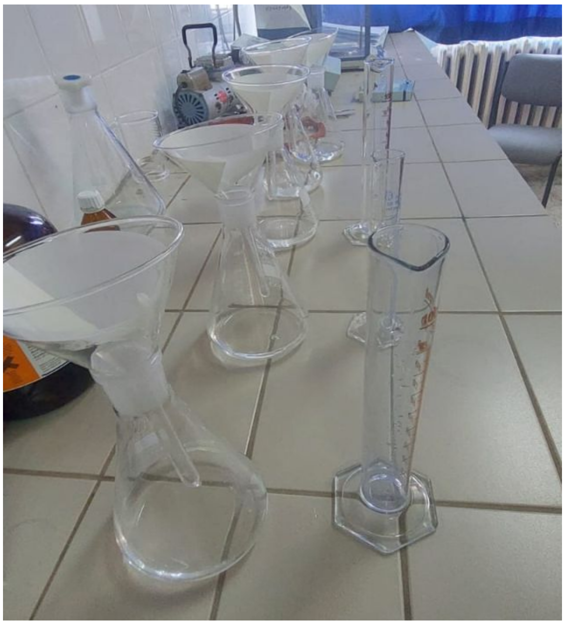

Figure S3. Pre-preparation of cellulose

In the clean room, the materials were dried on the bench. They were preserved in double plastic bags until sampling/analysis. The blank/sampling filters were placed in glass petri dishes, wrapped with parafilm, and transported to the sampling area.

The analyses of water-soluble/insoluble organic matter (WOM/WIOM and ash (WSA/WIA) of TDS were carried out using the loss-on-ignition (LOI) method (Boon et al., 1998). In LOI, the residue on the filter represents the insoluble phase, while the soluble phase is the filtrate. The filtrate, placed in pre-weighed porcelain crucibles, was first dried in an oven at 180 °C (12–24 h) to obtain gravimetric TDS (EPA 160.1), and then incinerated at 550 °C for WOM and WSA analyses. A similar procedure was applied to cellulose filters. The cellulose filters were dried in an oven at 105 °C (1 h) to obtain TSS (EPA 160.2), followed by incineration at 550 °C for WIOM and WIA analyses. The weights of each filter/crucible were measured after stabilizing for 24 hours (desiccator with silica gel, 20-22 °C) and were carried out using a Scaltec microbalance with a sensitivity of 10 µg in a temperature and humidity-controlled microenvironment.

Section 5. Extraction-solvent change

The organic and inorganic eluates of the samples were obtained using ultrasonic extraction method with dichloromethane (DCM, Merck) and ultra-pure water (Milipore® Water Purification System-18 MΩ at 25◦C), respectively. GFA filters, which were cut into small, equal pieces, were placed in beakers with ultra-pure water (50 ml) in the inorganic eluates and DCM (150 ml) in the organic eluates. The beakers were covered and applied at the extraction process at room temperature (Figure S4-A.). After passing through a membrane filter (Millipore), the dilution series of inorganic eluates were prepared. They were concentrated using a rotary evaporator (Figure S4-B). DCM was evaporated in a water bath (Figure S4-C.).

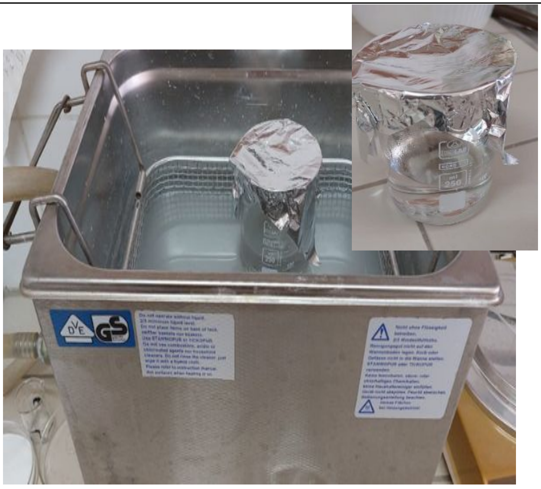

A- Ultrasonic extraction

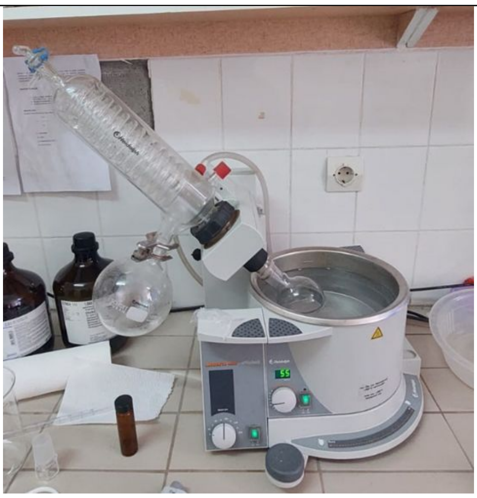

B- Rotary Evaporator

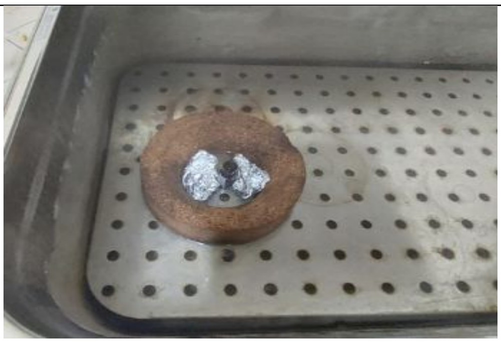

C- Water bath

Figure S4**.** Extraction**-**solvent change procedure

Section 6. Ecotoxicity analysis

The higher plant Lepidium sativum (purchased from the local market (Verve) and stored in the desiccator until analysis) was used as a test plant for evaluating the acute toxicity of the inorganic and organic fractions of TDs and TSPs. The validity of the test was controlled with a reference test (potassium dichromate, 3.2–56 mg/L, 6 concentration series) for each seed package. Some of the results (1 package) obtained are presented in Table S2. To summarize briefly, 5 mL of control/sample solution (inorganic control: ultra-pure water, organic control: %1.5 DMSO/samples-dilution series of 6.25%, 12.5%, 25%, 50%, 100%) and 25 healthy-looking Lepidium sativum seeds were placed at equal intervals on a 9 cm filter paper. The seeded glass petri dishes were covered and incubated in the darkness at 25±1°C for 3 days. Biometric indices (number of germinated seeds, root length-stem height of the 20 best-growing seeds in 25 seeds) were recorded in a logbook (Figure S5.). A seed was considered germinated if at least 1 mm of root length was observed.

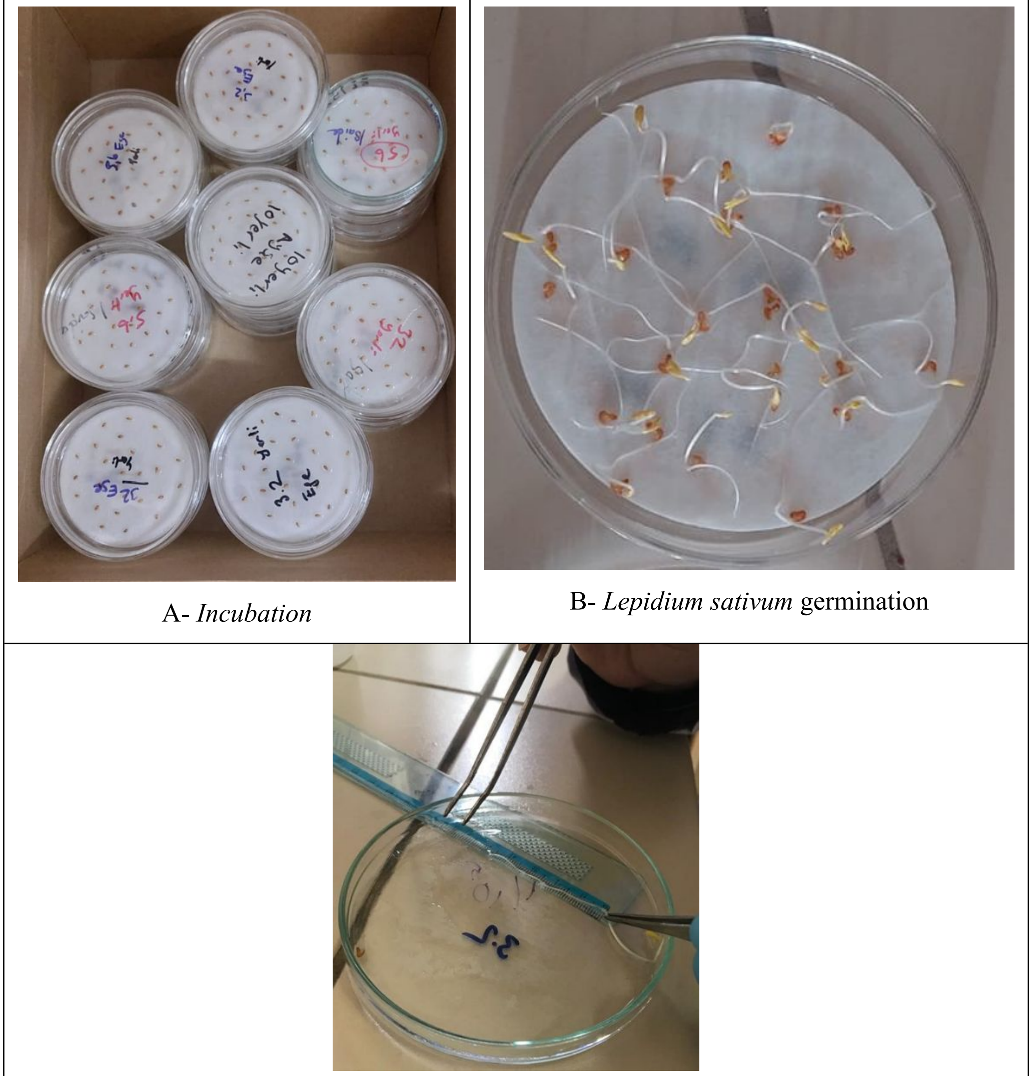

A- *Incubation*

B- *Lepidium sativum* germination

C- Measuring

Figure S5. *Lepidium sativum* toxicity test

Table S2. Reference test results

**LEPIDIUM SATIVUM**																								
Seed Charge:	Verve																							
Date:																								
Name:	Gülnihal Kara																							
Sample Name:	5 mL synth. potassium dichromate n=3																							
Control:	5 mL seralpur n=6																							
**KONTROLLEN**																								
Control 1	1	2	3	4	5	6	7	8	9	10	11	12	13	14	15	16	17	18	19	20	MV [cm]	STD DEV [cm]	VAR KOE	
Root length	5,8	5,6	4,3	7,1	5,4	3,9	5,2	4,9	6,1	5,5	5,5	5,8	5,5	5,5	3,9	6,2	5,5	5,0	5,6	4,9	*5,36*	0,74	13,74	
Hypokotyl length	3,0	3,4	3,3	3,7	2,7	2,6	2,7	2,5	3,0	2,6	2,6	2,2	2,6	2,8	2,0	2,9	2,5	2,9	2,0	2,5	2,73	0,42	15,35	
Control 2																								
Root length	6,0	6,3	5,6	4,8	6,2	5,1	6,1	6,5	5,2	6,7	3,7	5,2	5,9	3,1	6,0	5,0	4,9	5,9	5,7	6,7	*5,53*	0,91	16,51	
Hypokotyl length	3,0	3,1	3,3	2,7	2,2	3,0	3,0	2,6	3,4	3,5	2,2	3,0	2,7	2,5	2,5	2,1	2,6	3,1	2,2	2,9	*2,78*	0,41	14,59	
Control 3																								
Root length	5,9	4,8	4,5	5,0	5,1	4,5	4,7	5,4	4,9	5,5	4,3	5,2	5,5	3,5	5,1	5,0	5,5	5,5	5,5	5,1	*5,03*	0,53	10,58	
Hypokotyl length	3,0	3,3	3,6	3,1	2,6	3,3	3,3	2,2	2,5	2,6	2,2	3,2	2,6	3,3	2,3	2,7	2,6	2,6	2,6	2,7	2,82	0,40	14,31	
Control 4																								
Root length	6,0	6,3	5,6	4,8	6,2	5,1	6,1	6,5	5,2	6,7	5,5	5,2	5,9	5,5	6,0	5,0	4,9	5,9	5,7	6,7	*5,74*	0,57	9,95	
Hypokotyl length	3,0	3,1	3,3	2,7	2,2	3,0	3,0	2,6	3,4	3,5	2,6	3,0	2,7	2,6	2,5	2,1	2,6	3,1	2,2	2,9	*2,8*	0,4	13,6	
Control 5																								
Root length	6,0	6,3	5,6	4,8	6,2	5,1	6,1	6,5	5,2	6,7	3,7	5,2	5,9	3,1	6,0	5,0	4,9	5,9	5,7	6,7	*5,5*	0,9	16,5	
Hypokotyl length	3,0	3,1	3,3	2,7	2,2	3,0	3,0	2,6	3,4	3,5	2,2	3,0	2,7	2,5	2,5	2,1	2,6	3,1	2,2	2,9	*2,8*	0,4	14,6	
Control 6																								
Root length	6,0	6,3	5,6	4,8	6,2	5,1	6,1	6,5	5,2	6,7	5,5	5,2	5,9	5,5	6,0	5,0	4,9	5,9	5,7	6,7	5,7	0,6	10,0	
Hypokotyl length	3,0	3,1	3,3	2,7	2,2	3,0	3,0	2,6	3,4	3,5	2,6	3,0	2,7	2,6	2,5	2,1	2,6	3,1	2,2	2,9	2,8	0,4	13,6	
				**C1**	**C2**	**C3**	**C4**	**C5**	**C6**															
Mean Root Length				*5,36*	*5,53*	*5,03*	*5,74*	*5,53*	*5,74*												***5,49***			
Mean Hypokotyl Length				2,73	2,78	2,82	2,81	2,78	2,81												**2,79**			
**conc 1**	32 mg/L																							
sample 1																					MW [cm]	STD DEV [cm]	VAR KOE	Inhibition [%]
Root length	3,1	2,3	1,7	2,0	0,7	1,8	1,7	0,6	1,1	1,0	0,2	1,7	1,1	1,0	1,2	1,6	1,0	0,6	0,8	0,7	*1,30*	0,67	52,00	
Hypokotyl length	2,4	2,5	1,4	1,8	1,6	1,6	1,1	2,5	1,5	1,5	1,4	2,0	1,8	1,2	1,6	1,8	1,5	1,6	0,6	0,7	*1,61*	0,49	30,74	
sample 2																								
Root length	2,0	1,4	0,8	1,2	0,9	1,2	1,4	1,0	1,2	1,0	0,6	1,4	1,1	1,2	1,2	0,6	1,1	0,7	0,8	1,2	*1,10*	0,32	29,03	
Hypokotyl length	2,1	1,6	1,5	1,6	1,5	1,6	1,5	1,6	1,4	1,8	1,3	1,2	1,7	0,8	1,6	1,1	1,4	1,7	1,8	1,6	*1,52*	0,27	17,85	
Sample 3																								
Root length	1,5	2,1	1,4	1,6	1,3	1,9	1,6	1,4	1,2	1,6	1,5	0,6	1,4	1,6	0,9	0,8	1,2	1,4	1,0	0,8	*1,34*	0,37	27,46	
Hypokotyl length	2,0	1,7	2,5	2,3	2,2	2,4	2,7	1,8	1,5	1,4	2,8	2,6	2,4	2,5	2,3	2,2	2,6	2,3	2,2	2,2	*2,23*	0,37	16,72	
				**S1**	**S2**	**S3**																		
Mean Root Length				*1,30*	*1,10*	*1,34*															**1,25**			**77,31**
Mean Hypokotyl Length				1,61	1,52	2,23															**1,79**			**35,91**
**conc 2**	56 mg/L																							
sample 1																								
Root length	1,0	1,6	0,6	0,7	0,5	0,5	0,7	0,4	0,4	1,1	0,7	0,4	1,8	0,4	0,8	0,5	1,4	0,2	0,4	0,9	*0,8*	0,4	56,5	
Hypokotyl length	0,9	1,0	1,6	1,6	1,0	0,6	2,0	1,0	1,0	1,0	0,6	0,6	0,5	0,6	0,6	0,6	1,1	1,4	0,6	0,8	0,96	0,40	42,34	
Sample 2																								
Root length	0,9	1,2	0,6	0,6	0,3	0,3	0,5	0,6	0,5	0,3	0,6	0,3	0,3	0,3	0,2	0,5	0,4	0,4	0,5	0,2	*0,48*	0,24	49,76	
Hypokotyl length	0,6	1,6	1,3	1,2	1,1	0,6	0,7	0,8	1,2	0,9	0,8	0,7	0,6	0,7	0,5	1,1	0,6	0,7	0,8	0,6	0,86	0,29	33,79	
Sample 3																								
Root length	0,6	0,6	0,4	0,4	0,8	0,7	0,2	0,6	0,3	0,7	0,2	0,4	1,1	0,6	0,3	0,5	0,6	2,1	0,8	0,6	*0,6*	0,4	64,2	
Hypokotyl length	0,8	0,7	0,8	0,6	1,0	0,6	1,1	0,8	1,2	1,1	0,5	1,2	1,2	0,6	0,8	0,6	0,9	1,2	0,7	1,1	0,88	0,24	27,01	
				**S1**	**S2**	**S3**																		
Mean Root Length				*0,75*	*0,48*	*0,63*															**0,62**			**88,76**
Mean Hypokotyl Length				0,96	0,86	0,88															**0,90**			**67,86**
**conc 3**	18 mg/L																							
sample 1																					MW [cm]	STD DEV [cm]	VAR KOE	Inhibition [%]
Root length	2,3	2,6	2,7	3,2	2,0	2,1	2,1	2,2	2,1	2,9	2,7	1,8	1,9	1,3	2,2	1,7	1,9	1,9	1,6	1,5	*2,14*	0,47	22,18	
Hypokotyl length	2,2	2,8	2,8	2,9	2,1	2,7	3,4	2,4	1,9	1,8	2,4	1,9	2,3	1,4	1,8	2,1	2,2	2,1	1,9	1,6	*2,24*	0,48	21,42	
sample 2																								
Root length	3,2	2,0	1,5	1,5	2,4	1,8	1,7	2,3	2,3	1,7	1,3	2,3	1,7	1,7	2,0	2,2	1,3	1,4	1,6	1,5	*1,87*	0,46	24,57	
Hypokotyl length	2,7	2,5	2,2	2,0	2,3	2,0	2,2	2,6	2,0	2,2	2,1	2,0	1,8	1,1	2,0	2,1	2,1	1,2	1,4	1,3	*1,99*	0,43	21,55	
sample 3																								
Root length	2,3	1,8	2,2	2,0	2,0	1,2	0,5	1,7	2,0	3,0	1,2	1,6	2,2	3,3	1,4	1,2	1,9	0,9	1,3	1,9	*1,78*	0,65	36,48	
Hypokotyl length	2,5	2,0	1,7	2,3	2,5	2,1	1,2	1,7	2,2	2,0	1,9	2,2	1,5	2,0	1,6	1,8	1,7	1,1	1,3	1,7	*1,85*	0,39	20,90	
				**S1**	**S2**	**S3**																		
Mean Root Length				*2,14*	*1,87*	*1,78*															***1,93***			**64,86**
Mean Hypokotyl Length				2,24	1,99	1,85															**2,03**			**27,29**

Table S2. Continued

**conc 4**	10 mg/L																							
Sample 1																								
Root length	3,3	3,7	4,1	3,6	3,0	4,3	2,6	4,3	3,6	2,8	2,1	3,3	2,8	2,3	2,9	2,3	3,2	2,6	2,1	1,6	*3,03*	0,74	24,49	
Hypokotyl length	2,8	3,2	3,0	2,5	2,5	3,0	2,0	2,9	2,5	2,5	1,9	2,2	1,9	1,8	2,5	1,9	2,9	2,0	1,8	1,4	*2,36*	0,49	20,90	
Sample 2																								
Root length	2,4	2,2	1,7	2,0	3,4	2,2	2,8	2,9	2,9	1,8	3,4	2,3	2,8	2,5	1,0	2,6	2,1	3,3	2,3	3,0	*2,48*	0,59	23,98	
Hypokotyl length	2,0	2,1	1,8	1,8	2,8	1,3	2,3	3,0	1,9	2,3	2,4	1,6	3,0	2,1	1,0	2,2	1,7	1,8	1,8	1,9	*2,04*	0,50	24,33	
Sample 3																								
Root length	2,7	3,2	2,8	2,7	3,0	3,0	2,4	2,8	2,1	2,8	3,3	2,5	2,2	1,5	2,3	3,2	1,4	1,9	1,8	1,6	*2,46*	0,57	23,37	
Hypokotyl length	2,6	2,5	2,6	1,9	2,8	2,6	1,9	1,3	2,8	2,7	2,9	2,3	2,3	2,1	2,5	2,6	1,9	2,3	1,9	2,1	*2,33*	0,40	17,07	
				**S1**	**S2**	**S3**																		
Mean Root Length				*3,03*	*2,48*	*2,46*															***2,66***			**51,62**
Mean Hypokotyl Length				2,36	2,04	2,33															**2,24**			**19,45**
**conc 5**	5,6 mg/L																							
Sample 1																					**MW [cm]**	**STD DEV [cm]**	**VAR KOE**	**Inhibition [%]**
Root length	4,5	5,4	4,7	4,6	6,4	5,0	4,6	4,0	3,6	4,0	4,5	3,9	3,0	4,1	4,2	3,6	3,9	2,2	2,9	2,6	*4,09*	0,95	23,28	
Hypokotyl length	2,2	3,0	2,0	3,0	3,0	2,5	2,4	1,7	2,6	1,6	1,8	2,5	1,9	2,3	2,1	1,5	2,5	1,9	1,8	1,5	2,19	0,48	21,75	
Sample 2																								
Root length	5,0	3,0	4,0	5,6	4,4	4,2	4,6	5,5	4,5	2,9	3,1	6,8	3,9	3,2	3,0	3,2	2,5	3,6	1,7	2,5	*3,86*	1,21	31,37	
Hypokotyl length	3,1	2,7	3,0	3,9	3,7	2,5	3,6	3,0	3,0	3,1	2,9	3,0	2,5	2,4	2,4	2,5	2,5	1,9	1,5	2,0	*2,76*	0,58	21,14	
Sample 3																								
Root length	5,2	5,3	1,4	4,5	4,7	5,1	4,6	2,1	1,7	5,5	1,1	2,4	2,4	2,8	4,6	3,2	4,6	4,5	1,6	1,4	*3,44*	1,52	44,18	
Hypokotyl length	2,4	2,4	1,2	2,4	2,3	2,6	2,5	2,0	1,4	2,1	1,5	1,8	1,6	1,5	1,9	1,4	2,6	2,3	1,4	1,9	*1,96*	0,45	22,95	
				**S1**	**S2**	**S3**																		
Mean Root Length				*4,09*	*3,86*	*3,44*															***3,79***			***30,87***
Mean Hypokotyl Length				2,19	2,76	1,96															**2,30**			***17,30***
**conc 6**	3,2 mg/L																							
Sample 1																								
Root length	5,3	5,3	4,5	5,6	5,4	3,3	5,2	5,3	5,3	5,4	3,2	5,3	3,2	5,3	5,2	3,2	5,3	5,7	5,3	3,2	*4,78*	0,92	19,34	
Hypokotyl length	3,3	3,3	2,4	3,1	2,8	2,7	2,9	3,3	3,3	3,2	2,6	3,3	2,7	3,3	3,4	2,6	3,3	3,5	3,3	3,1	3,07	0,32	10,31	
Sample 2																								
Root length	4,9	5,2	6,1	6,2	5,5	3,4	6,4	3,2	5,4	4,3	4,4	5,3	5,4	3,2	5,3	4,0	5,3	3,2	3,2	5,3	*4,76*	1,04	21,94	
Hypokotyl length	2,8	3,1	2,8	3,4	2,4	3,5	2,7	3,2	3,3	2,1	2,5	3,3	2,9	3,4	3,3	2,8	3,3	3,1	3,3	3,3	*3,03*	0,37	12,36	
Sample 3																								
Root length	4,6	5,3	3,1	5,3	4,6	5,3	3,4	4,2	3,9	3,4	4,3	4,5	5,4	3,9	4,2	4,1	5,3	2,9	3,1	3,5	*4,22*	0,80	18,95	
Hypokotyl length	3,5	3,3	2,7	3,3	2,6	3,3	3,2	3,7	2,9	2,7	2,8	3,1	3,5	3,3	3,1	2,8	3,3	2,6	3,4	2,7	*3,09*	0,33	10,68	
				**S1**	**S2**	**S3**																		
Mean Root Length				*4,78*	*4,76*	*4,22*															***4,58***			**16,48**
Mean Hypokotyl Length				3,07	3,03	3,09															**3,06**			**-9,93**

In the control/dilution series of inorganic/organic eluates, 100% seed germination was detected, supplying the validity criteria (OECD, 2006). The tests were conducted with 3 replicates for sample dilution series and 6 replicates for control series. The effects of the eluates on root elongation and stem height were compared according to the control (% inhibition) and calculated according to Equation 1.

%effect = (A−B)/A *100, where A is the control and B is the measured average root length/stem height in the dilution series of organic/inorganic eluates (Microbiotests Inc., 2016).

In the evaluation was used to sets that supplied the validity criteria in ISO 11269-1. Results related to the samples (Table 3) were used to calculate the inhibitory effect.

Table S3. The results of the samples

BA_{TD} -Inorganic Eluat

**LEPIDIUM SATIVUM**																								
Seed Charge:																								
Date:	10.03.2023																							
Name:	Ali ÇANKAYA																							
Sample Name:	8 mL yerli filtre kağıtı 6,25- 12,5-25-50-100 mg/l																							
Control:	8 mL seralpur n=3																							
**KONTROLLEN**																								
Control 1	1	2	3	4	5	6	7	8	9	10	11	12	13	14	15	16	17	18	19	20	**MV [cm]**	**STD DEV [cm]**	**VAR KOE**	
Root length	5,0	6,2	5,5	4,6	6,8	6,6	4,0	5,5	4,4	6,2	5,0	6,1	5,7	3,5	5,0	3,9	3,8	4,8	3,7	4,9	*5,06*	0,97	19,25	
Hypokotyl length	3,3	3,4	3,1	3,2	2,6	2,8	3,7	2,4	3,4	3,2	2,6	3,0	2,9	2,4	3,0	2,5	3,0	2,1	2,8	2,7	2,91	0,39	13,53	
Control 2																								
Root length	6,7	4,7	4,6	6,3	5,7	4,7	4,7	4,7	3,9	4,7	3,4	4,7	3,6	4,7	4,7	3,0	4,7	4,7	3,1	4,7	*4,60*	0,90	19,66	
Hypokotyl length	3,7	2,8	4,0	3,1	3,5	2,4	3,9	3,9	2,3	2,7	3,2	3,9	2,8	3,9	2,0	2,9	3,9	3,9	2,1	3,9	*3,24*	0,68	20,87	
*Control 3 **(filtre)***																								
Root length	5,2	6,3	6,0	3,6	5,5	4,7	4,1	4,1	5,0	3,0	4,8	4,9	3,6	5,6	5,2	3,3	4,7	3,2	4,5	4,5	*4,59*	0,91	19,74	
Hypokotyl length	4,1	2,5	2,1	2,7	2,4	2,8	3,4	2,4	2,1	2,2	2,3	2,9	2,7	3,2	2,9	2,1	2,4	3,4	2,4	2,4	2,67	0,51	19,17	
Control 4																								
Root length	6,7	4,7	4,6	6,3	5,7	4,7	3,7	4,7	3,9	4,7	3,4	4,7	3,6	4,7	4,7	3,0	4,7	4,7	4,7	4,7	*4,63*	0,86	18,64	
Hypokotyl length	3,7	2,8	4,0	3,1	3,5	2,4	2,7	3,9	2,3	2,7	3,2	3,9	2,8	3,9	2,0	2,9	3,9	3,9	3,9	3,9	*3,3*	0,6	19,5	
Control 5																								
Root length	5,0	6,2	5,5	4,6	6,8	6,6	4,0	5,5	4,4	6,2	5,0	6,1	5,7	3,5	5,0	3,9	3,8	4,8	3,7	4,9	*5,1*	1,0	19,2	
Hypokotyl length	3,3	3,4	3,1	3,2	2,6	2,8	3,7	2,4	3,4	3,2	2,6	3,0	2,9	2,4	3,0	2,5	3,0	2,1	2,8	2,7	*2,9*	0,4	13,5	
Control 6																								
Root length	5,0	6,2	5,5	4,6	6,8	6,6	4,0	5,5	4,4	6,2	5,0	6,1	5,7	3,5	5,0	3,9	3,8	4,8	3,7	4,9	5,1	1,0	19,2	
Hypokotyl length	3,3	3,4	3,1	3,2	2,6	2,8	3,7	2,4	3,4	3,2	2,6	3,0	2,9	2,4	3,0	2,5	3,0	2,1	2,8	2,7	2,9	0,4	13,5	
				**C1**	**C2**	**C3**	**C4**	**C5**	**C6**															
Mean Root Length				*5,06*	*4,60*	*4,59*	*4,63*	*5,06*	*5,06*												***4,75***			
Mean Hypokotyl Length				2,91	3,24	2,67	3,27	2,91	2,91												**2,94**			
**6,25**																								
Sample 1																					**MW [cm]**	**STD DEV [cm]**	**VAR KOE**	**Inhibition [%]**
Root length	5,6	5,6	4,9	6,0	5,4	6,2	5,4	4,9	4,6	4,6	5,6	3,8	3,6	4,1	3,9	5,2	4,7	4,3	4,0	4,1	*4,83*	0,76	15,66	
Hypokotyl length	3,4	3,6	3,1	2,5	3,5	3,2	4,0	3,0	3,1	2,4	3,4	3,0	3,0	2,9	2,9	2,8	3,5	2,0	2,8	3,6	*3,09*	0,46	14,79	
Sample 2																								
Root length	4,6	5,5	4,3	6,0	5,1	5,6	4,7	5,7	4,6	4,0	5,3	4,4	3,6	4,7	4,8	5,2	5,1	4,2	3,8	4,9	*4,81*	0,63	13,10	
Hypokotyl length	3,7	3,1	2,7	3,9	2,6	3,7	3,7	3,4	3,6	3,8	2,5	3,0	3,4	3,9	2,1	3,1	2,5	3,5	3,2	2,5	*3,20*	0,54	16,81	
Sample 3																								
Root length	4,9	3,6	5,0	6,2	5,0	7,6	5,2	4,2	6,6	4,6	4,9	4,6	5,1	4,1	4,2	4,8	5,0	4,0	4,8	5,6	*5,00*	0,91	18,24	
Hypokotyl length	3,7	3,2	3,1	3,1	2,7	3,7	3,2	3,2	2,9	2,8	3,0	3,4	3,6	3,6	2,7	2,2	2,4	2,7	2,3	2,9	*3,02*	0,44	14,46	
				**S1**	**S2**	**S3**																		
Mean Root Length				*4,83*	*4,81*	*5,00*															**4,88**			**-2,67**
Mean Hypokotyl Length				3,09	3,20	3,02															**3,10**			**-5,50**
**12,5**																								
Sample 1																								
Root length	5,9	5,1	6,2	6,4	7,5	5,9	4,9	4,2	4,6	4,9	5,6	6,4	5,6	6,3	5,0	5,9	4,2	4,7	4,2	4,3	*5,4*	0,9	16,6	
Hypokotyl length	3,5	3,2	3,2	2,7	3,7	2,4	2,9	2,5	3,3	3,7	3,2	3,3	2,4	3,5	2,8	3,0	3,4	2,3	2,3	3,2	3,03	0,45	14,89	
Sample 2																								
Root length	5,1	3,8	3,7	6,6	4,1	3,6	4,7	4,6	4,0	4,5	4,1	5,7	3,7	4,9	3,9	4,3	4,7	4,9	3,5	6,0	*4,52*	0,82	18,12	
Hypokotyl length	3,1	3,7	2,7	2,8	3,2	2,9	3,6	2,9	3,6	3,1	2,6	3,4	3,2	3,3	3,3	2,8	3,1	2,4	3,3	3,6	3,13	0,35	11,25	
Sample 3																								
Root length	5,7	6,1	4,9	5,5	6,4	5,5	6,5	7,0	5,4	6,0	5,6	5,0	5,3	5,4	5,7	4,8	4,8	4,5	5,2	5,6	*5,5*	0,6	11,0	
Hypokotyl length	3,6	2,5	2,6	2,5	3,4	3,1	3,3	3,2	3,0	2,2	2,5	3,5	2,5	2,8	3,1	2,6	3,5	3,1	3,1	2,4	2,93	0,41	14,17	
				**S1**	**S2**	**S3**																		
Mean Root Length				*5,39*	*4,52*	*5,55*															**5,15**			**-8,46**
Mean Hypokotyl Length				3,03	3,13	2,93															**3,03**			**-3,01**
**25**																								
Sample 1																					**MW [cm]**	**STD DEV [cm]**	**VAR KOE**	**Inhibition [%]**
Root length	5,9	5,3	5,6	4,5	4,5	7,1	6,6	5,8	6,2	6,4	6,9	6,4	6,0	5,9	6,4	6,3	5,3	5,4	5,0	5,7	*5,86*	0,70	11,91	
Hypokotyl length	2,8	3,4	3,2	2,6	2,4	3,5	4,4	3,2	3,3	3,2	3,7	2,5	3,4	3,7	4,5	4,3	3,4	2,9	2,1	2,5	*3,25*	0,65	20,00	
Sample 2																								
Root length	5,9	5,5	4,7	6,2	5,2	4,6	6,6	3,0	4,6	5,6	4,0	5,5	5,4	4,5	5,8	5,0	5,6	3,9	4,1	5,7	*5,07*	0,86	17,05	
Hypokotyl length	3,0	3,1	4,5	4,4	3,2	2,9	3,1	2,9	2,7	2,9	2,1	3,5	2,9	2,8	2,4	2,6	3,0	2,5	3,0	3,6	*3,06*	0,57	18,76	
Sample 3																								
Root length	4,6	4,6	4,6	4,6	5,2	5,4	4,6	5,1	4,6	4,9	4,9	4,6	4,6	3,6	6,2	5,6	5,3	5,3	4,1	3,1	*4,78*	0,67	13,94	
Hypokotyl length	3,5	2,9	2,8	2,9	3,1	3,2	2,8	3,1	3,1	3,7	3,1	2,8	3,5	2,1	2,1	3,0	2,1	2,6	3,0	2,5	*2,90*	0,44	15,11	
				**S1**	**S2**	**S3**																		
Mean Root Length				*5,86*	*5,07*	*4,78*															***5,24***			**-10,21**
Mean Hypokotyl Length				3,25	3,06	2,90															**3,07**			**-4,37**

Table S3. Continued

**50**																								
Sample 1																								
Root length	6,3	6,3	5,8	5,9	6,5	5,8	5,2	4,1	5,3	4,7	6,2	6,7	4,6	4,2	5,5	6,0	4,0	3,5	4,9	3,6	*5,26*	0,98	18,57	
Hypokotyl length	3,1	3,1	2,3	3,6	3,4	2,5	3,1	3,3	2,5	2,7	2,9	3,0	2,5	2,6	3,0	3,0	2,0	2,0	2,0	2,3	*2,75*	0,47	16,95	
Sample 2																								
Root length	5,7	6,1	5,3	6,6	6,5	6,5	4,2	5,9	5,8	4,5	4,2	4,1	4,5	4,0	4,0	3,5	4,5	4,5	4,5	4,5	*4,97*	0,95	19,15	
Hypokotyl length	4,1	3,5	3,3	3,5	3,1	3,1	3,0	3,8	2,0	2,2	2,1	3,1	2,2	2,0	3,0	2,6	2,2	2,2	2,2	2,2	*2,77*	0,64	23,15	
Sample 3																								
Root length	5,3	6,3	5,3	6,3	6,5	6,3	6,3	4,5	5,3	5,7	5,6	4,6	4,8	5,7	4,7	6,3	5,7	5,8	5,6	5,7	*5,62*	0,60	10,73	
Hypokotyl length	3,2	3,1	3,3	3,1	3,4	3,1	3,1	3,0	3,0	2,7	2,3	3,6	3,4	2,7	3,7	3,1	2,7	3,2	3,5	2,7	*3,10*	0,34	10,98	
				**S1**	**S2**	**S3**																		
Mean Root Length				*5,26*	*4,97*	*5,62*															***5,28***			**-11,16**
Mean Hypokotyl Length				2,75	2,77	3,10															**2,87**			**2,33**

**100**																								
Sample 1																					**MW [cm]**	**STD DEV [cm]**	**VAR KOE**	**Inhibition [%]**
Root length	5,4	4,5	5,5	6,5	6,4	5,4	4,6	5,2	6,1	6,2	6,3	6,2	6,9	5,2	5,2	4,9	6,1	5,0	5,5	4,7	*5,59*	0,68	12,20	
Hypokotyl length	3,3	3,2	3,4	3,1	3,3	2,9	2,5	3,4	3,6	3,1	3,1	2,9	2,7	2,8	3,8	3,5	3,4	2,7	3,0	2,9	3,13	0,33	10,46	
Sample 2																								
Root length	5,4	5,6	7,4	5,3	5,7	5,8	5,2	4,3	4,5	4,4	5,0	5,7	4,1	4,3	4,4	4,1	5,7	6,0	6,7	6,7	*5,32*	0,92	17,29	
Hypokotyl length	4,2	3,1	3,3	3,0	3,7	4,0	3,9	3,1	2,9	2,6	1,9	3,7	3,0	2,5	2,6	2,6	3,7	2,7	2,7	2,7	*3,10*	0,59	18,94	
Sample 3																								
Root length	5,7	6,0	5,6	6,5	6,5	6,2	5,6	5,0	5,6	6,3	5,0	5,2	4,8	5,6	5,2	4,8	5,9	4,6	4,9	5,6	*5,53*	0,57	10,26	
Hypokotyl length	3,6	2,4	4,4	3,6	3,4	2,8	3,6	2,9	2,8	2,7	2,8	3,4	3,9	3,4	3,0	3,1	3,4	2,6	2,4	3,8	*3,20*	0,52	16,30	
				**S1**	**S2**	**S3**																		
Mean Root Length				*5,59*	*5,32*	*5,53*															***5,48***			***-15,33***
Mean Hypokotyl Length				3,13	3,10	3,20															**3,14**			***-6,92***

Table S3. Continued

BA_{TD}-Organic eluat

**LEPIDIUM SATIVUM**																								
Seed Charge:																								
Date:																								
Name:																								
Sample Name:	Kampüs Toz Organik																							
Control:	6																							
**KONTROLLEN**																								
Control 1	1	2	3	4	5	6	7	8	9	10	11	12	13	14	15	16	17	18	19	20	**MV [cm]**	**STD DEV [cm]**	**VAR KOE**	
Root length	6,0	5,0	2,5	6,3	5,0	6,4	7,0	5,9	7,0	5,5	5,7	6,0	6,1	6,0	6,0	4,0	6,2	5,0	5,0	5,8	*5,62*	1,00	17,82	
Hypokotyl length	2,3	2,5	2,8	2,7	2,5	3,0	2,1	2,7	3,3	2,2	2,0	2,7	3,2	2,5	2,0	1,5	2,5	2,0	2,5	2,5	2,48	0,43	17,18	
Control 2																								
Root length	5,3	5,8	5,7	5,8	3,6	2,8	4,0	6,1	5,0	5,9	2,7	4,4	5,1	5,9	5,2	5,5	5,5	4,5	4,6	5,2	*4,93*	0,98	19,79	
Hypokotyl length	2,8	3,0	2,1	2,4	2,3	2,4	1,7	3,0	2,7	2,5	1,5	2,5	2,3	2,3	2,4	1,9	3,5	2,2	1,7	1,8	*2,35*	0,49	20,65	
Control 3																								
Root length	6,2	6,0	6,3	5,4	7,0	5,5	6,5	5,0	5,2	5,0	5,5	6,0	4,5	5,0	5,0	5,2	4,9	5,3	5,8	5,5	*5,54*	0,61	11,07	
Hypokotyl length	2,9	3,0	2,5	2,7	2,8	2,0	3,0	2,7	2,5	2,7	2,5	2,3	2,5	3,0	2,5	2,0	2,5	2,8	2,7	2,5	2,61	0,28	10,75	
Control 4 filtreli																								
Root length	4,9	5,5	3,0	5,7	5,3	6,0	4,6	6,0	6,0	6,2	6,5	5,8	4,7	5,1	3,6	5,4	5,2	4,6	3,7	5,0	*5,14*	0,90	17,43	
Hypokotyl length	2,8	2,6	2,4	1,8	2,7	2,8	2,3	3,0	2,7	2,7	3,0	2,0	2,1	2,3	1,9	2,4	2,5	1,8	1,7	2,0	*2,4*	0,4	16,9	
Control 5																								
Root length	6,2	6,0	6,3	5,4	7,0	5,5	6,5	5,0	5,2	5,0	5,5	6,0	4,5	5,0	5,0	5,2	4,9	5,3	5,8	5,5	*5,54*	0,6	11,1	
Hypokotyl length	2,9	3,0	2,5	2,7	2,8	2,0	3,0	2,7	2,5	2,7	2,5	2,3	2,5	3,0	2,5	2,0	2,5	2,8	2,7	2,5	2,61	0,3	10,8	
Control 6																								
Root length	4,9	5,5	3,0	5,7	5,3	6,0	4,6	6,0	6,0	6,2	6,5	5,8	4,7	5,1	3,6	5,4	5,2	4,6	3,7	5,0	*5,14*	0,9	17,4	
Hypokotyl length	2,8	2,6	2,4	1,8	2,7	2,8	2,3	3,0	2,7	2,7	3,0	2,0	2,1	2,3	1,9	2,4	2,5	1,8	1,7	2,0	*2,4*	0,4	16,9	
				**C1**	**C2**	**C3**	**C4**	**C5**	**C6**															
Mean Root Length				*5,62*	*4,93*	*5,54*	*5,14*	*5,54*	*5,14*												***5,32***			
Mean Hypokotyl Length				2,48	2,35	2,61	2,38	2,61	2,38												**2,46**			
**conc**	100,00%																							
Sample 1																					**MW [cm]**	**STD DEV [cm]**	**VAR KOE**	**Inhibition [%]**
Root length	5,3	5,3	5,7	6,0	6,1	4,5	4,5	5,9	4,0	5,1	3,1	2,3	5,2	4,4	4,0	4,7	4,5	4,5	3,3	4,5	*4,65*	0,97	20,82	
Hypokotyl length	2,5	2,0	2,4	2,9	2,5	2,5	2,5	2,4	1,8	1,8	1,6	2,2	2,3	1,6	2,3	1,5	1,5	2,5	1,4	2,5	*2,14*	0,43	20,27	
Sample 2																								
Root length	3,5	4,5	3,2	2,0	3,3	5,0	3,0	5,0	3,0	4,5	5,5	3,6	4,6	5,0	5,0	4,0	4,3	3,0	3,5	4,0	*3,98*	0,89	22,49	
Hypokotyl length	2,0	2,1	2,0	1,5	2,0	2,0	2,1	1,5	2,2	3,0	2,0	2,0	2,0	2,4	2,0	2,5	1,5	2,0	2,0	2,0	*2,04*	0,33	16,21	
Sample 3																								
Root length	6,5	5,4	5,5	5,5	4,5	4,6	5,5	6,5	3,4	5,0	4,5	3,5	3,4	3,5	5,5	4,5	3,5	2,5	2,5	5,0	*4,54*	1,16	25,45	
Hypokotyl length	2,0	2,3	2,0	3,5	2,8	2,5	2,0	3,0	2,0	2,2	2,0	2,0	2,0	1,5	2,0	2,5	2,0	2,0	2,0	2,5	*2,24*	0,44	19,63	
				**S1**	**S2**	**S3**																		
Mean Root Length				*4,65*	*3,98*	*4,54*															**4,39**			**17,52**
Mean Hypokotyl Length				2,14	2,04	2,24															**2,14**			**13,22**
	50%																							
Sample 1																								
Root length	5,5	4,6	5,3	4,2	4,6	5,0	5,0	3,7	3,1	4,6	4,2	3,8	5,0	5,0	3,2	5,0	5,0	3,6	4,9	3,7	*4,5*	0,7	15,6	
Hypokotyl length	2,5	1,5	2,0	2,5	2,0	1,6	1,6	2,2	1,6	2,0	2,0	2,0	1,6	1,6	2,0	1,6	1,6	2,0	2,0	2,2	1,91	0,30	15,61	
Sample 2																								
Root length	3,1	5,6	5,1	3,7	5,9	4,6	5,7	5,1	5,8	5,9	6,1	6,4	4,9	5,4	4,9	5,0	3,7	5,0	5,8	3,1	*5,04*	0,94	18,74	
Hypokotyl length	1,4	3,5	2,5	2,2	2,5	2,9	2,3	2,5	2,9	2,1	3,0	3,3	2,4	2,4	3,0	2,0	2,2	1,6	2,6	2,3	2,48	0,51	20,51	
Sample 3																								
Root length	3,7	5,8	5,4	4,1	4,8	4,6	3,4	3,7	4,2	5,0	5,0	3,9	3,7	4,2	5,0	3,7	5,0	3,7	3,9	4,7	*4,4*	0,7	15,2	
Hypokotyl length	2,2	2,2	2,5	2,4	2,4	2,6	2,0	2,2	2,4	2,0	1,6	2,0	2,2	2,0	3,0	2,0	2,0	2,2	2,1	1,8	2,19	0,30	13,61	
				**S1**	**S2**	**S3**																		
Mean Root Length				*4,45*	*5,04*	*4,38*															**4,62**			**13,10**
Mean Hypokotyl Length				1,91	2,48	2,19															**2,19**			**11,06**
	25%																							
Sample 1																					**MW [cm]**	**STD DEV [cm]**	**VAR KOE**	**Inhibition [%]**
Root length	4,4	6,3	5,8	2,6	6,1	4,2	5,1	5,5	4,2	3,7	7,4	4,3	4,6	4,2	5,3	4,2	5,6	5,1	4,4	2,7	*4,79*	1,14	23,72	
Hypokotyl length	2,3	2,7	2,7	1,7	3,1	2,0	2,1	3,0	2,0	2,2	3,0	2,0	2,1	2,0	2,1	2,0	2,3	1,5	1,4	1,9	*2,21*	0,46	21,05	
Sample 2																								
Root length	3,8	4,8	5,5	6,1	6,4	5,9	6,5	4,2	6,1	7,5	5,0	5,1	3,6	4,7	5,3	5,5	4,5	4,2	4,7	2,5	*5,10*	1,13	22,17	
Hypokotyl length	2,2	2,3	2,3	3,2	3,6	3,0	3,3	1,4	2,0	3,0	2,8	1,9	3,0	2,0	1,8	2,2	1,2	1,4	2,3	2,1	*2,35*	0,66	27,98	
Sample 3																								
Root length	5,3	6,3	6,1	5,0	6,1	3,7	5,5	5,4	4,2	6,0	3,8	3,6	5,7	5,3	3,6	6,0	4,2	3,6	2,2	2,1	*4,69*	1,26	26,89	
Hypokotyl length	2,1	2,0	2,4	1,7	2,3	1,9	2,9	2,0	2,0	2,7	2,0	2,3	2,3	2,0	2,4	2,4	1,4	1,6	2,3	1,4	*2,11*	0,38	18,12	
				**S1**	**S2**	**S3**																		
Mean Root Length				*4,79*	*5,10*	*4,69*															***4,86***			**8,71**
Mean Hypokotyl Length				2,21	2,35	2,11															**2,22**			**9,91**

Table S3. Continued

	12,50%																							
Sample 1																								
Root length	5,5	6,0	4,5	3,5	3,7	5,5	5,0	4,1	6,0	3,5	5,0	4,5	3,5	6,4	2,5	5,0	4,5	5,5	3,8	2,5	*4,53*	1,10	24,28	
Hypokotyl length	2,0	3,0	2,8	2,5	2,0	2,7	2,0	2,0	2,0	2,0	2,5	2,5	2,0	2,5	1,5	2,0	2,0	2,5	2,3	2,0	*2,24*	0,36	16,00	
Sample 2																								
Root length	5,5	4,6	5,5	3,5	6,5	5,8	6,0	6,5	4,9	3,5	5,5	5,7	6,0	6,0	6,0	7,0	5,5	5,5	4,5	6,7	*5,54*	0,92	16,64	
Hypokotyl length	3,0	2,7	2,0	2,5	3,8	3,0	3,4	3,0	3,3	2,0	3,0	3,0	3,0	2,0	2,0	2,0	2,5	1,8	1,5	3,5	*2,65*	0,63	23,82	
Sample 3																								
Root length	4,0	5,3	7,3	3,5	4,5	3,5	5,5	4,0	5,5	4,5	4,7	6,0	5,0	4,0	4,7	5,5	3,5	3,4	5,0	5,0	*4,72*	0,96	20,41	
Hypokotyl length	2,0	2,0	2,5	2,3	2,0	2,2	2,0	2,0	1,5	2,0	2,0	1,8	1,5	2,0	2,0	1,5	1,0	2,0	2,0	1,5	*1,89*	0,33	17,46	
				**S1**	**S2**	**S3**																		
Mean Root Length				*4,53*	*5,54*	*4,72*															***4,93***			**7,36**
Mean Hypokotyl Length				2,24	2,65	1,89															**2,26**			**8,29**
	6,25%																							
Sample 1																					**MW [cm]**	**STD DEV [cm]**	**VAR KOE**	**Inhibition [%]**
Root length	5,0	4,3	5,7	5,8	6,0	6,2	5,5	6,0	4,0	4,2	3,5	5,0	6,0	5,5	5,8	5,0	4,3	4,0	4,2	3,0	*4,95*	0,93	18,72	
Hypokotyl length	2,5	3,0	2,0	2,5	3,0	3,0	2,0	2,0	2,3	2,3	2,0	2,5	2,0	2,0	2,5	2,0	2,0	2,0	2,3	2,0	2,30	0,36	15,50	
Sample 2																								
Root length	6,5	5,4	5,7	4,2	3,5	6,0	4,5	7,0	4,1	5,5	4,2	5,2	3,0	4,7	4,6	4,5	4,6	5,5	5,8	4,7	*4,96*	0,96	19,33	
Hypokotyl length	3,2	3,5	3,0	2,0	2,0	2,3	2,0	2,5	2,0	2,5	2,0	2,5	2,0	2,3	2,5	2,0	3,0	2,5	2,7	2,0	*2,43*	0,45	18,44	
Sample 3																								
Root length	4,2	4,9	4,9	5,1	4,7	6,1	5,1	4,9	4,2	4,9	5,4	5,6	6,0	4,2	5,1	4,8	4,2	4,2	4,5	5,8	*4,94*	0,59	11,93	
Hypokotyl length	2,3	2,3	3,1	1,8	2,4	3,0	2,0	2,2	2,3	2,7	2,3	2,0	2,5	2,3	2,0	2,0	2,3	2,3	2,0	2,0	*2,29*	0,33	14,28	
				**S1**	**S2**	**S3**																		
Mean Root Length				*4,95*	*4,96*	*4,94*															***4,95***			***6,93***
Mean Hypokotyl Length				2,30	2,43	2,29															**2,34**			***5,17***

Table S3. Continued

BA_{TSP}-Organic eluat

**LEPIDIUM SATIVUM**																								
Seed Charge:																								
Date:	16.05.2023																							
Name:	Ali ÇANKAYA																							
Sample Name:	Kampüs Tsp organik																							
Control:	3 kontrol 8 ml yerli																							
**KONTROLLEN**																								
Control 1	1	2	3	4	5	6	7	8	9	10	11	12	13	14	15	16	17	18	19	20	**MV [cm]**	**STD DEV [cm]**	**VAR KOE**	
Root length	7,3	8,7	5,3	7,4	8,2	7,5	6,5	7,5	7,7	7,4	6,7	5,9	6,7	9,4	5,9	7,9	7,3	8,3	7,8	6,0	*7,27*	1,00	13,76	
Hypokotyl length	3,7	3,8	3,5	3,4	3,7	3,3	3,4	4,0	3,5	3,5	3,4	3,6	2,8	4,3	3,3	3,0	3,4	3,9	3,2	3,6	3,52	0,33	9,40	
Control 2																								
Root length	7,5	5,2	7,8	5,5	8,3	7,8	6,0	7,5	6,9	6,9	8,0	6,9	6,1	8,1	6,0	8,0	6,4	5,6	7,0	7,6	*6,96*	0,94	13,51	
Hypokotyl length	2,6	2,2	3,5	2,9	3,3	3,3	3,3	3,0	3,2	3,0	3,2	3,2	3,2	3,0	3,2	2,9	3,4	2,4	3,0	3,5	*3,07*	0,33	10,88	
Control 3																								
Root length	6,8	7,0	6,1	7,1	8,7	7,6	8,7	6,2	6,4	8,2	7,7	7,4	7,8	6,0	6,9	5,8	6,7	6,9	7,2	6,7	*7,10*	0,81	11,46	
Hypokotyl length	4,0	3,5	3,1	3,6	3,6	3,5	3,1	3,5	3,4	3,5	3,4	1,0	3,7	3,3	2,9	3,3	3,2	3,6	2,6	2,5	3,22	0,62	19,21	
Control 4																								
Root length	7,3	8,7	5,3	7,4	8,2	7,5	6,5	7,5	7,7	7,4	6,7	5,9	6,7	9,4	5,9	7,9	7,3	8,3	7,8	6,0	*7,27*	1,00	13,76	
Hypokotyl length	3,7	3,8	3,5	3,4	3,7	3,3	3,4	4,0	3,5	3,5	3,4	3,6	2,8	4,3	3,3	3,0	3,4	3,9	3,2	3,6	*3,5*	0,3	9,4	
Control 5																								
Root length	7,5	5,2	7,8	5,5	8,3	7,8	6,0	7,5	6,9	6,9	6,0	6,9	6,1	8,1	6,0	8,0	9,0	5,6	7,0	7,6	*7,0*	1,0	14,8	
Hypokotyl length	2,6	2,2	3,5	2,9	3,3	3,3	3,3	3,0	3,2	3,0	3,3	3,2	3,2	3,0	3,2	2,9	3,3	2,4	3,0	3,5	*3,1*	0,3	10,8	
Control 6																								
Root length	6,8	7,0	6,1	7,1	8,7	7,6	8,7	6,2	6,4	6,0	6,4	7,4	6,4	9,4	6,9	5,8	6,7	6,9	7,2	6,7	7,0	0,9	13,3	
Hypokotyl length	4,0	3,5	3,1	3,6	3,6	3,5	3,1	3,5	3,4	3,3	3,4	1,0	3,4	3,1	2,9	3,3	3,2	3,6	2,6	2,5	3,2	0,6	19,0	
				**C1**	**C2**	**C3**	**C4**	**C5**	**C6**															
Mean Root Length				*7,27*	*6,96*	*7,10*	*7,27*	*6,99*	*7,02*												***7,11***			
Mean Hypokotyl Length				3,52	3,07	3,22	3,52	3,07	3,18												**3,27**			
**conc**	6.25%																							
Sample 1																					**MW [cm]**	**STD DEV [cm]**	**VAR KOE**	**Inhibition [%]**
Root length	6,5	7,1	8,1	7,2	7,8	7,1	7,8	6,3	7,8	8,0	7,9	5,6	7,1	6,3	7,8	5,6	5,1	7,1	7,8	7,8	*7,09*	0,88	12,43	
Hypokotyl length	3,6	4,0	4,0	4,2	3,1	3,6	3,1	3,9	3,1	2,6	4,0	3,7	3,9	2,8	4,2	3,0	3,0	4,3	3,1	3,1	*3,52*	0,52	14,71	
Sample 2																								
Root length	7,8	7,0	6,9	8,5	7,5	8,8	5,3	8,9	8,0	7,5	5,2	6,9	6,8	5,7	7,8	7,8	7,8	7,8	7,8	7,8	*7,38*	1,00	13,57	
Hypokotyl length	4,0	4,3	4,0	3,3	3,6	4,2	2,5	4,5	3,7	3,6	3,4	3,5	2,3	2,3	3,1	3,1	3,1	3,1	3,1	3,1	*3,39*	0,61	17,87	
Sample 3																								
Root length	5,9	7,2	6,7	6,1	7,0	5,8	7,5	6,6	6,5	8,0	7,8	7,8	7,6	9,2	5,6	6,0	6,5	7,8	7,8	7,0	*7,02*	0,90	12,83	
Hypokotyl length	2,9	3,6	3,8	3,0	4,3	3,5	3,6	3,2	3,5	3,2	3,1	3,1	3,4	4,2	3,3	2,5	3,3	3,1	3,1	3,3	*3,35*	0,41	12,22	
				**S1**	**S2**	**S3**																		
Mean Root Length				*7,09*	*7,38*	*7,02*															**7,16**			**-0,80**
Mean Hypokotyl Length				3,52	3,39	3,35															**3,42**			**-4,70**
	12,5																							
Sample 1																								
Root length	7,0	6,0	6,2	8,8	7,3	6,0	6,5	7,5	7,9	8,0	7,7	7,3	7,0	7,3	6,3	6,9	6,2	7,5	6,0	6,5	*7,0*	0,8	10,9	
Hypokotyl length	3,6	2,3	3,5	4,2	3,3	3,6	2,9	2,2	3,1	3,4	2,0	3,8	2,5	2,2	3,1	2,0	2,5	2,5	1,7	3,7	2,91	0,70	24,16	
Sample 2																								
Root length	6,5	8,2	6,1	7,6	8,2	5,6	7,7	6,0	7,5	6,0	7,0	6,3	6,9	7,2	7,0	6,9	7,5	7,1	7,0	7,5	*6,99*	0,71	10,15	
Hypokotyl length	3,8	4,0	3,8	3,3	3,0	4,2	3,3	3,8	4,5	4,0	3,9	2,7	3,2	3,1	3,0	3,1	3,9	3,9	4,0	3,6	3,61	0,47	13,02	
Sample 3																								
Root length	5,6	6,9	8,9	7,4	6,4	7,4	6,9	6,7	7,7	7,3	6,3	7,2	7,0	7,3	7,2	6,5	6,5	7,0	8,1	8,5	*7,1*	0,7	10,5	
Hypokotyl length	3,0	4,0	4,2	3,1	3,7	2,6	2,2	2,7	2,9	3,8	3,8	3,2	3,2	3,6	3,8	3,4	3,5	3,2	3,5	3,0	3,32	0,49	14,84	
				**S1**	**S2**	**S3**																		
Mean Root Length				*7,00*	*6,99*	*7,14*															**7,04**			**0,91**
Mean Hypokotyl Length				2,91	3,61	3,32															**3,28**			**-0,36**
	25%																							
Sample 1																					**MW [cm]**	**STD DEV [cm]**	**VAR KOE**	**Inhibition [%]**
Root length	8,4	9,1	7,2	8,2	7,2	7,4	7,4	7,1	6,1	7,6	8,6	6,5	7,2	6,3	6,3	5,9	5,5	5,6	6,9	7,3	*7,09*	0,96	13,59	
Hypokotyl length	4,1	4,1	4,3	3,9	4,0	3,9	4,0	4,1	3,2	4,3	4,1	4,2	3,1	3,8	3,6	2,9	1,8	3,2	3,5	3,1	*3,66*	0,61	16,58	
Sample 2																								
Root length	7,6	7,2	7,4	6,6	7,3	7,9	7,4	7,9	7,7	6,5	7,5	6,9	7,7	6,9	7,0	5,5	6,5	6,0	7,1	7,7	*7,12*	0,63	8,79	
Hypokotyl length	3,8	4,5	3,7	3,8	4,0	3,1	4,5	3,6	3,7	4,1	3,6	3,8	4,0	3,2	3,5	3,7	4,0	3,7	3,5	4,2	*3,80*	0,35	9,30	
Sample 3																								
Root length	7,6	7,2	6,8	6,7	7,3	7,3	7,0	7,0	7,3	9,1	6,5	5,5	6,2	6,9	5,0	8,6	6,4	7,5	5,4	7,1	*6,92*	0,95	13,70	
Hypokotyl length	4,0	3,9	3,8	4,0	3,8	3,8	3,3	3,6	3,7	4,2	3,8	3,4	3,3	3,7	3,2	4,0	3,8	4,6	2,9	4,0	*3,74*	0,37	9,98	
				**S1**	**S2**	**S3**																		
Mean Root Length				*7,09*	*7,12*	*6,92*															***7,04***			**0,91**
Mean Hypokotyl Length				3,66	3,80	3,74															**3,73**			**-14,34**

	50,0%																							
Sample 1																								
Root length	7,1	7,8	7,2	8,0	6,2	7,1	6,2	7,6	7,2	7,5	7,2	7,4	7,1	6,2	7,5	6,3	6,4	6,6	6,3	6,1	*6,95*	0,59	8,51	
Hypokotyl length	4,2	3,7	3,1	3,5	3,5	3,2	3,2	4,0	4,2	4,1	3,5	4,1	3,5	4,0	3,7	3,2	3,4	3,7	3,3	2,8	*3,60*	0,39	10,97	
Sample 2																								
Root length	9,6	6,3	6,1	8,3	6,0	6,1	7,2	6,7	7,2	7,1	8,1	6,2	7,0	8,0	7,2	7,1	6,5	6,5	5,9	5,0	*6,91*	1,00	14,54	
Hypokotyl length	4,5	3,2	3,7	3,5	3,3	3,6	3,6	4,1	3,1	4,0	3,9	3,2	3,9	4,7	4,2	3,1	3,5	2,9	4,2	3,1	*3,67*	0,50	13,54	
Sample 3																								
Root length	7,9	8,3	7,6	7,4	8,1	6,9	6,3	5,2	7,1	5,2	6,9	6,7	5,3	7,1	9,0	6,6	7,0	6,7	6,0	7,6	*6,95*	0,99	14,32	
Hypokotyl length	3,5	3,9	3,3	3,9	3,9	4,1	3,6	3,6	3,1	3,0	3,9	3,0	3,0	4,1	3,7	3,1	4,3	3,1	3,7	4,0	*3,59*	0,42	11,58	
				**S1**	**S2**	**S3**																		
Mean Root Length				*6,95*	*6,91*	*6,95*															***6,93***			**2,44**
Mean Hypokotyl Length				3,60	3,67	3,59															**3,62**			**-10,77**
	100%																							
Sample 1																					**MW [cm]**	**STD DEV [cm]**	**VAR KOE**	**Inhibition [%]**
Root length	6,0	7,2	5,6	6,6	6,4	6,9	7,2	8,0	8,3	7,6	7,1	7,5	5,8	6,5	6,0	4,6	6,2	4,4	7,2	4,8	*6,50*	1,06	16,33	
Hypokotyl length	3,8	4,5	3,0	4,3	3,6	3,1	4,8	4,8	3,9	4,3	4,0	4,0	3,3	4,0	3,6	3,8	3,2	3,7	4,6	3,1	3,87	0,55	14,13	
Sample 2																								
Root length	7,5	6,9	7,3	4,9	7,0	7,3	5,1	5,2	7,5	6,0	6,8	7,8	5,6	7,4	6,4	7,9	8,3	4,6	5,6	6,9	*6,60*	1,08	16,30	
Hypokotyl length	4,8	4,5	4,9	4,3	2,6	4,6	3,1	4,1	3,7	3,1	4,4	4,7	2,6	4,1	3,9	3,6	4,5	3,1	2,8	4,5	*3,90*	0,75	19,17	
Sample 3																								
Root length	9,6	6,8	7,9	6,6	6,1	5,7	6,9	5,1	7,6	6,7	5,9	6,2	6,6	3,9	5,5	8,0	7,0	4,6	6,6	5,5	*6,44*	1,25	19,37	
Hypokotyl length	4,2	4,5	3,2	4,1	3,1	4,3	3,8	3,1	4,0	4,2	4,0	4,2	3,8	3,7	3,8	3,9	3,9	2,9	2,5	3,7	*3,75*	0,51	13,63	
				**S1**	**S2**	**S3**																		
Mean Root Length				*6,50*	*6,60*	*6,44*															***6,51***			***8,37***
Mean Hypokotyl Length				3,87	3,90	3,75															**3,84**			***-17,51***

Table S3. Continued

BA$_{TSP}$-Inorganic eluat

**LEPIDIUM SATIVUM**																								
Seed Charge:																								
Date:																								
Name:																								
Sample Name:	Kampüs toz TSP-inorganik																							
Control:	8 mL seralpur n=6																							
**KONTROLLEN**																								
Control 1 rand	1	2	3	4	5	6	7	8	9	10	11	12	13	14	15	16	17	18	19	20	MV [cm]	STD DEV [cm]	VAR KOE	
Root length	5,9	4,0	3,1	4,5	5,1	4,0	5,2	4,5	4,0	3,8	3,0	3,0	3,0	3,0	3,0	4,5	3,5	3,5	3,5	3,5	*3,88*	0,82	21,23	
Hypokotyl length	3,2	3,0	2,9	2,1	2,5	2,9	3,0	3,2	3,5	1,4	1,4	1,4	1,4	1,4	1,4	3,2	1,8	1,8	1,8	1,8	2,26	0,76	33,58	
Control 2																								
Root length	4,5	4,0	4,0	5,0	3,9	3,2	3,5	3,5	3,5	5,1	3,5	3,5	3,5	3,5	3,5	3,5	4,0	4,0	4,0	4,0	*3,86*	0,50	12,91	
Hypokotyl length	2,3	0,2	3,8	2,6	3,3	2,1	1,8	2,7	1,8	2,8	1,8	1,8	1,8	1,8	1,8	1,8	3,8	3,8	3,8	3,8	*2,47*	0,96	39,07	
Control 3																								
Root length	4,4	4,2	2,6	4,9	3,9	3,6	3,3	3,5	3,5	5,0	3,5	2,4	2,3	3,3	2,6	3,3	3,3	3,3	3,3	3,3	*3,48*	0,71	20,57	
Hypokotyl length	2,8	2,7	2,5	2,5	3,7	2,5	2,4	2,2	2,4	2,2	1,7	1,1	1,4	2,4	1,1	2,4	2,4	2,4	2,4	2,4	2,28	0,58	25,26	
Control 4																								
Root length	5,9	4,0	3,1	4,5	5,1	4,0	5,2	4,5	4,0	3,8	3,0	3,0	3,0	3,0	3,0	4,5	3,5	3,5	3,5	3,5	*3,88*	0,82	21,23	
Hypokotyl length	3,2	3,0	2,9	2,1	2,5	2,9	3,0	3,2	3,5	1,4	1,4	1,4	1,4	1,4	1,4	3,2	1,8	1,8	1,8	1,8	*2,3*	0,8	33,6	
Control 5																								
Root length	4,5	4,0	4,0	5,0	3,9	3,2	3,5	3,5	3,5	5,1	3,5	3,5	3,5	3,5	3,5	3,5	4,0	4,0	4,0	4,0	*3,9*	0,5	12,9	
Hypokotyl length	2,3	0,2	3,8	2,6	3,3	2,1	1,8	2,7	1,8	2,8	1,8	1,8	1,8	1,8	1,8	1,8	3,8	3,8	3,8	3,8	*2,5*	1,0	39,1	
Control 6																								
Root length	4,4	4,2	2,6	4,9	3,9	3,6	3,3	3,5	3,5	5,0	3,5	2,4	2,3	3,3	2,6	3,3	3,3	3,3	3,3	3,3	3,5	0,7	20,6	
Hypokotyl length	2,8	2,7	2,5	2,5	3,7	2,5	2,4	2,2	2,4	2,2	1,7	1,1	1,4	2,4	1,1	2,4	2,4	2,4	2,4	2,4	2,3	0,6	25,3	
				**C1**	**C2**	**C3**	**C4**	**C5**	**C6**															
Mean Root Length				*3,88*	*3,86*	*3,48*	*3,88*	*3,86*	*3,48*												***3,74***			
Mean Hypokotyl Length				2,26	2,47	2,28	2,26	2,47	2,28												**2,34**			
**conc**	6,25%																							
Sample 1 rand																					MW [cm]	STD DEV [cm]	VAR KOE	Inhibition [%]
Root length	2,5	3,6	1,5	3,1	3,3	4,3	2,8	2,8	4,0	4,1	2,8	2,7	2,9	2,7	2,8	4,3	2,8	4,3	2,8	2,7	*3,14*	0,72	22,93	
Hypokotyl length	0,6	2,7	2,5	3,3	2,2	3,2	2,0	2,0	2,8	2,5	2,0	2,1	2,0	2,1	2,0	3,2	2,0	3,2	2,0	2,1	*2,33*	0,61	26,44	
Sample 2																								
Root length	1,9	2,7	2,2	4,7	3,2	2,6	2,0	2,7	2,7	2,7	2,9	2,6	2,0	2,7	2,9	2,7	2,7	4,3	2,8	2,7	*2,79*	0,66	23,61	
Hypokotyl length	2,7	2,5	2,0	2,4	2,3	2,1	1,6	2,1	2,1	2,1	2,0	2,1	1,6	2,1	2,0	2,1	2,1	3,2	2,0	2,1	*2,16*	0,34	15,80	
Sample 3																								
Root length	3,6	4,5	5,5	4,6	3,6	2,9	5,2	2,7	3,0	5,1	3,6	2,9	5,2	2,7	2,7	2,7	4,3	2,8	4,3	2,8	*3,74*	0,98	26,28	
Hypokotyl length	3,0	2,5	3,7	2,9	1,7	2,0	2,8	2,1	3,5	2,9	1,7	2,0	2,8	2,1	2,1	2,1	3,2	2,0	3,2	2,0	*2,52*	0,60	23,67	
				**S1**	**S2**	**S3**																		
Mean Root Length				*3,14*	*2,79*	*3,74*															**3,22**			**13,87**
Mean Hypokotyl Length				2,33	2,16	2,52															**2,33**			**0,07**
	12.5%																							
Sample 1 saide																								
Root length	2,3	3,1	3,8	3,8	2,9	2,3	3,8	3,8	3,8	4,3	4,3	2,3	2,3	3,1	3,8	2,3	2,9	3,8	3,8	2,3	*3,2*	0,7	22,2	
Hypokotyl length	3,0	2,2	3,1	3,1	2,3	1,7	2,1	2,1	3,1	2,2	2,2	1,7	3,0	2,2	3,1	1,9	2,3	3,1	2,1	1,7	2,41	0,52	21,44	
Sample 2																								
Root length	2,1	3,4	4,5	2,4	2,1	4,6	3,8	3,8	3,8	3,3	3,6	3,3	3,8	3,4	4,5	2,4	3,8	4,6	2,1	4,6	*3,50*	0,85	24,35	
Hypokotyl length	2,2	1,7	2,7	2,6	1,7	1,9	3,1	2,1	2,1	2,2	2,6	3,8	2,1	1,7	2,7	2,6	2,1	1,9	1,7	1,9	2,27	0,53	23,23	
Sample 3																								
Root length	3,8	3,8	3,0	3,8	3,8	3,8	3,7	3,3	4,3	3,8	3,8	3,3	4,3	3,8	3,8	3,8	3,8	3,8	3,8	0,4	*3,6*	0,8	21,9	
Hypokotyl length	2,1	2,1	2,5	2,1	3,1	3,1	2,3	2,1	2,2	2,1	2,1	2,1	2,2	2,1	2,1	2,1	2,1	2,1	2,1	0,3	2,15	0,52	24,19	
				**S1**	**S2**	**S3**																		
Mean Root Length				*3,24*	*3,50*	*3,59*															**3,44**			**7,98**
Mean Hypokotyl Length				2,41	2,27	2,15															**2,28**			**2,50**
	25%																							
Sample 1 saide																					MW [cm]	STD DEV [cm]	VAR KOE	Inhibition [%]
Root length	3,5	2,3	2,5	3,2	3,6	3,5	3,5	4,4	3,5	2,3	3,5	2,3	3,5	3,2	3,6	3,5	3,5	4,4	3,5	4,4	*3,39*	0,62	18,31	
Hypokotyl length	2,5	1,5	2,9	3,1	2,1	2,5	2,5	2,9	1,8	1,5	2,5	1,5	2,5	3,1	2,1	2,5	2,5	2,9	2,5	2,9	*2,42*	0,50	20,76	
Sample 2																								
Root length	3,9	3,0	2,9	3,6	3,5	3,5	3,5	3,5	4,4	3,4	3,9	3,0	3,5	3,6	3,9	3,0	4,4	3,6	3,4	3,4	*3,55*	0,40	11,34	
Hypokotyl length	2,3	2,3	1,9	1,9	2,5	2,5	2,5	2,5	2,9	1,9	2,3	2,3	2,5	1,9	2,3	2,3	2,9	1,9	2,4	1,9	*2,30*	0,31	13,39	
Sample 3																								
Root length	3,5	3,5	3,4	5,0	3,9	3,0	3,5	3,6	3,5	3,5	3,0	3,5	3,5	3,5	3,5	4,4	3,5	3,0	3,0	3,0	*3,52*	0,47	13,47	
Hypokotyl length	2,5	2,5	2,3	2,5	2,3	2,3	2,5	1,9	2,5	2,5	2,1	2,5	2,5	2,5	2,5	2,9	2,5	2,1	2,1	2,1	*2,38*	0,22	9,36	
				**S1**	**S2**	**S3**																		
Mean Root Length				*3,39*	*3,55*	*3,52*															***3,48***			**6,87**
Mean Hypokotyl Length				2,42	2,30	2,38															**2,36**			**-1,21**

Table S3. Continued

	50,0%																							
Sample 1 rand																								
Root length	2,5	3,1	3,1	3,1	3,5	3,3	3,1	3,1	2,3	2,5	4,4	4,4	4,4	4,4	4,4	4,4	3,8	4,4	4,2	4,4	*3,64*	0,74	20,33	
Hypokotyl length	2,1	2,4	2,2	2,4	1,7	2,5	2,4	2,4	1,8	1,8	3,1	2,2	2,2	2,2	2,2	2,2	2,0	2,2	1,6	2,2	*2,19*	0,32	14,58	
Sample 2																								
Root length	3,6	3,1	5,0	3,1	3,1	4,7	4,2	3,1	3,5	3,1	3,8	3,1	3,5	2,4	3,8	3,8	3,1	3,5	2,4	3,8	*3,49*	0,64	18,24	
Hypokotyl length	3,2	2,4	2,7	2,4	2,4	3,0	2,6	2,4	2,2	2,4	2,3	2,4	2,2	0,8	2,3	2,3	2,4	2,2	0,8	2,3	*2,29*	0,55	24,26	
Sample 3																								
Root length	4,0	3,1	4,7	3,8	4,4	4,2	4,1	3,4	3,4	3,2	3,4	3,4	3,4	4,1	4,0	3,1	4,7	3,8	4,4	4,2	*3,84*	0,50	13,06	
Hypokotyl length	2,4	0,9	2,3	2,0	2,2	1,6	2,3	1,3	1,3	2,9	1,3	1,3	1,3	2,8	2,4	0,9	2,3	2,0	2,2	1,6	*1,87*	0,59	31,60	
				**S1**	**S2**	**S3**																		
Mean Root Length				*3,64*	*3,49*	*3,84*															***3,66***			**2,23**
Mean Hypokotyl Length				2,19	2,29	1,87															**2,11**			**9,49**
	100%																							
Sample 1 saide																					**MW [cm]**	**STD DEV [cm]**	**VAR KOE**	**Inhibition [%]**
Root length	4,6	3,0	3,0	3,5	5,7	3,6	3,0	3,0	3,0	3,0	2,2	3,0	4,6	3,0	3,0	3,5	5,7	3,6	5,7	3,6	*3,67*	1,01	27,53	
Hypokotyl length	4,0	3,1	3,1	2,2	2,6	1,4	3,1	3,1	3,1	3,1	0,9	3,1	4,0	3,1	3,1	2,2	2,6	1,4	2,6	1,4	2,66	0,83	31,03	
Sample 2 rand																								
Root length	5,3	3,7	3,0	5,5	3,6	2,3	3,8	4,0	3,5	2,8	2,7	4,2	5,3	3,7	3,0	5,5	3,6	2,3	3,8	3,8	*3,77*	0,97	25,61	
Hypokotyl length	3,9	2,1	3,3	2,1	2,9	2,2	1,7	2,3	2,3	1,5	2,1	2,7	3,9	2,1	3,3	2,1	2,9	2,2	1,7	1,7	*2,45*	0,69	28,12	
Sample 3 rand																								
Root length	2,6	4,6	3,1	2,4	1,6	4,1	1,9	2,7	2,7	2,7	2,6	4,6	3,1	2,4	1,6	4,1	1,9	2,7	1,3	1,6	*2,72*	0,96	35,45	
Hypokotyl length	3,0	2,3	2,5	2,1	0,6	2,0	0,5	2,1	2,1	2,1	3,0	2,3	2,5	2,1	0,6	2,0	0,5	2,1	0,4	0,6	*1,77*	0,86	48,31	
				**S1**	**S2**	**S3**																		
Mean Root Length				*3,67*	*3,77*	*2,72*															***3,38***			***9,50***
Mean Hypokotyl Length				2,66	2,45	1,77															**2,29**			***1,78***

Table S3. Continued

MQA$_{TD}$-Inorganic eluat

**LEPIDIUM SATIVUM**																								
Seed Charge:																								
Date:	16.05.2023																							
Name:	Ali ÇANKAYA																							
Sample Name:	Karaömerler Taş Ocağı Çözünmüş																							
Control:	3 kontrol yerli filtre 8 ml																							
**KONTROLLEN**																								
Control 1	1	2	3	4	5	6	7	8	9	10	11	12	13	14	15	16	17	18	19	20	**MV [cm]**	**STD DEV [cm]**	**VAR KOE**	
Root length	8,0	6,0	6,9	5,2	5,6	5,7	5,4	5,4	6,9	5,6	5,4	5,5	5,4	5,5	6,2	5,4	5,4	5,4	6,3	5,4	*5,83*	0,69	11,88	
Hypokotyl length	3,0	3,0	3,0	2,8	2,8	3,0	3,2	3,2	2,5	3,1	3,2	3,0	3,2	2,7	2,4	3,2	3,2	3,2	2,8	3,2	2,99	0,24	8,02	
Control 2																								
Root length	5,3	6,3	6,2	6,5	6,0	5,8	5,9	6,0	6,2	5,2	7,3	5,2	5,5	6,5	5,6	5,5	5,4	5,4	5,5	5,4	*5,84*	0,53	9,09	
Hypokotyl length	3,4	2,5	2,7	3,5	3,0	2,7	4,5	2,0	2,6	2,6	3,4	2,5	2,7	2,8	3,2	2,5	3,2	3,2	2,2	3,2	*2,92*	0,54	18,56	
Control 3																								
Root length	7,0	5,4	6,3	5,4	7,0	5,9	7,2	7,8	4,6	5,7	7,3	5,5	5,7	5,4	5,7	5,5	5,5	5,4	7,3	6,8	*6,12*	0,86	14,12	
Hypokotyl length	3,6	2,6	3,3	3,0	2,9	3,2	3,0	3,4	2,7	3,0	2,7	3,3	3,0	3,2	2,9	3,3	3,3	3,2	2,4	2,9	3,05	0,29	9,55	
Control 4																								
Root length	8,0	6,0	6,9	5,2	5,6	5,7	5,4	5,4	6,9	5,6	5,0	5,5	5,4	5,5	6,2	5,4	5,0	5,4	6,3	5,4	*5,79*	0,73	12,56	
Hypokotyl length	3,0	3,0	3,0	2,8	2,8	3,0	3,2	3,2	2,5	3,1	2,9	3,0	3,2	2,7	2,4	3,2	2,7	3,2	2,8	3,2	*2,9*	0,2	8,0	
Control 5																								
Root length	5,3	6,3	6,2	6,5	6,0	5,8	5,9	6,0	6,2	5,2	7,3	5,2	5,5	6,5	5,6	5,5	5,5	5,5	5,5	5,5	*5,9*	0,5	8,9	
Hypokotyl length	3,4	2,5	2,7	3,5	3,0	2,7	4,5	2,0	2,6	2,6	3,4	2,5	2,7	2,8	3,2	2,5	3,3	3,3	2,2	3,3	*2,9*	0,6	18,8	
Control 6																								
Root length	7,0	5,4	6,3	5,4	7,0	5,9	7,2	7,8	4,6	5,7	7,3	6,8	5,7	4,9	5,7	6,2	5,5	4,9	7,3	6,8	6,2	0,9	14,7	
Hypokotyl length	3,6	2,6	3,3	3,0	2,9	3,2	3,0	3,4	2,7	3,0	2,7	2,7	3,0	3,1	2,9	2,7	3,3	2,4	2,4	2,9	2,9	0,3	10,6	
				**C1**	**C2**	**C3**	**C4**	**C5**	**C6**															
Mean Root Length				*5,83*	*5,84*	*6,12*	*5,79*	*5,85*	*6,17*												***5,93***			
Mean Hypokotyl Length				2,99	2,92	3,05	2,95	2,94	2,94												**2,98**			
**conc**	6.25%																							
Sample 1																					**MW [cm]**	**STD DEV [cm]**	**VAR KOE**	**Inhibition [%]**
Root length	6,0	8,1	6,6	5,1	6,5	6,9	5,6	6,6	6,2	6,2	6,9	5,9	6,9	6,6	6,5	6,0	5,9	4,9	5,6	5,0	*6,20*	0,74	11,99	
Hypokotyl length	3,0	3,5	3,0	2,8	2,7	2,5	3,2	2,3	2,3	3,3	3,1	3,1	3,2	3,2	3,0	3,2	3,0	2,6	2,6	2,7	*2,92*	0,33	11,24	
Sample 2																								
Root length	6,4	6,2	6,7	5,2	5,9	5,6	5,6	6,5	6,0	6,0	6,6	6,0	5,2	6,3	5,0	6,0	5,6	5,5	5,1	5,1	*5,83*	0,52	8,93	
Hypokotyl length	3,7	3,2	3,1	2,7	3,8	3,3	3,4	3,1	3,3	3,6	3,7	2,9	2,3	3,2	3,4	3,5	3,1	2,9	2,4	3,0	*3,18*	0,40	12,48	
Sample 3																								
Root length	6,0	6,2	6,1	6,4	6,1	5,5	4,7	6,0	5,5	6,1	6,0	6,3	6,2	6,1	5,8	5,2	6,1	4,7	4,8	5,6	*5,77*	0,52	9,07	
Hypokotyl length	3,7	3,2	3,3	3,2	2,4	2,5	2,7	2,9	3,4	2,9	2,5	3,0	2,7	2,7	3,1	1,5	2,8	2,8	3,0	2,7	*2,85*	0,44	15,59	
				**S1**	**S2**	**S3**																		
Mean Root Length				*6,20*	*5,83*	*5,77*															**5,93**			**-0,06**
Mean Hypokotyl Length				2,92	3,18	2,85															**2,98**			**0,06**
	12,5																							
Sample 1																								
Root length	5,5	5,7	6,3	5,9	6,6	8,5	6,4	6,1	8,3	6,7	4,3	6,1	6,8	6,9	6,1	6,9	6,9	7,2	6,8	6,7	*6,1*	0,9	14,6	
Hypokotyl length	3,1	3,9	3,5	4,2	3,6	4,0	3,7	3,2	3,8	3,2	3,1	3,0	3,2	4,2	4,0	3,7	3,9	2,8	3,2	3,2	3,53	0,42	11,83	
Sample 2																								
Root length	6,2	6,7	6,0	6,5	6,1	6,2	5,1	4,3	5,4	4,5	6,2	5,4	5,6	6,6	6,1	6,0	6,9	5,2	6,5	3,3	*5,74*	0,89	15,44	
Hypokotyl length	4,5	4,2	3,4	3,4	3,6	3,1	3,2	3,1	4,0	3,1	3,5	3,1	2,8	2,6	3,3	3,3	3,4	2,5	2,6	3,0	3,29	0,50	15,26	
Sample 3																								
Root length	6,7	4,3	5,9	5,4	6,3	6,1	5,2	6,6	6,4	6,5	6,3	6,1	6,7	6,0	6,7	4,8	6,9	6,7	5,0	4,3	*5,9*	0,8	13,5	
Hypokotyl length	4,4	3,1	2,9	3,8	4,7	3,7	3,4	3,2	3,9	3,9	2,7	4,4	3,9	2,9	2,7	3,5	3,6	4,0	1,7	3,1	3,48	0,69	19,91	
				**S1**	**S2**	**S3**																		
Mean Root Length				*6,10*	*5,74*	*5,95*															**5,93**			**0,00**
Mean Hypokotyl Length				3,53	3,29	3,48															**3,43**			**-14,92**
	25%																							
Sample 1																					**MW [cm]**	**STD DEV [cm]**	**VAR KOE**	**Inhibition [%]**
Root length	7,1	6,1	4,8	5,5	4,3	4,8	4,4	4,8	3,9	5,6	3,6	5,4	5,2	4,8	5,4	5,3	5,1	4,7	3,2	4,5	*4,93*	0,85	17,19	
Hypokotyl length	3,9	2,6	3,2	3,1	2,6	3,4	3,1	2,9	3,6	3,0	2,4	3,5	2,5	2,2	2,8	2,6	2,1	2,4	2,2	2,5	*2,83*	0,50	17,49	
Sample 2																								
Root length	5,3	6,0	6,2	6,5	6,8	7,8	6,0	7,5	6,5	6,6	6,4	6,2	7,5	6,0	5,1	6,1	5,4	6,1	5,8	6,1	*6,30*	0,69	10,93	
Hypokotyl length	3,8	3,7	4,1	4,2	3,6	3,6	3,5	2,7	3,0	3,8	3,2	3,2	3,8	4,2	2,8	4,3	3,4	3,6	4,2	3,2	*3,60*	0,46	12,88	
Sample 3																								
Root length	8,5	7,2	6,7	6,7	6,7	6,1	5,1	5,8	6,7	7,5	6,5	7,3	5,9	5,8	6,7	6,3	6,6	6,2	6,4	5,2	*6,50*	0,76	11,74	
Hypokotyl length	4,2	4,2	4,5	3,7	4,6	3,6	3,9	4,5	3,9	4,1	4,0	4,2	3,7	3,0	4,1	4,4	3,8	4,1	4,1	4,2	*4,04*	0,36	8,90	
				**S1**	**S2**	**S3**																		
Mean Root Length				*4,93*	*6,30*	*6,50*															***5,91***			**0,39**
Mean Hypokotyl Length				2,83	3,60	4,04															**3,49**			**-16,93**

Table S3. Continued

	50,0%																							
Sample 1																								
Root length	5,0	4,2	8,2	6,1	6,6	4,9	5,5	6,2	6,0	5,9	6,2	6,5	6,7	6,1	5,6	4,6	5,7	4,2	4,5	5,4	*5,71*	0,95	16,66	
Hypokotyl length	4,4	4,5	4,7	4,6	3,2	3,2	3,7	2,9	4,3	2,6	4,3	4,2	4,6	4,9	3,2	2,6	3,8	3,9	4,1	2,2	*3,80*	0,78	20,62	
Sample 2																								
Root length	5,7	5,7	6,0	4,2	5,9	5,1	6,6	6,2	5,9	5,2	5,6	6,1	5,9	6,4	7,0	5,6	5,0	6,7	5,6	5,3	*5,79*	0,63	10,94	
Hypokotyl length	2,9	3,0	4,2	3,2	3,6	4,1	3,7	3,1	3,2	3,6	3,6	4,0	2,5	3,5	3,2	3,1	3,9	4,5	3,3	3,5	*3,49*	0,48	13,71	
Sample 3																								
Root length	6,7	7,2	5,9	5,2	5,0	6,0	4,7	7,2	5,3	6,4	6,7	5,2	5,9	5,9	5,2	5,5	4,5	6,4	7,6	5,2	*5,89*	0,86	14,59	
Hypokotyl length	4,4	3,8	4,6	4,8	3,0	3,0	2,8	3,7	3,8	5,1	3,6	4,0	3,6	3,6	2,7	3,6	3,5	3,7	5,0	3,1	*3,77*	0,69	18,19	
				**S1**	**S2**	**S3**																		
Mean Root Length				*5,71*	*5,79*	*5,89*															***5,79***			**2,31**
Mean Hypokotyl Length				3,80	3,49	3,77															**3,68**			**-23,46**
	100%																							
Sample 1																					**MW [cm]**	**STD DEV [cm]**	**VAR KOE**	**Inhibition [%]**
Root length	5,5	4,5	7,7	7,4	4,9	6,5	5,9	6,7	4,8	5,8	5,9	5,7	5,5	6,8	5,3	4,5	6,1	4,6	4,2	6,8	*5,76*	0,98	17,01	
Hypokotyl length	4,0	5,0	4,0	4,1	3,5	3,7	3,6	3,9	3,6	2,9	3,6	3,5	3,7	3,7	2,8	2,6	3,0	3,1	3,6	2,8	3,54	0,55	15,45	
Sample 2																								
Root length	6,4	6,3	5,3	5,5	5,1	6,2	5,5	6,3	5,5	5,2	8,4	6,3	5,6	5,7	6,7	4,0	5,4	6,0	7,0	2,8	*5,76*	1,10	19,06	
Hypokotyl length	4,4	3,0	4,2	3,4	4,7	4,7	4,0	3,3	3,9	3,8	3,5	4,4	3,6	5,2	4,2	3,2	2,7	4,3	2,7	2,7	*3,80*	0,71	18,72	
Sample 3																								
Root length	6,2	8,0	6,4	6,0	6,2	5,2	7,4	5,5	4,9	6,6	5,7	6,6	5,8	6,5	6,6	5,9	4,2	4,5	4,0	4,1	*5,82*	1,05	18,11	
Hypokotyl length	5,1	3,6	3,9	4,5	4,3	4,7	5,0	3,9	3,9	3,6	4,5	3,8	3,9	3,8	4,3	3,2	2,8	2,9	3,6	2,3	*3,88*	0,70	18,11	
				**S1**	**S2**	**S3**																		
Mean Root Length				*5,76*	*5,76*	*5,82*															***5,78***			***2,56***
Mean Hypokotyl Length				3,54	3,80	3,88															**3,74**			***-25,25***

Table S3. Continued

MQA_{TD}-Organic eluat

**LEPIDIUM SATIVUM**																								
Seed Charge:																								
Date:	17.04.2023																							
Name:																								
Sample Name:	Köyiçi organik																							
Control:																								
**KONTROLLEN**																								
Control 1	1	2	3	4	5	6	7	8	9	10	11	12	13	14	15	16	17	18	19	20	**MV [cm]**	**STD DEV [cm]**	**VAR KOE**	
Root length	6,0	6,1	6,5	6,4	6,5	5,9	6,6	5,9	6,7	6,2	5,1	6,3	6,0	5,5	4,9	6,7	5,4	4,9	6,1	4,9	*5,93*	0,60	10,06	
Hypokotyl length	2,3	3,4	2,3	2,8	3,2	3,1	3,4	2,8	3,0	3,1	2,0	2,4	3,0	2,0	2,5	2,7	2,9	3,1	2,1	2,3	2,72	0,44	16,22	
Control 2																								
Root length	7,0	6,0	6,3	7,3	6,9	6,6	7,2	6,7	6,2	5,5	7,0	6,1	5,9	6,0	5,9	5,8	5,4	5,5	5,5	6,0	*6,24*	0,59	9,45	
Hypokotyl length	2,6	3,0	2,9	2,2	2,8	2,1	3,3	3,0	2,9	3,1	3,2	3,3	2,4	2,7	2,7	2,8	2,2	2,4	2,6	2,4	*2,73*	0,36	13,06	
Control 3																								
Root length	6,0	9,0	6,4	7,6	8,5	6,5	6,3	6,3	5,5	5,8	6,8	6,2	6,5	6,9	6,2	6,4	6,0	5,3	4,8	5,4	*6,42*	0,98	15,34	
Hypokotyl length	3,2	3,1	2,8	1,4	3,1	3,3	3,3	2,9	2,5	3,4	2,2	3,5	3,5	3,3	2,9	3,6	2,8	2,8	2,0	2,1	2,89	0,57	19,74	
Control 4																								
Root length	6,4	7,5	6,6	6,3	5,8	7,4	7,7	7,2	7,5	6,8	7,3	5,7	6,0	7,1	6,0	7,6	6,7	7,7	6,8	6,4	*6,83*	0,64	9,38	
Hypokotyl length	2,6	2,9	3,1	3,3	2,5	3,4	2,9	3,0	3,4	3,6	2,8	3,3	3,0	2,9	2,9	3,3	3,4	3,0	2,7	2,9	*3,0*	0,3	9,5	
Control 5																								
Root length	7,0	7,5	5,7	6,4	7,7	6,9	6,4	5,0	5,3	6,6	7,5	6,1	6,0	7,8	4,8	5,2	6,0	6,6	6,5	5,0	*6,3*	0,9	14,5	
Hypokotyl length	2,8	4,0	3,2	2,6	3,4	2,9	3,6	3,8	3,1	2,9	3,3	3,6	2,9	3,2	2,6	3,3	2,6	3,3	3,7	1,9	*3,1*	0,5	15,6	
Control 6																								
Root length	7,4	6,2	7,0	6,7	6,7	6,9	5,9	5,1	5,0	5,6	5,9	5,6	6,0	7,0	7,7	5,5	6,3	6,1	5,4	5,3	6,2	0,8	12,3	
Hypokotyl length	3,5	2,7	2,8	2,6	3,2	2,8	1,8	3,4	2,8	3,9	3,1	2,9	2,8	2,6	3,0	2,4	2,6	3,1	2,5	2,1	2,8	0,5	16,4	
				**C1**	**C2**	**C3**	**C4**	**C5**	**C6**															
Mean Root Length				*5,93*	*6,24*	*6,42*	*6,83*	*6,30*	*6,17*												***6,31***			
Mean Hypokotyl Length				2,72	2,73	2,89	3,05	3,14	2,83												**2,89**			
	**6.25%**																							
Sample 1																					**MW [cm]**	**STD DEV [cm]**	**VAR KOE**	**Inhibition [%]**
Root length	5,8	7,1	7,0	6,6	6,6	5,3	7,1	6,0	5,8	6,9	6,2	5,7	6,5	7,0	5,1	6,0	5,5	5,5	4,5	4,3	*6,03*	0,82	13,55	
Hypokotyl length	2,7	3,5	2,8	3,7	2,9	2,6	3,3	1,9	3,4	2,8	2,8	2,9	2,9	3,3	3,3	2,4	2,6	2,4	2,0	2,9	*2,86*	0,46	16,15	
Sample 2																								
Root length	7,2	6,1	5,9	6,5	5,7	6,8	5,9	5,3	7,1	6,1	7,4	5,9	7,2	7,0	4,7	6,4	7,0	6,9	6,7	6,9	*6,44*	0,70	10,85	
Hypokotyl length	3,0	3,3	2,8	3,3	3,4	3,2	3,5	2,7	2,9	3,4	2,4	3,3	3,6	3,6	3,2	3,2	3,1	3,4	2,5	3,7	*3,18*	0,35	11,09	
Sample 3																								
Root length	5,5	6,4	6,2	5,5	4,3	6,2	7,0	6,5	5,8	5,3	7,2	5,7	6,2	5,7	5,1	5,6	6,5	7,1	6,2	5,5	*5,98*	0,70	11,70	
Hypokotyl length	3,0	3,3	2,4	3,3	3,1	3,4	2,8	3,5	3,3	2,8	3,3	2,9	3,4	2,7	3,3	2,8	3,5	3,6	2,8	2,2	*3,07*	0,37	12,19	
				**S1**	**S2**	**S3**																		
Mean Root Length				*6,03*	*6,44*	*5,98*															**6,15**			**2,67**
Mean Hypokotyl Length				2,86	3,18	3,07															**3,03**			**-4,93**
	**12,5**																							
Sample 1																								
Root length	6,3	6,1	7,1	5,8	7,1	6,1	5,5	4,1	6,1	6,4	5,4	4,7	5,2	5,5	6,1	6,1	6,5	6,7	5,3	6,1	*5,9*	0,7	12,3	
Hypokotyl length	3,8	3,5	3,6	3,7	4,0	3,7	2,6	3,6	3,4	3,5	2,9	3,6	2,9	3,8	2,7	2,7	3,4	3,6	3,0	2,7	3,34	0,43	12,94	
Sample 2																								
Root length	5,8	5,8	6,2	5,5	5,2	6,1	5,0	5,4	5,8	6,1	6,0	5,8	6,1	5,5	6,1	5,2	6,1	6,1	5,3	6,1	*5,76*	0,37	6,44	
Hypokotyl length	3,0	3,0	2,8	2,6	3,1	2,7	2,8	3,4	3,1	2,7	3,1	3,3	2,7	2,9	2,7	3,2	2,7	2,7	2,6	2,7	2,89	0,24	8,18	
Sample 3																								
Root length	5,5	7,3	6,9	6,1	7,2	5,8	5,2	7,3	5,7	6,5	6,6	6,4	6,1	6,1	5,3	7,1	5,8	7,0	7,2	6,0	*6,4*	0,7	10,6	
Hypokotyl length	2,9	3,3	2,9	2,6	3,3	2,8	3,9	3,4	2,5	3,3	2,8	3,1	2,3	3,6	3,5	3,7	3,2	3,0	2,5	2,7	3,07	0,43	13,93	
				**S1**	**S2**	**S3**																		
Mean Root Length				*5,91*	*5,76*	*6,36*															**6,01**			**4,83**
Mean Hypokotyl Length				3,34	2,89	3,07															**3,10**			**-7,12**
	**25%**																							
Sample 1																					**MW [cm]**	**STD DEV [cm]**	**VAR KOE**	**Inhibition [%]**
Root length	6,4	7,3	6,5	5,4	6,0	5,8	5,6	6,2	6,3	6,8	7,2	6,0	6,3	7,8	6,4	6,9	6,6	5,8	6,3	5,0	*6,33*	0,65	10,29	
Hypokotyl length	2,8	3,2	3,2	3,3	3,4	3,2	2,9	3,1	3,8	3,8	3,2	3,3	3,0	3,8	3,4	3,6	2,7	3,1	3,6	3,1	*3,28*	0,31	9,55	
Sample 2																								
Root length	6,9	5,6	5,8	5,0	6,3	7,4	5,1	5,3	6,4	6,7	6,9	7,8	7,9	5,8	5,6	4,9	5,3	5,4	5,8	5,3	*6,06*	0,91	14,94	
Hypokotyl length	3,7	3,1	3,2	3,2	3,3	3,0	2,9	3,4	3,6	3,2	3,4	3,8	3,7	2,9	3,0	2,3	3,3	1,5	2,8	3,0	*3,12*	0,51	16,28	
Sample 3																								
Root length	5,9	6,3	3,9	6,4	4,6	4,6	6,6	6,8	5,5	3,0	4,1	5,5	5,6	6,0	6,1	4,5	5,6	4,6	5,3	4,1	*5,25*	1,00	19,09	
Hypokotyl length	3,1	3,0	3,2	3,3	2,5	3,3	3,4	3,8	3,8	3,2	2,9	2,9	3,4	2,6	3,3	3,2	3,5	3,2	3,7	2,0	*3,17*	0,43	13,53	
				**S1**	**S2**	**S3**																		
Mean Root Length				*6,33*	*6,06*	*5,25*															***5,88***			**6,86**
Mean Hypokotyl Length				3,28	3,12	3,17															**3,19**			**-10,18**

Table S3. Continued

	**50,0%**																							
Sample 1																								
Root length	5,6	4,0	5,9	4,6	5,6	7,6	5,0	4,1	6,4	6,6	6,0	4,1	5,2	6,6	6,6	6,2	5,9	3,9	4,2	6,2	*5,52*	1,05	19,01	
Hypokotyl length	3,1	2,3	3,4	3,8	3,2	4,5	3,7	3,1	3,1	3,5	3,3	3,5	3,0	2,9	3,3	3,3	3,0	3,5	3,9	3,3	*3,34*	0,44	13,05	
Sample 2																								
Root length	4,9	6,2	6,1	5,4	6,2	6,5	6,4	5,2	4,9	6,5	4,9	4,8	5,6	6,5	6,6	6,1	6,1	5,3	6,2	6,0	*5,82*	0,61	10,52	
Hypokotyl length	3,3	4,0	2,7	3,6	3,3	2,8	3,5	3,8	3,7	3,4	3,0	3,2	2,8	3,5	3,1	3,4	3,2	2,3	3,2	2,7	*3,23*	0,41	12,70	
Sample 3																								
Root length	5,6	5,3	5,9	7,3	5,2	6,0	6,8	5,2	5,4	5,1	5,6	5,7	5,2	6,7	6,2	5,9	5,7	5,5	4,6	5,0	*5,70*	0,64	11,32	
Hypokotyl length	3,3	3,4	2,9	3,5	3,2	3,1	4,7	4,0	3,2	3,7	2,8	3,0	3,2	2,7	3,5	3,3	3,6	3,7	3,0	3,7	*3,38*	0,45	13,28	
				**S1**	**S2**	**S3**																		
Mean Root Length				*5,52*	*5,82*	*5,70*															***5,68***			**10,08**
Mean Hypokotyl Length				3,34	3,23	3,38															**3,31**			**-14,56**
	**100%**																							
Sample 1																					**MW [cm]**	**STD DEV [cm]**	**VAR KOE**	**Inhibition [%]**
Root length	4,5	4,7	4,6	4,6	5,3	3,1	3,5	2,2	2,5	3,4	2,1	2,5	4,5	1,9	1,8	1,6	1,6	2,0	1,9	1,7	*3,00*	1,24	41,26	
Hypokotyl length	2,7	2,3	2,3	2,9	1,8	1,6	1,4	1,6	1,6	1,5	1,8	0,7	0,7	1,7	1,4	1,8	2,1	1,4	1,2	0,7	1,66	0,59	35,40	
Sample 2																								
Root length	6,8	6,6	6,3	6,5	6,5	6,9	5,4	4,7	6,2	6,3	6,4	5,7	5,5	5,8	5,9	5,7	5,6	6,2	5,1	5,6	*5,99*	0,57	9,44	
Hypokotyl length	3,2	3,7	3,0	3,2	3,0	3,1	3,1	2,9	2,8	3,1	3,3	2,1	3,3	2,3	3,4	2,9	3,1	3,1	3,0	2,8	*3,02*	0,34	11,36	
Sample 3																								
Root length	5,6	6,5	4,1	5,5	6,9	6,4	6,3	6,3	6,5	6,2	6,6	5,0	6,0	6,5	5,6	5,7	5,1	5,8	6,3	6,1	*5,95*	0,65	10,94	
Hypokotyl length	2,4	3,4	3,3	3,1	3,2	3,4	3,5	2,4	2,8	3,3	3,2	2,8	3,4	3,6	3,0	3,1	3,0	3,0	2,7	3,1	*3,09*	0,33	10,57	
				**S1**	**S2**	**S3**																		
Mean Root Length				*3,00*	*5,99*	*5,95*															***4,98***			***21,15***
Mean Hypokotyl Length				1,66	3,02	3,09															**2,59**			***10,46***

Table S3. Continued

SRRA$_{TD}$-Organic eluat

**LEPIDIUM SATIVUM**																								
Seed Charge:																								
Date:	17.04.2023																							
Name:																								
Sample Name:	Köyiçi organik																							
Control:																								
**KONTROLLEN**																								
Control 1	1	2	3	4	5	6	7	8	9	10	11	12	13	14	15	16	17	18	19	20	**MV [cm]**	**STD DEV [cm]**	**VAR KOE**	
Root length	6,0	6,1	6,5	6,4	6,5	5,9	6,6	5,9	6,7	6,2	5,1	6,3	6,0	5,5	4,9	6,7	5,4	4,9	6,1	4,9	*5,93*	0,60	10,06	
Hypokotyl length	2,3	3,4	2,3	2,8	3,2	3,1	3,4	2,8	3,0	3,1	2,0	2,4	3,0	2,0	2,5	2,7	2,9	3,1	2,1	2,3	2,72	0,44	16,22	
Control 2																								
Root length	7,0	6,0	6,3	7,3	6,9	6,6	7,2	6,7	6,2	5,5	7,0	6,1	5,9	6,0	5,9	5,8	5,4	5,5	5,5	6,0	*6,24*	0,59	9,45	
Hypokotyl length	2,6	3,0	2,9	2,2	2,8	2,1	3,3	3,0	2,9	3,1	3,2	3,3	2,4	2,7	2,7	2,8	2,2	2,4	2,6	2,4	*2,73*	0,36	13,06	
Control 3																								
Root length	6,0	9,0	6,4	7,6	8,5	6,5	6,3	6,3	5,5	5,8	6,8	6,2	6,5	6,9	6,2	6,4	6,0	5,3	4,8	5,4	*6,42*	0,98	15,34	
Hypokotyl length	3,2	3,1	2,8	1,4	3,1	3,3	3,3	2,9	2,5	3,4	2,2	3,5	3,5	3,3	2,9	3,6	2,8	2,8	2,0	2,1	2,89	0,57	19,74	
Control 4																								
Root length	6,4	7,5	6,6	6,3	5,8	7,4	7,7	7,2	7,5	6,8	7,3	5,7	6,0	7,1	6,0	7,6	6,7	7,7	6,8	6,4	*6,83*	0,64	9,38	
Hypokotyl length	2,6	2,9	3,1	3,3	2,5	3,4	2,9	3,0	3,4	3,6	2,8	3,3	3,0	2,9	2,9	3,3	3,4	3,0	2,7	2,9	*3,0*	0,3	9,5	
Control 5																								
Root length	7,0	7,5	5,7	6,4	7,7	6,9	6,4	5,0	5,3	6,6	7,5	6,1	6,0	7,8	4,8	5,2	6,0	6,6	6,5	5,0	*6,3*	0,9	14,5	
Hypokotyl length	2,8	4,0	3,2	2,6	3,4	2,9	3,6	3,8	3,1	2,9	3,3	3,6	2,9	3,2	2,6	3,3	2,6	3,3	3,7	1,9	*3,1*	0,5	15,6	
Control 6																								
Root length	7,4	6,2	7,0	6,7	6,7	6,9	5,9	5,1	5,0	5,6	5,9	5,6	6,0	7,0	7,7	5,5	6,3	6,1	5,4	5,3	6,2	0,8	12,3	
Hypokotyl length	3,5	2,7	2,8	2,6	3,2	2,8	1,8	3,4	2,8	3,9	3,1	2,9	2,8	2,6	3,0	2,4	2,6	3,1	2,5	2,1	2,8	0,5	16,4	
				**C1**	**C2**	**C3**	**C4**	**C5**	**C6**															
Mean Root Length				*5,93*	*6,24*	*6,42*	*6,83*	*6,30*	*6,17*												***6,31***			
Mean Hypokotyl Length				2,72	2,73	2,89	3,05	3,14	2,83												**2,89**			
	**6.25%**																							
Sample 1																					**MW [cm]**	**STD DEV [cm]**	**VAR KOE**	**Inhibition [%]**
Root length	5,8	7,1	7,0	6,6	6,6	5,3	7,1	6,0	5,8	6,9	6,2	5,7	6,5	7,0	5,1	6,0	5,5	5,5	4,5	4,3	*6,03*	0,82	13,55	
Hypokotyl length	2,7	3,5	2,8	3,7	2,9	2,6	3,3	1,9	3,4	2,8	2,8	2,9	2,9	3,3	3,3	2,4	2,6	2,4	2,0	2,9	*2,86*	0,46	16,15	
Sample 2																								
Root length	7,2	6,1	5,9	6,5	5,7	6,8	5,9	5,3	7,1	6,1	7,4	5,9	7,2	7,0	4,7	6,4	7,0	6,9	6,7	6,9	*6,44*	0,70	10,85	
Hypokotyl length	3,0	3,3	2,8	3,3	3,4	3,2	3,5	2,7	2,9	3,4	2,4	3,3	3,6	3,6	3,2	3,2	3,1	3,4	2,5	3,7	*3,18*	0,35	11,09	
Sample 3																								
Root length	5,5	6,4	6,2	5,5	4,3	6,2	7,0	6,5	5,8	5,3	7,2	5,7	6,2	5,7	5,1	5,6	6,5	7,1	6,2	5,5	*5,98*	0,70	11,70	
Hypokotyl length	3,0	3,3	2,4	3,3	3,1	3,4	2,8	3,5	3,3	2,8	3,3	2,9	3,4	2,7	3,3	2,8	3,5	3,6	2,8	2,2	*3,07*	0,37	12,19	
				**S1**	**S2**	**S3**																		
Mean Root Length				*6,03*	*6,44*	*5,98*															**6,15**			**2,67**
Mean Hypokotyl Length				2,86	3,18	3,07															**3,03**			**-4,93**
	**12,5**																							
Sample 1																								
Root length	6,3	6,1	7,1	5,8	7,1	6,1	5,5	4,1	6,1	6,4	5,4	4,7	5,2	5,5	6,1	6,1	6,5	6,7	5,3	6,1	*5,9*	0,7	12,3	
Hypokotyl length	3,8	3,5	3,6	3,7	4,0	3,7	2,6	3,6	3,4	3,5	2,9	3,6	2,9	3,8	2,7	2,7	3,4	3,6	3,0	2,7	3,34	0,43	12,94	
Sample 2																								
Root length	5,8	5,8	6,2	5,5	5,2	6,1	5,0	5,4	5,8	6,1	6,0	5,8	6,1	5,5	6,1	5,2	6,1	6,1	5,3	6,1	*5,76*	0,37	6,44	
Hypokotyl length	3,0	3,0	2,8	2,6	3,1	2,7	2,8	3,4	3,1	2,7	3,1	3,3	2,7	2,9	2,7	3,2	2,7	2,7	2,6	2,7	2,89	0,24	8,18	
Sample 3																								
Root length	5,5	7,3	6,9	6,1	7,2	5,8	5,2	7,3	5,7	6,5	6,6	6,4	6,1	6,1	5,3	7,1	5,8	7,0	7,2	6,0	*6,4*	0,7	10,6	
Hypokotyl length	2,9	3,3	2,9	2,6	3,3	2,8	3,9	3,4	2,5	3,3	2,8	3,1	2,3	3,6	3,5	3,7	3,2	3,0	2,5	2,7	3,07	0,43	13,93	
				**S1**	**S2**	**S3**																		
Mean Root Length				*5,91*	*5,76*	*6,36*															**6,01**			**4,83**
Mean Hypokotyl Length				3,34	2,89	3,07															**3,10**			**-7,12**
	**25%**																							
Sample 1																					**MW [cm]**	**STD DEV [cm]**	**VAR KOE**	**Inhibition [%]**
Root length	6,4	7,3	6,5	5,4	6,0	5,8	5,6	6,2	6,3	6,8	7,2	6,0	6,3	7,8	6,4	6,9	6,6	5,8	6,3	5,0	*6,33*	0,65	10,29	
Hypokotyl length	2,8	3,2	3,2	3,3	3,4	3,2	2,9	3,1	3,8	3,8	3,2	3,3	3,0	3,8	3,4	3,6	2,7	3,1	3,6	3,1	*3,28*	0,31	9,55	
Sample 2																								
Root length	6,9	5,6	5,8	5,0	6,3	7,4	5,1	5,3	6,4	6,7	6,9	7,8	7,9	5,8	5,6	4,9	5,3	5,4	5,8	5,3	*6,06*	0,91	14,94	
Hypokotyl length	3,7	3,1	3,2	3,2	3,3	3,0	2,9	3,4	3,6	3,2	3,4	3,8	3,7	2,9	3,0	2,3	3,3	1,5	2,8	3,0	*3,12*	0,51	16,28	
Sample 3																								
Root length	5,9	6,3	3,9	6,4	4,6	4,6	6,6	6,8	5,5	3,0	4,1	5,5	5,6	6,0	6,1	4,5	5,6	4,6	5,3	4,1	*5,25*	1,00	19,09	
Hypokotyl length	3,1	3,0	3,2	3,3	2,5	3,3	3,4	3,8	3,8	3,2	2,9	2,9	3,4	2,6	3,3	3,2	3,5	3,2	3,7	2,0	*3,17*	0,43	13,53	
				**S1**	**S2**	**S3**																		
Mean Root Length				*6,33*	*6,06*	*5,25*															***5,88***			**6,86**
Mean Hypokotyl Length				3,28	3,12	3,17															**3,19**			**-10,18**

Table S3. Continued

	**50,0%**																							
Sample 1																								
Root length	5,6	4,0	5,9	4,6	5,6	7,6	5,0	4,1	6,4	6,6	6,0	4,1	5,2	6,6	6,6	6,2	5,9	3,9	4,2	6,2	*5,52*	1,05	19,01	
Hypokotyl length	3,1	2,3	3,4	3,8	3,2	4,5	3,7	3,1	3,1	3,5	3,3	3,5	3,0	2,9	3,3	3,3	3,0	3,5	3,9	3,3	*3,34*	0,44	13,05	
Sample 2																								
Root length	4,9	6,2	6,1	5,4	6,2	6,5	6,4	5,2	4,9	6,5	4,9	4,8	5,6	6,5	6,6	6,1	6,1	5,3	6,2	6,0	*5,82*	0,61	10,52	
Hypokotyl length	3,3	4,0	2,7	3,6	3,3	2,8	3,5	3,8	3,7	3,4	3,0	3,2	2,8	3,5	3,1	3,4	3,2	2,3	3,2	2,7	*3,23*	0,41	12,70	
Sample 3																								
Root length	5,6	5,3	5,9	7,3	5,2	6,0	6,8	5,2	5,4	5,1	5,6	5,7	5,2	6,7	6,2	5,9	5,7	5,5	4,6	5,0	*5,70*	0,64	11,32	
Hypokotyl length	3,3	3,4	2,9	3,5	3,2	3,1	4,7	4,0	3,2	3,7	2,8	3,0	3,2	2,7	3,5	3,3	3,6	3,7	3,0	3,7	*3,38*	0,45	13,28	
				**S1**	**S2**	**S3**																		
Mean Root Length				*5,52*	*5,82*	*5,70*															***5,68***			**10,08**
Mean Hypokotyl Length				3,34	3,23	3,38															**3,31**			**-14,56**
	**100%**																							
Sample 1																					**MW [cm]**	**STD DEV [cm]**	**VAR KOE**	**Inhibition [%]**
Root length	4,5	4,7	4,6	4,6	5,3	3,1	3,5	2,2	2,5	3,4	2,1	2,5	4,5	1,9	1,8	1,6	1,6	2,0	1,9	1,7	*3,00*	1,24	41,26	
Hypokotyl length	2,7	2,3	2,3	2,9	1,8	1,6	1,4	1,6	1,6	1,5	1,8	0,7	0,7	1,7	1,4	1,8	2,1	1,4	1,2	0,7	1,66	0,59	35,40	
Sample 2																								
Root length	6,8	6,6	6,3	6,5	6,5	6,9	5,4	4,7	6,2	6,3	6,4	5,7	5,5	5,8	5,9	5,7	5,6	6,2	5,1	5,6	*5,99*	0,57	9,44	
Hypokotyl length	3,2	3,7	3,0	3,2	3,0	3,1	3,1	2,9	2,8	3,1	3,3	2,1	3,3	2,3	3,4	2,9	3,1	3,1	3,0	2,8	*3,02*	0,34	11,36	
Sample 3																								
Root length	5,6	6,5	4,1	5,5	6,9	6,4	6,3	6,3	6,5	6,2	6,6	5,0	6,0	6,5	5,6	5,7	5,1	5,8	6,3	6,1	*5,95*	0,65	10,94	
Hypokotyl length	2,4	3,4	3,3	3,1	3,2	3,4	3,5	2,4	2,8	3,3	3,2	2,8	3,4	3,6	3,0	3,1	3,0	3,0	2,7	3,1	*3,09*	0,33	10,57	
				**S1**	**S2**	**S3**																		
Mean Root Length				*3,00*	*5,99*	*5,95*															***4,98***			***21,15***
Mean Hypokotyl Length				1,66	3,02	3,09															**2,59**			***10,46***

Table S3. Continued

SRRA$_{TD}$- Inorganic eluat

**LEPIDIUM SATIVUM**																								
Seed Charge:																								
Date:	7.04.2023																							
Name:	Ali ÇANKAYA																							
Sample Name:	Köy İçi İnorganik																							
Control:	6																							
**KONTROLLEN**																								
Control 1	1	2	3	4	5	6	7	8	9	10	11	12	13	14	15	16	17	18	19	20	**MV [cm]**	**STD DEV [cm]**	**VAR KOE**	
Root length	4,0	3,3	5,0	3,7	5,1	5,2	3,8	4,7	4,6	3,1	4,0	4,5	3,1	4,6	3,1	4,1	5,0	3,3	4,0	3,3	*4,08*	0,71	17,38	
Hypokotyl length	3,3	3,2	2,6	2,8	2,3	3,0	3,1	3,4	2,6	1,5	2,9	2,7	2,2	3,1	3,0	3,2	2,3	2,6	2,9	3,5	2,81	0,47	16,65	
Control 2																								
Root length	5,3	5,0	4,9	4,5	4,6	4,3	7,1	4,9	5,4	6,2	5,3	6,4	5,3	6,3	4,5	6,1	4,8	6,2	4,3	5,3	*5,34*	0,78	14,59	
Hypokotyl length	2,0	3,0	2,8	2,3	2,1	2,1	3,1	2,9	2,6	2,6	2,8	2,6	1,9	2,9	3,1	2,6	2,5	2,4	3,5	3,2	*2,65*	0,42	15,85	
Control 3																								
Root length	5,1	6,5	6,1	6,9	5,3	4,9	5,0	5,9	4,7	6,2	6,2	4,7	5,0	4,6	5,7	5,0	5,3	6,1	5,0	4,4	*5,43*	0,70	12,80	
Hypokotyl length	2,8	3,1	3,5	3,1	3,7	2,7	2,6	3,0	1,6	3,1	2,9	3,5	3,7	3,3	3,6	2,4	3,5	2,8	3,7	2,6	3,06	0,52	17,06	
Control 4																								
Root length	4,6	4,8	4,7	5,2	6,8	5,0	4,9	5,6	7,7	4,2	5,0	5,0	6,1	3,8	5,6	4,4	3,6	4,6	5,4	3,7	*5,04*	0,98	19,45	
Hypokotyl length	2,9	2,9	2,5	2,8	2,9	2,5	2,7	2,6	3,2	2,7	2,9	3,4	3,0	3,0	3,2	3,0	2,2	3,2	2,3	2,9	*2,8*	0,3	10,6	
Control 5																								
Root length	5,3	4,3	4,7	5,8	3,9	5,3	5,8	3,3	3,6	4,2	5,3	4,6	4,0	3,7	4,3	3,5	5,1	2,5	1,7	2,6	*4,2*	1,1	26,1	
Hypokotyl length	3,5	2,8	3,6	3,1	3,8	3,6	2,1	2,3	2,0	3,3	3,5	3,2	2,6	3,3	2,7	2,9	2,1	2,6	2,2	3,2	*2,9*	0,6	19,1	
Control 6																								
Root length	5,1	5,5	4,7	4,9	6,3	4,1	5,3	6,8	5,6	4,7	4,7	5,3	4,7	5,2	4,6	4,0	5,5	5,4	3,8	4,5	5,0	0,7	14,2	
Hypokotyl length	3,3	2,9	3,2	2,7	3,3	2,5	3,2	3,6	2,5	3,1	2,2	2,7	3,3	2,6	2,7	3,2	3,1	2,6	2,8	2,6	2,9	0,4	12,2	
				**C1**	**C2**	**C3**	**C4**	**C5**	**C6**															
Mean Root Length				*4,08*	*5,34*	*5,43*	*5,04*	*4,18*	*5,04*												***4,85***			
Mean Hypokotyl Length				2,81	2,65	3,06	2,84	2,92	2,91												**2,86**			
	**6.25%**																							
Sample 1																					**MW [cm]**	**STD DEV [cm]**	**VAR KOE**	**Inhibition [%]**
Root length	5,7	4,0	5,4	4,3	4,4	6,3	4,2	4,7	4,7	4,5	5,6	4,7	4,5	4,4	4,1	5,8	5,1	4,4	4,0	4,8	*4,78*	0,64	13,41	
Hypokotyl length	2,7	2,9	3,3	2,9	2,6	2,9	2,6	3,0	2,4	3,1	2,6	2,9	2,7	3,2	2,8	2,7	3,0	3,0	3,8	3,0	*2,91*	0,30	10,30	
Sample 2																								
Root length	4,9	4,7	4,8	4,7	4,0	4,7	4,1	5,3	4,7	4,7	5,1	4,7	4,1	4,5	5,2	4,0	4,7	4,7	4,5	4,9	*4,65*	0,36	7,74	
Hypokotyl length	2,7	3,3	2,7	2,3	2,8	2,8	3,1	2,4	3,2	2,8	3,2	2,8	3,0	3,5	3,0	2,7	2,8	2,8	3,6	3,9	*2,97*	0,38	12,91	
Sample 3																								
Root length	6,3	3,6	3,7	6,2	6,0	3,9	4,1	6,2	4,7	4,1	5,3	3,6	5,0	5,6	5,3	4,8	4,3	4,7	4,7	4,7	*4,84*	0,86	17,85	
Hypokotyl length	3,7	3,3	2,5	3,9	3,1	2,7	3,7	4,2	3,4	3,1	3,0	3,3	2,5	3,3	3,3	3,6	2,7	2,5	2,8	2,8	*3,17*	0,48	15,07	
				**S1**	**S2**	**S3**																		
Mean Root Length				*4,78*	*4,65*	*4,84*															**4,76**			**1,87**
Mean Hypokotyl Length				2,91	2,97	3,17															**3,02**			**-5,27**
	**12,5**																							
Sample 1																								
Root length	4,4	5,8	5,1	4,8	4,5	4,2	5,2	4,1	6,0	3,8	5,1	4,3	6,1	4,5	4,8	4,0	4,5	4,6	4,6	4,6	*4,8*	0,6	13,1	
Hypokotyl length	3,5	2,8	3,3	3,0	2,5	3,2	2,3	3,7	3,0	3,6	3,3	2,7	3,3	2,9	2,8	2,7	2,8	3,4	3,3	3,0	3,06	0,36	11,91	
Sample 2																								
Root length	5,5	4,9	4,0	5,4	3,9	4,9	5,7	4,3	4,3	5,2	4,2	5,2	4,4	4,3	4,0	4,7	4,8	4,4	4,0	3,7	*4,59*	0,57	12,42	
Hypokotyl length	3,3	3,8	2,2	2,9	2,8	3,5	2,6	3,3	3,4	3,2	2,8	3,5	2,7	3,5	3,7	2,4	2,5	3,5	2,2	2,1	3,00	0,53	17,59	
Sample 3																								
Root length	5,0	4,5	4,5	5,3	5,3	5,1	4,9	4,6	5,1	4,3	4,5	5,0	5,5	4,8	4,9	5,6	4,6	5,0	4,4	5,1	*4,9*	0,4	7,4	
Hypokotyl length	3,7	3,8	3,0	3,5	3,1	2,9	3,1	2,8	2,7	3,1	3,0	2,3	2,6	3,3	3,0	3,8	3,7	2,9	3,2	3,2	3,14	0,40	12,65	
				**S1**	**S2**	**S3**																		
Mean Root Length				*4,75*	*4,59*	*4,90*															**4,75**			**2,08**
Mean Hypokotyl Length				3,06	3,00	3,14															**3,06**			**-6,90**
	**25%**																							
Sample 1																					**MW [cm]**	**STD DEV [cm]**	**VAR KOE**	**Inhibition [%]**
Root length	4,9	3,1	5,8	3,3	5,0	5,2	4,7	5,2	4,1	4,0	4,6	3,8	4,3	3,1	5,0	4,0	4,5	4,0	3,5	4,7	*4,34*	0,73	16,86	
Hypokotyl length	3,4	4,4	3,8	3,4	1,8	3,2	3,4	3,3	3,4	2,5	3,2	2,7	3,3	2,4	2,4	3,2	3,4	1,9	3,2	3,0	*3,07*	0,61	19,80	
Sample 2																								
Root length	4,8	4,0	5,0	5,0	4,2	5,0	5,0	5,5	5,3	5,5	5,7	5,0	5,3	5,5	5,0	5,7	5,9	4,7	4,9	5,5	*5,13*	0,47	9,19	
Hypokotyl length	3,2	2,6	3,2	3,4	3,2	3,0	3,9	3,0	3,1	2,9	3,5	3,9	2,9	3,7	3,4	3,2	2,7	2,2	2,9	2,7	*3,13*	0,42	13,41	
Sample 3																								
Root length	5,5	5,4	5,4	4,2	5,0	4,8	4,4	4,7	4,2	4,9	4,5	4,8	4,0	4,2	4,1	4,1	3,8	3,7	3,7	4,6	*4,50*	0,54	12,05	
Hypokotyl length	3,3	3,9	3,1	3,3	3,5	2,3	2,1	1,9	3,0	3,0	3,6	3,0	3,5	3,8	3,4	4,2	3,2	2,8	3,0	2,7	*3,13*	0,57	18,13	
				**S1**	**S2**	**S3**																		
Mean Root Length				*4,34*	*5,13*	*4,50*															***4,66***			**3,97**
Mean Hypokotyl Length				3,07	3,13	3,13															**3,11**			**-8,52**

Table S3. Continued

	**50,0%**																							
Sample 1																								
Root length	3,2	4,6	4,3	5,1	4,8	3,6	5,0	5,1	3,4	5,1	4,3	4,5	4,5	3,4	3,1	3,6	2,6	4,2	5,1	4,0	*4,18*	0,75	18,08	
Hypokotyl length	3,2	3,4	3,4	3,5	3,8	2,8	2,7	3,5	3,7	3,5	2,8	2,8	2,8	3,7	3,3	1,6	2,7	2,5	3,5	2,5	*3,09*	0,54	17,34	
Sample 2																								
Root length	5,1	4,5	5,7	4,6	5,0	3,8	5,0	4,1	4,3	5,0	4,1	4,6	3,8	3,9	4,3	5,0	5,6	3,9	3,7	4,5	*4,53*	0,58	12,85	
Hypokotyl length	3,1	2,7	4,2	3,3	2,4	3,1	2,5	3,0	2,6	2,7	3,8	3,3	3,9	3,8	2,9	3,3	3,5	3,6	3,1	2,8	*3,18*	0,49	15,39	
Sample 3																								
Root length	4,6	4,5	5,3	3,5	3,4	5,8	4,8	4,6	5,8	5,6	3,7	3,0	5,1	5,1	5,1	6,1	3,0	3,4	4,5	4,1	*4,55*	0,94	20,65	
Hypokotyl length	4,8	4,0	4,1	3,8	3,3	3,8	3,5	3,3	3,9	3,5	3,1	2,8	3,5	3,5	3,5	4,0	3,5	3,3	3,3	3,1	*3,58*	0,43	12,07	
				**S1**	**S2**	**S3**																		
Mean Root Length				*4,18*	*4,53*	*4,55*															***4,42***			**8,89**
Mean Hypokotyl Length				3,09	3,18	3,58															**3,28**			**-14,58**
	**100%**																							
Sample 1																					**MW [cm]**	**STD DEV [cm]**	**VAR KOE**	**Inhibition [%]**
Root length	3,4	4,2	4,3	4,4	4,1	4,8	4,5	4,1	4,1	3,7	4,6	3,3	5,6	3,7	3,5	5,2	3,0	3,2	3,4	1,9	*3,95*	0,81	20,57	
Hypokotyl length	3,5	3,4	3,6	3,5	3,0	3,7	3,6	3,4	3,3	2,6	3,5	3,4	3,3	3,6	2,3	3,3	3,1	2,0	3,1	4,0	3,26	0,47	14,37	
Sample 2																								
Root length	5,5	4,6	5,5	4,2	4,1	4,1	3,9	4,5	6,0	5,2	5,0	5,1	4,1	5,1	4,0	5,1	3,5	4,3	5,0	4,4	*4,66*	0,63	13,61	
Hypokotyl length	3,3	4,2	3,5	4,1	3,1	3,1	3,5	3,5	3,3	2,9	3,4	3,8	3,7	2,9	3,5	3,3	3,3	3,3	3,0	2,3	*3,35*	0,42	12,43	
Sample 3																								
Root length	5,9	5,0	3,5	5,3	5,5	5,6	4,3	5,1	4,1	3,7	3,5	5,3	4,6	3,8	4,2	5,3	2,8	4,0	3,7	4,2	*4,47*	0,84	18,71	
Hypokotyl length	3,1	3,2	2,9	3,0	2,6	2,9	3,4	3,5	2,3	2,2	3,1	3,8	2,9	2,1	2,9	3,3	2,9	3,2	2,7	2,4	*2,92*	0,43	14,75	
				**S1**	**S2**	**S3**																		
Mean Root Length				*3,95*	*4,66*	*4,47*															***4,36***			***10,06***
Mean Hypokotyl Length				3,26	3,35	2,92															**3,18**			***-10,91***

Table S3. Continued

SRRA$_{TSP}$-Inorganic eluat

**LEPIDIUM SATIVUM**																								
Seed Charge:																								
Date:																								
Name:	Ali Çankaya																							
Sample Name:	TSP-Köyiçi inorganik 8ml yerli																							
Control:																								
**KONTROLLEN**																								
Control 1	1	2	3	4	5	6	7	8	9	10	11	12	13	14	15	16	17	18	19	20	**MV [cm]**	**STD DEV [cm]**	**VAR KOE**	
Root length	6,7	6,7	5,0	6,5	7,0	7,2	5,8	4,5	5,0	7,3	5,8	5,5	5,3	4,6	6,5	8,5	4,8	5,1	5,8	5,6	*5,96*	1,03	17,32	
Hypokotyl length	3,3	3,2	3,1	3,6	3,2	3,0	3,4	2,7	3,3	3,7	3,6	2,9	3,7	3,5	3,7	3,3	2,8	3,2	3,5	1,7	3,22	0,45	14,08	
Control 2																								
Root length	6,2	6,3	4,0	5,5	7,1	7,2	5,2	6,0	5,8	7,0	5,0	6,2	5,9	5,0	4,8	5,1	6,5	6,0	5,7	4,5	*5,75*	0,85	14,73	
Hypokotyl length	3,3	3,6	3,3	4,0	3,1	3,5	3,0	3,3	3,5	3,2	3,7	4,0	3,2	2,9	2,7	3,5	3,1	3,1	3,3	2,3	*3,28*	0,39	11,99	
Control 3																								
Root length	6,2	5,5	5,1	6,5	5,6	5,3	6,5	7,8	5,3	7,7	5,3	5,9	5,0	4,5	5,4	5,2	5,0	3,0	3,0	3,1	*5,35*	1,27	23,83	
Hypokotyl length	4,1	2,8	2,3	2,6	3,7	3,3	3,4	4,5	3,4	3,2	2,8	3,1	2,7	2,6	2,9	2,5	2,9	2,1	2,7	3,3	3,05	0,57	18,88	
Control 4																								
Root length	6,7	6,7	5,0	6,5	7,0	7,2	5,8	4,5	5,0	7,3	5,8	5,5	5,3	4,6	6,5	8,5	4,8	5,1	5,8	5,6	*5,96*	1,03	17,32	
Hypokotyl length	3,3	3,2	3,1	3,6	3,2	3,0	3,4	2,7	3,3	3,7	3,6	2,9	3,7	3,5	3,7	3,3	2,8	3,2	3,5	1,7	*3,2*	0,5	14,1	
Control 5																								
Root length	6,2	6,3	4,0	5,5	7,1	7,2	5,2	6,0	5,8	7,0	5,0	6,2	5,9	5,0	4,8	5,1	6,5	6,0	5,7	4,5	*5,8*	0,8	14,7	
Hypokotyl length	3,3	3,6	3,3	4,0	3,1	3,5	3,0	3,3	3,5	3,2	3,7	4,0	3,2	2,9	2,7	3,5	3,1	3,1	3,3	2,3	*3,3*	0,4	12,0	
Control 6																								
Root length	6,2	5,5	5,1	6,5	5,6	5,3	6,5	7,8	5,3	7,7	5,3	5,9	5,0	4,5	5,4	5,2	5,0	3,0	3,0	3,1	5,3	1,3	23,8	
Hypokotyl length	4,1	2,8	2,3	2,6	3,7	3,3	3,4	4,5	3,4	3,2	2,8	3,1	2,7	2,6	2,9	2,5	2,9	2,1	2,7	3,3	3,0	0,6	18,9	
				**C1**	**C2**	**C3**	**C4**	**C5**	**C6**															
Mean Root Length				*5,96*	*5,75*	*5,35*	*5,96*	*5,75*	*5,35*												***5,69***			
Mean Hypokotyl Length				3,22	3,28	3,05	3,22	3,28	3,05												**3,18**			
**conc**	6.25%																							
Sample 1																					**MW [cm]**	**STD DEV [cm]**	**VAR KOE**	**Inhibition [%]**
Root length	4,1	7,8	5,2	6,7	6,1	5,6	5,3	4,9	5,7	4,3	5,7	6,7	5,1	5,4	5,2	4,5	5,7	6,7	6,7	6,7	*5,71*	0,93	16,29	
Hypokotyl length	3,6	3,5	3,7	3,3	3,8	3,2	3,4	3,7	3,4	3,1	3,0	3,3	3,5	2,7	2,6	2,9	3,5	3,3	3,3	3,3	*3,31*	0,31	9,45	
Sample 2																								
Root length	5,5	5,5	5,2	6,7	6,0	6,7	4,7	5,0	5,0	4,6	6,7	6,7	4,6	5,0	6,7	5,0	5,0	5,0	5,0	6,7	*5,57*	0,80	14,43	
Hypokotyl length	3,2	3,3	3,3	3,3	3,2	3,3	3,3	3,5	3,5	2,7	3,3	3,3	2,1	2,5	3,3	3,5	3,5	3,5	3,5	3,3	*3,22*	0,36	11,14	
Sample 3																								
Root length	5,4	5,6	6,4	5,7	5,1	4,7	7,2	5,3	5,2	6,4	6,3	5,4	5,7	5,3	4,7	6,1	6,5	4,9	6,8	5,1	*5,69*	0,70	12,25	
Hypokotyl length	2,9	3,0	3,0	2,8	2,8	2,2	3,3	3,0	3,1	3,2	2,2	3,5	2,4	2,4	2,6	3,2	3,0	2,9	3,0	2,6	*2,86*	0,35	12,21	
				**S1**	**S2**	**S3**																		
Mean Root Length				*5,71*	*5,57*	*5,69*															**5,65**			**0,55**
Mean Hypokotyl Length				3,31	3,22	2,86															**3,13**			**1,73**
	12.5%																							
Sample 1																								
Root length	4,6	4,8	4,3	6,0	5,5	6,7	5,7	4,6	4,0	6,5	5,0	3,8	4,5	5,7	3,8	5,3	5,3	5,3	7,0	5,0	*5,2*	0,9	17,3	
Hypokotyl length	3,5	3,2	2,9	2,4	2,6	3,3	2,5	2,8	2,9	2,7	2,8	2,6	2,7	3,0	2,0	3,1	2,6	2,7	3,0	3,3	2,83	0,34	12,14	
Sample 2																								
Root length	7,0	7,3	5,1	6,0	6,1	5,1	8,7	6,7	8,2	5,7	4,8	4,5	4,2	5,8	5,2	7,8	5,5	5,5	5,7	5,1	*6,00*	1,21	20,17	
Hypokotyl length	2,8	3,1	2,6	2,5	2,8	2,3	2,8	2,3	3,0	2,1	3,0	2,3	2,6	2,8	2,6	3,7	3,0	2,0	2,7	3,1	2,71	0,39	14,39	
Sample 3																								
Root length	4,8	6,1	7,1	6,1	4,0	5,5	6,0	5,1	7,6	5,0	6,3	4,6	8,2	5,5	4,5	5,2	5,8	8,2	4,0	4,2	*5,7*	1,3	22,1	
Hypokotyl length	3,1	3,1	2,3	2,6	3,0	3,2	3,9	2,8	3,4	2,8	2,8	2,7	3,0	3,6	2,3	2,6	2,7	3,0	2,7	2,1	2,89	0,43	14,79	
				**S1**	**S2**	**S3**																		
Mean Root Length				*5,17*	*6,00*	*5,69*															**5,62**			**1,14**
Mean Hypokotyl Length				2,83	2,71	2,89															**2,81**			**11,77**
	25%																							
Sample 1																					**MW [cm]**	**STD DEV [cm]**	**VAR KOE**	**Inhibition [%]**
Root length	5,5	6,9	4,3	6,2	5,2	6,3	6,0	6,7	7,8	6,0	5,5	5,6	5,3	6,0	5,7	5,2	5,8	5,6	6,9	5,4	*5,90*	0,75	12,71	
Hypokotyl length	4,0	3,0	3,2	3,5	2,7	2,5	3,0	3,0	3,7	3,0	3,8	3,0	2,7	2,5	2,5	2,5	3,2	2,5	2,9	3,2	*3,02*	0,44	14,68	
Sample 2																								
Root length	6,2	4,6	5,7	5,9	5,7	4,5	4,6	5,9	5,6	6,2	6,2	4,1	5,2	5,6	5,2	5,5	4,3	5,2	5,9	4,7	*5,34*	0,65	12,19	
Hypokotyl length	3,4	3,4	3,5	3,4	2,9	2,3	3,1	3,4	2,6	3,4	3,4	3,5	2,9	3,2	2,9	3,3	2,6	2,9	3,4	3,0	*3,13*	0,34	10,85	
Sample 3																								
Root length	4,5	5,5	6,2	5,6	5,2	8,0	5,7	5,0	6,0	5,3	7,0	5,0	4,8	5,3	4,3	7,1	5,1	5,1	3,8	4,0	*5,43*	1,02	18,80	
Hypokotyl length	3,0	3,7	2,5	2,0	3,2	3,0	2,9	3,0	2,8	3,2	3,0	2,1	2,6	2,7	3,5	3,1	3,0	2,8	3,0	2,2	*2,87*	0,42	14,61	
				**S1**	**S2**	**S3**																		
Mean Root Length				*5,90*	*5,34*	*5,43*															***5,55***			**2,32**
Mean Hypokotyl Length				3,02	3,13	2,87															**3,00**			**5,61**

Table S3. Continued

	50,0%																							
Sample 1																								
Root length	5,7	5,0	6,7	4,5	6,2	6,5	5,0	6,0	5,3	4,7	4,5	5,8	6,0	6,2	7,4	4,6	4,8	7,4	4,5	4,5	*5,57*	0,94	16,89	
Hypokotyl length	2,6	2,3	2,8	2,6	2,3	2,5	2,5	3,1	2,6	3,0	2,9	3,2	3,0	2,9	4,4	2,3	2,8	4,4	2,5	2,3	*2,85*	0,58	20,47	
Sample 2																								
Root length	7,0	4,4	6,2	7,4	4,8	6,0	5,4	5,7	6,0	6,4	5,7	5,0	5,7	5,2	4,6	4,5	4,5	4,7	5,5	5,4	*5,51*	0,82	14,81	
Hypokotyl length	4,1	2,3	3,5	4,4	2,8	3,5	3,3	3,0	2,7	3,0	2,6	2,5	2,6	3,2	3,2	3,5	2,7	2,1	2,5	3,5	*3,05*	0,58	18,88	
Sample 3																								
Root length	4,6	5,5	5,7	4,1	6,0	4,4	5,0	5,5	5,5	5,0	5,5	4,0	7,4	4,4	5,4	7,4	5,5	5,5	5,5	7,4	*5,47*	0,98	17,86	
Hypokotyl length	4,5	3,5	4,2	3,0	4,0	3,8	3,2	2,5	2,5	3,7	2,5	4,3	4,4	3,5	3,6	4,4	2,5	2,5	2,5	4,4	*3,48*	0,75	21,61	
				**S1**	**S2**	**S3**																		
Mean Root Length				*5,57*	*5,51*	*5,47*															***5,51***			**3,05**
Mean Hypokotyl Length				2,85	3,05	3,48															**3,13**			**1,78**
	100%																							
Sample 1																					**MW [cm]**	**STD DEV [cm]**	**VAR KOE**	**Inhibition [%]**
Root length	4,7	4,6	5,2	5,0	4,6	6,3	5,7	5,8	6,3	4,5	5,9	6,3	4,3	4,5	6,2	6,3	4,3	6,3	5,2	5,8	*5,39*	0,75	14,01	
Hypokotyl length	2,2	2,6	2,3	2,7	1,9	3,0	3,3	3,3	3,5	2,7	3,2	3,3	2,9	2,2	3,2	2,4	2,5	2,5	2,5	2,5	2,74	0,44	16,12	
Sample 2																								
Root length	6,4	6,7	7,8	5,5	4,6	5,2	7,2	6,1	5,8	4,9	5,9	6,6	6,5	5,0	6,1	5,0	5,7	4,1	4,0	4,1	*5,66*	1,03	18,13	
Hypokotyl length	4,0	3,4	3,5	3,0	3,5	3,3	2,8	3,3	2,7	3,1	3,3	3,0	3,2	3,5	3,3	2,0	3,0	3,5	1,8	2,5	*3,09*	0,51	16,60	
Sample 3																								
Root length	6,2	4,5	4,3	4,8	6,7	5,9	5,9	6,0	4,2	4,4	6,3	5,7	4,6	6,4	5,3	5,0	6,3	5,1	4,8	3,7	*5,31*	0,86	16,15	
Hypokotyl length	3,2	2,7	3,2	2,8	3,6	3,8	3,3	3,6	3,5	3,1	2,7	3,2	2,9	2,8	2,7	2,0	2,5	2,0	2,8	3,1	*2,98*	0,47	15,90	
				**S1**	**S2**	**S3**																		
Mean Root Length				*5,39*	*5,66*	*5,31*															***5,45***			***4,10***
Mean Hypokotyl Length				2,74	3,09	2,98															**2,93**			***7,86***

Table S3. Continued

SRRA$_{TSP}$-Organic eluat

**LEPIDIUM SATIVUM**																								
Seed Charge:																								
Date:																								
Name:	Ali Çankaya																							
Sample Name:	TSP- Köyiçi Organik 8ml yerli																							
Control:																								
**KONTROLLEN**																								
Control 1	1	2	3	4	5	6	7	8	9	10	11	12	13	14	15	16	17	18	19	20	**MV [cm]**	**STD DEV [cm]**	**VAR KOE**	
Root length	7,1	7,6	6,0	7,7	4,7	8,0	5,6	6,7	6,9	7,2	6,7	7,0	6,4	4,8	6,0	4,7	7,0	7,0	4,6	4,9	*6,33*	1,07	16,95	
Hypokotyl length	2,9	3,7	3,0	2,7	2,5	3,2	2,6	3,2	3,1	3,1	2,7	3,1	2,5	2,8	3,2	2,0	2,6	3,2	2,5	1,9	2,83	0,42	14,97	
Control 2																								
Root length	7,6	7,0	8,1	6,5	7,7	7,1	7,0	8,3	7,5	6,7	6,7	7,5	7,1	5,3	6,3	5,3	6,3	5,6	5,5	6,4	*6,78*	0,87	12,79	
Hypokotyl length	3,1	3,1	3,2	3,3	2,8	2,2	3,3	3,3	2,8	2,5	3,0	2,2	3,2	3,2	3,2	2,3	3,1	2,4	2,7	3,5	*2,92*	0,40	13,55	
Control 3																								
Root length	5,9	6,3	6,7	8,0	6,2	6,5	6,6	6,5	6,0	5,9	7,2	8,6	7,1	6,1	6,1	6,4	7,2	6,4	6,0	6,4	*6,61*	0,69	10,45	
Hypokotyl length	2,5	2,9	3,1	3,0	3,1	2,6	2,9	3,2	3,0	3,0	2,7	2,6	2,7	2,7	2,9	2,5	2,7	2,4	2,2	2,6	2,77	0,26	9,31	
Control 4																								
Root length	7,1	7,6	6,0	7,7	4,7	8,0	5,6	6,7	6,9	7,2	6,7	7,0	6,4	4,8	6,0	4,7	7,0	7,0	4,6	4,9	*6,33*	1,07	16,95	
Hypokotyl length	2,9	3,7	3,0	2,7	2,5	3,2	2,6	3,2	3,1	3,1	2,7	3,1	2,5	2,8	3,2	2,0	2,6	3,2	2,5	1,9	*2,8*	0,4	15,0	
Control 5																								
Root length	7,6	7,0	8,1	6,5	7,7	7,1	7,0	8,3	7,5	6,7	6,7	7,5	7,1	5,3	6,3	5,3	6,3	5,6	5,5	6,4	*6,8*	0,9	12,8	
Hypokotyl length	3,1	3,1	3,2	3,3	2,8	2,2	3,3	3,3	2,8	2,5	3,0	2,2	3,2	3,2	3,2	2,3	3,1	2,4	2,7	3,5	*2,9*	0,4	13,6	
Control 6																								
Root length	5,9	6,3	6,7	8,0	6,2	6,5	6,6	6,5	6,0	5,9	7,2	8,6	7,1	6,1	6,1	6,4	7,2	6,4	6,0	6,4	6,6	0,7	10,5	
Hypokotyl length	2,5	2,9	3,1	3,0	3,1	2,6	2,9	3,2	3,0	3,0	2,7	2,6	2,7	2,7	2,9	2,5	2,7	2,4	2,2	2,6	2,8	0,3	9,3	
				**C1**	**C2**	**C3**	**C4**	**C5**	**C6**															
Mean Root Length				*6,33*	*6,78*	*6,61*	*6,33*	*6,78*	*6,61*												**6,57**			
Mean Hypokotyl Length				2,83	2,92	2,77	2,83	2,92	2,77												**2,84**			
**conc**	6.25%																							
Sample 1																					**MW [cm]**	**STD DEV [cm]**	**VAR KOE**	**Inhibition [%]**
Root length	6,1	6,7	7,2	7,7	6,7	6,3	7,6	5,7	6,1	5,1	5,9	6,1	6,1	6,8	6,7	5,5	6,2	6,1	7,8	4,7	*6,36*	0,80	12,56	
Hypokotyl length	2,3	2,9	3,2	3,2	3,0	2,4	3,3	3,2	2,7	2,3	2,8	2,7	3,2	2,9	2,2	2,3	2,9	2,3	3,4	2,5	*2,79*	0,38	13,78	
Sample 2																								
Root length	6,5	6,9	7,4	6,8	6,3	6,0	6,7	7,3	7,2	6,5	6,8	6,0	6,6	5,8	7,0	7,6	5,6	5,8	5,8	5,6	*6,51*	0,61	9,36	
Hypokotyl length	3,0	2,5	3,3	3,0	3,1	2,8	3,3	3,1	2,7	2,5	3,1	2,2	2,8	2,2	2,7	2,6	2,7	2,1	2,7	2,9	*2,77*	0,34	12,28	
Sample 3																								
Root length	6,1	5,2	6,7	7,5	5,6	7,8	5,3	6,7	6,5	6,7	6,0	4,3	6,8	6,5	5,3	7,0	6,0	7,2	5,2	5,6	*6,20*	0,87	14,10	
Hypokotyl length	3,0	2,7	2,5	3,1	3,7	3,4	2,8	2,8	2,5	2,6	2,9	3,1	2,8	2,3	3,0	2,7	2,7	3,0	2,0	2,7	*2,82*	0,36	12,87	
				**S1**	**S2**	**S3**																		
Mean Root Length				*6,36*	*6,51*	*6,20*															**6,35**			**3,28**
Mean Hypokotyl Length				2,79	2,77	2,82															**2,79**			**1,70**
	12.5%																							
Sample 1																								
Root length	5,7	6,8	5,6	7,2	6,1	6,3	5,6	6,3	7,5	6,3	7,5	6,7	5,0	7,5	4,8	5,6	6,1	5,6	5,3	6,8	*6,2*	0,8	13,0	
Hypokotyl length	3,1	2,7	2,9	3,5	2,7	2,8	3,0	2,8	2,7	2,7	3,1	3,1	2,2	2,7	2,6	2,6	2,7	3,1	2,7	2,4	2,81	0,28	9,99	
Sample 2																								
Root length	7,7	6,2	5,8	7,3	5,5	7,5	6,7	5,5	7,5	6,3	5,7	7,6	7,1	5,9	7,5	7,0	5,5	6,6	5,6	6,5	*6,55*	0,79	11,99	
Hypokotyl length	3,5	3,1	2,7	2,9	2,8	3,1	2,6	2,9	2,7	2,3	2,2	2,5	2,8	2,2	2,9	2,6	2,2	2,6	2,9	2,7	2,71	0,33	12,01	
Sample 3																								
Root length	6,9	6,0	7,1	5,1	8,5	5,4	6,4	5,7	6,6	6,7	5,9	6,3	5,2	6,1	6,3	7,5	5,7	6,2	6,3	5,8	*6,3*	0,8	12,5	
Hypokotyl length	2,9	2,7	3,0	2,1	3,3	2,7	2,6	2,4	3,6	3,2	2,9	2,7	3,0	2,8	2,1	3,0	2,6	2,7	2,7	2,9	2,80	0,35	12,57	
				**S1**	**S2**	**S3**																		
Mean Root Length				*6,22*	*6,55*	*6,29*															**6,35**			**3,35**
Mean Hypokotyl Length				2,81	2,71	2,80															**2,77**			**2,35**
	25%																							
Sample 1																					**MW [cm]**	**STD DEV [cm]**	**VAR KOE**	**Inhibition [%]**
Root length	6,2	8,5	6,0	6,4	6,4	6,3	5,2	6,9	6,5	5,9	6,7	6,9	6,1	6,1	5,2	7,9	5,7	6,0	7,4	6,6	*6,45*	0,79	12,23	
Hypokotyl length	3,4	3,0	3,1	2,8	3,1	2,5	3,0	2,7	3,4	2,6	2,6	2,6	2,7	1,7	2,8	2,9	2,7	2,5	1,9	2,4	*2,72*	0,41	15,10	
Sample 2																								
Root length	6,5	6,6	5,4	7,2	5,5	5,1	6,9	5,6	6,6	6,5	6,5	5,9	6,6	6,9	7,3	5,2	6,6	5,6	5,7	7,5	*6,29*	0,71	11,28	
Hypokotyl length	2,4	3,1	1,7	2,4	2,1	2,0	3,0	2,4	2,5	2,7	2,4	2,4	2,5	2,2	2,8	2,3	2,0	2,4	2,7	2,6	*2,43*	0,33	13,59	
Sample 3																								
Root length	6,1	6,0	6,7	6,0	5,7	5,8	5,5	7,8	5,9	5,6	7,6	6,1	6,7	7,5	6,3	5,9	6,7	5,7	6,3	6,4	*6,32*	0,65	10,35	
Hypokotyl length	3,2	3,1	3,3	3,3	2,8	3,3	2,5	2,9	2,9	2,1	3,0	2,5	2,0	2,2	2,5	2,7	2,2	2,0	2,1	1,5	*2,61*	0,51	19,67	
				**S1**	**S2**	**S3**																		
Mean Root Length				*6,45*	*6,29*	*6,32*															**6,35**			**3,37**
Mean Hypokotyl Length				2,72	2,43	2,61															**2,59**			**8,87**

Table S3. Continued

	50,0%																							
Sample 1																								
Root length	5,0	7,0	7,5	7,6	8,4	6,7	7,4	6,0	6,1	5,1	4,9	7,6	5,2	6,6	7,0	5,2	5,4	6,0	5,7	6,9	*6,37*	1,02	16,04	
Hypokotyl length	2,6	3,1	3,1	3,3	2,6	3,2	2,9	2,4	2,9	2,5	3,0	2,5	3,0	3,7	3,3	2,6	2,9	2,2	2,4	3,1	*2,40*	0,37	15,44	
Sample 2																								
Root length	6,7	5,4	6,9	6,2	7,0	6,9	6,5	8,2	7,4	6,6	7,4	6,4	6,5	6,9	4,7	6,2	5,8	6,2	5,4	5,3	*6,43*	0,81	12,57	
Hypokotyl length	3,3	2,9	3,1	2,9	2,8	2,6	3,4	3,9	3,2	3,0	3,3	2,9	3,8	3,1	3,2	2,2	3,2	2,6	2,2	2,4	*3,00*	0,44	14,83	
Sample 3																								
Root length	7,9	5,9	5,0	6,0	5,9	5,5	7,2	6,2	6,6	6,5	6,6	6,4	6,1	7,0	5,2	6,9	6,5	5,9	6,1	5,5	*6,25*	0,68	10,90	
Hypokotyl length	3,5	3,5	3,4	3,9	3,2	3,7	2,8	3,5	3,7	3,8	3,3	3,7	3,6	3,1	2,0	3,4	2,8	3,1	2,9	3,1	*3,30*	0,44	13,21	
				**S1**	**S2**	**S3**																		
Mean Root Length				*6,37*	*6,43*	*6,25*															***6,35***			**3,40**
Mean Hypokotyl Length				2,40	3,00	3,30															**2,90**			***-2,23***
	100%																							
Sample 1																					**MW [cm]**	**STD DEV [cm]**	**VAR KOE**	**Inhibition [%]**
Root length	7,1	6,1	6,8	6,6	6,3	5,8	5,7	6,1	5,3	7,2	7,1	6,1	7,7	5,5	7,5	5,8	6,2	5,9	5,7	6,5	*6,35*	0,67	10,55	
Hypokotyl length	3,2	2,8	2,3	2,7	2,5	3,0	3,3	2,8	2,8	3,1	3,8	2,8	2,5	2,3	2,9	2,2	2,6	3,1	2,3	2,6	2,78	0,39	13,96	
Sample 2																								
Root length	6,5	7,0	6,0	7,0	6,5	5,5	5,7	6,2	6,3	6,0	6,4	6,3	5,8	6,2	6,3	5,8	7,5	5,7	6,1	6,5	*6,27*	0,48	7,68	
Hypokotyl length	2,5	3,3	3,0	3,2	4,7	3,0	3,0	2,5	2,9	3,1	3,5	3,2	2,8	3,3	3,3	2,7	2,8	3,1	3,3	2,9	*3,11*	0,45	14,53	
Sample 3																								
Root length	8,8	6,6	5,6	6,8	6,6	5,9	5,3	5,5	6,3	5,6	6,2	5,9	6,1	5,9	5,1	5,6	5,1	6,2	5,2	5,0	*5,97*	0,83	13,96	
Hypokotyl length	3,7	2,8	3,1	3,9	2,8	3,0	2,6	2,6	2,7	2,9	1,7	2,0	1,5	1,8	2,8	1,8	2,3	1,6	1,6	1,7	*2,45*	0,69	28,35	
				**S1**	**S2**	**S3**																		
Mean Root Length				*6,35*	*6,27*	*5,97*															***6,19***			***5,73***
Mean Hypokotyl Length				2,78	3,11	2,45															**2,78**			***2,12***

Section 7. FTIR, ICP-OES and GC/MS analyses

GC/MS, ICP-OES, and FTIR analyses were conducted at ILTEK (Selcuk University-ARGE Lab, Konya). Organic species in DCM extracts were qualitatively analyzed using an Agilent gas chromatograph (5977 A) equipped with an HP-5 column (30 m × 0.25 mm, 0.25 µm) and an MS (7890B) detector. Identification of compounds was performed using m/z data in both positive (ESI^+) and negative (ESI^-) modes. A program (the injector and detector temperature is 300 and 325 ^oC, respectively, and the column temperature program is 50°C (5 minutes)-10°C/minute-180°C (3 minutes)-6°C/minute-30°C (5 minutes)-3°C /minute-310 ^{o}C (3 minutes)) capable of separating volatile and semi-volatile species was employed. The helium carrier gas flow rate was 1.8 mL/min, and the injection volume was 1 µL. Element concentrations were measured using ICP-OES (Perkin Elmer-Optima 2100 DV). Acid extraction of elements from quartz filters was conducted using the microwave method (with HNO_3) recommended by the EPA (1999).

Section 8. Validation Proceduru

The solutions used in ICP-OES, GC/MS, and phytotoxicity analyses (extraction/solvent exchange/dissolution) were analyzed similarly to sample analyses (HNO_3, DCM, and DMSO). This procedure was used to determine potential contamination related to sample preparation in the laboratory and to calculate the detection limit (DL). Lab blank values were obtained from the analysis of 5 filter/nitric acid (20 ml of 10 % HNO_3) solutions (without transferring to the areas), while field blank values were obtained through the transfer of GFA filters/HDPE bottles to the field and back to the laboratory for analysis. The std. dev. of lab blank results were calculated, and the detection limits (DL) were determined as three times the std.dev. However, since any compounds were detected in the lab blanks, the ınstrumen's detection limits were used as the detection limits (MDL=IDL). The limits of detection were as follows: Cd and Zn 0.05 µg/L; Cu 0.09 µg/L; Pb 0.6 µg/L; Al, As, Co, Cr, Se, Ni, Mo, Ba 0.5 µg/L; Mn and others, 2 µg/L (n=4).

Certified reference material SRM 2711, which is similar to heavy metal accumulation in the atmosphere, was used and analyzed with the samples. Before the ICP-OES analysis of the samples, 0.5 µg of Be (Internal standard, 1 µg/mL in 10 % HNO_3)) was added to each sample. The recoveries of the target elements were between 85% and 110%. The relative standard deviation between the measured and certified values was %11 for Cd, Cu, and Na and %50 for Pb, while it was %20 for Al, Cr, Zn, and others.The linearity of the calibration curve prepared with reference standards of 0.5, 1, 5, 10, and 50 µg/L was checked daily. In GC/MS, full scan analyses were performed for DCM in the range of 40 to 650 molecular weight in both positive and negative ionization modes. The same method was applied in the analysis of samples and blanks afterward. Peaks in the lab/field blanks in the extracts were eliminated. The structural characterization of the TDS was investigated by scanning with a Bruker spectrometer in the range of 1,700-600 cm-1. 0.01 g of homogenized samples was placed on the ATR crystal. Every sample was scanned by 24 scans with a wavelength range of 4000 to 450 cm-1 and a resolution of 4 cm1.

Section 9. NTA

FTIR-LOI analyses show that SRRA and BA have high WOM. Along with other findings, the relationship between EC and TDS showed that MQA included phenolic chemicals, whereas BA contained carboxylic acid-carboxyl groups. In order to avoid losses and interferences at volatile and semi-volatile species, GFA filters were respectively dried in the freezer. Clean-up and vacuum (solvent exchange, concentration) were not applied. The presence of semi-volatile species prompted us on the selection of solvents that could extract both volatile and semi-volatile species and allow for concentration without vacuum. DCM was selected as the extraction solvent. Full-scan spectra were analyzed using the NIST Mass Spectral Library software.

Section 10. Results of correlation coefficient

Elements (n=8)	Al	As	Ba	Cd	Co	Cr	Cu	Mo	Ni	Pb	Se	Zn	Ca	K	Mg	Mn	Na	P	pH	EC	OM	WOM	WIOM	EOM	WSA	WIA	Inhibisyon - Inorganic Eluat	Inhibisyon - Inorganic Eluat
Al	1,00																											
As	0,31	1,00																										
Ba	0,96	0,05	1,00																									
Cd	0,24	0,95	0,02	1,00																								
Co	0,98	0,49	0,90	0,42	1,00																							
Cr	0,93	0,52	0,86	0,52	0,97	1,00																						
Cu	0,60	0,88	0,41	0,92	0,74	0,82	1,00																					
Mo	0,25	0,95	0,03	1,00	0,43	0,53	0,92	1,00																				
Ni	0,79	0,76	0,65	0,77	0,89	0,95	0,96	0,78	1,00																			
Pb	0,65	0,87	0,47	0,89	0,79	0,86	0,99	0,89	0,98	1,00																		
Se	0,99	0,36	0,95	0,31	0,99	0,97	0,66	0,32	0,84	0,71	1,00																	
Zn	0,97	0,09	1,00	0,05	0,91	0,88	0,44	0,06	0,68	0,51	0,96	1,00																
Ca	0,74	0,08	0,81	0,12	0,73	0,76	0,43	0,12	0,61	0,48	0,78	0,81	1,00															
K	0,98	0,17	0,99	0,11	0,94	0,90	0,49	0,12	0,72	0,56	0,98	0,99	0,81	1,00														
Mg	0,99	0,29	0,97	0,23	0,97	0,94	0,59	0,24	0,79	0,65	0,99	0,98	0,82	0,99	1,00													
Mn	0,49	0,93	0,29	0,96	0,65	0,74	0,99	0,96	0,92	0,98	0,56	0,33	0,36	0,38	0,50	1,00												
Na	0,98	0,17	0,99	0,12	0,94	0,90	0,49	0,13	0,72	0,56	0,98	1,00	0,81	1,00	0,99	0,39	1,00											
P	0,99	0,30	0,97	0,24	0,98	0,94	0,60	0,26	0,80	0,66	1,00	0,98	0,79	0,99	1,00	0,50	0,99	1,00										
pH (n=6)	-0,62	-0,58	-0,41	-0,45	-0,66	-0,59	-0,60	-0,46	-0,62	-0,61	-0,57	-0,43	0,10	-0,46	-0,51	-0,52	-0,46	-0,55	1,00									
EC(n=6)	-0,65	-0,61	-0,45	-0,50	-0,71	-0,66	-0,66	-0,51	-0,70	-0,68	-0,62	-0,47	0,02	-0,51	-0,57	-0,59	-0,51	-0,60	0,98	1,00								
OM(n=6)	0,63	0,42	0,51	0,38	0,67	0,65	0,54	0,39	0,62	0,59	0,61	0,52	0,12	0,54	0,59	0,50	0,54	0,60	-0,82	-0,91	1,00							
WOM(n=6)	0,77	0,50	0,62	0,51	0,80	0,80	0,73	0,52	0,79	0,75	0,76	0,64	0,28	0,65	0,70	0,64	0,65	0,73	-0,96	-0,98	0,86	1,00						
WIOM(n=6)	-0,33	-0,14	-0,30	-0,24	-0,31	-0,35	-0,36	-0,25	-0,36	-0,31	-0,35	-0,30	-0,18	-0,27	-0,25	-0,24	-0,28	-0,32	0,38	0,23	0,02	-0,49	1,00					
EOM(n=5)	0,47	0,43	0,21	-0,92	0,31	-1,00	-1,00	-0,96	-0,90	-0,56	0,50	0,33	-0,91	0,41	-0,11	-0,70	0,43	0,49	#SAYI/0!	#SAYI/0!	-1,00	-1,00	-0,69	1,00				
WSA (n=6)	-0,59	-0,01	-0,64	-0,06	-0,56	-0,57	-0,33	-0,07	-0,46	-0,33	-0,62	-0,64	-0,79	-0,63	-0,61	-0,21	-0,63	-0,62	-0,04	-0,10	-0,07	-0,20	0,75	-1,00	1,00			
WIA (n=6)	-0,29	-0,08	0,04	-0,89	-0,27	-0,51	0,09	-0,65	-0,43	-0,73	-0,05	0,10	0,66	0,13	-0,12	-0,14	0,11	-0,14	0,65	0,82	-0,07	-0,75	-0,50	1,00	-0,95	1,00		
Inhibisyon_Inorganic Eluat (n=5)	0,71	0,72	0,64	-0,81	0,64	-1,00	-0,28	-0,96	-0,98	-0,21	0,82	0,72	-0,22	0,74	0,48	-0,55	0,74	0,71	1,00	1,00	-0,54	-0,75	-0,38	1,00	-0,49	0,66	1,00	
Inhibisyon_Organic Eluat (n=5)	0,81	0,81	0,87	0,44	0,86	-0,12	-0,99	0,09	0,02	0,93	0,70	0,81	-1,00	0,79	0,95	0,73	0,78	0,82	-1,00	-1,00	0,74	0,53	0,85	1,00	0,77	-0,63	0,17	1,00
